# Energy Gap Law-Harnessing Design of Highly Second Near-Infrared Emissive 34π-Annulated Porphyrinoids for In Vivo Imaging

**DOI:** 10.1021/jacs.5c05151

**Published:** 2025-06-11

**Authors:** Yi-Chen Tsai, Yan-Chang Chen, Hsiu-Feng Lu, Kai-Min Chan, Syue-Liang Lin, Pin-Xuan Lin, Ricardas Rotomskis, Simona Steponkiene, Tung-Kung Wu, Ming-Hsien Chan, Ja-an Annie Ho, Yu-Fen Huang, Chao-Ping Hsu, Yang-Hsiang Chan

**Affiliations:** a Department of Applied Chemistry, 34914 National Yang Ming Chiao Tung University, Hsinchu 300, Taiwan, R.O.C.; b Institute of Chemistry, 71552 Academia Sinica, Taipei 115, Taiwan, R.O.C.; c Department of Biomedical Engineering and Environmental Sciences, 34881 National Tsing Hua University, Hsinchu 300, Taiwan, R.O.C.; d Department of Biotechnology and Laboratory Science in Medicine, 34914 National Yang Ming Chiao Tung University, Taipei 112, Taiwan; e Biomedical Physics Laboratory of National Cancer Institute, Baublio 3B, Vilnius LT-08406, Lithuania; f Department of Biological Science, College of Engineering Bioscience, Center for Emergent Functional Matter Science, 34914 National Yang Ming Chiao Tung University, Hsinchu 300, Taiwan, R.O.C.; g Department of Biomedical Imaging and Radiological Sciences, 34914 National Yang Ming Chiao Tung University, Taipei 112, Taiwan; h Department of Biochemical Science and Technology, 33561 National Taiwan University, Taipei 106, Taiwan, R.O.C.; i Institute of Analytical and Environmental Sciences, 34881 National Tsing Hua University, Hsinchu 300, Taiwan, R.O.C.; j School of Pharmacy, College of Pharmacy, Kaohsiung Medical University, Kaohsiung 807, Taiwan, R.O.C.; k National Center for Theoretical Sciences, Taipei 106, Taiwan, R.O.C.; l Center for Emergent Functional Matter Science, 34914 National Yang Ming Chiao Tung University, Hsinchu 300, Taiwan, R.O.C.; m Department of Medicinal and Applied Chemistry, Kaohsiung Medical University, Kaohsiung 807, Taiwan, R.O.C.

## Abstract

NIR-II fluorophores (1000–1700 nm) are pivotal for biomedical imaging, offering deep-tissue penetration and high signal-to-noise ratios but suffer from low quantum yields (QY < 0.01%) beyond 1200 nm. To date, most reported NIR-II small-molecule dyes are derived from polymethine and xanthene frameworks. However, achieving NIR-II chromophores with sufficient QYs remains challenging, as the energy gap law dictates that internal conversion-governed by the emission energy gap and reorganization energy-dominates nonradiative decay. To address this, we designed a novel pseudo-2D molecular framework: 34 π-electron annulated porphyrinoids (Scheme 1), engineered to minimize reorganization energy. These structures achieve emission wavelengths up to 1290 nm with QYs of 1.10–6.14%. Density functional theory (DFT) calculations were performed to unravel the photophysical mechanisms underlying these behaviors, showing that the reorganization energy is as small as 10.5 meV for these dyes, which validates our design. The optimized molecular structures and the stacking geometry of these porphyrinoids in the nanoparticle form were also elaborated by DFT. The intense NIR-II fluorescence (>1200 nm) enables high-resolution in vivo vascular imaging, further enhanced by AI-driven imaging algorithms to significantly improve image quality.

## Graphical abstract

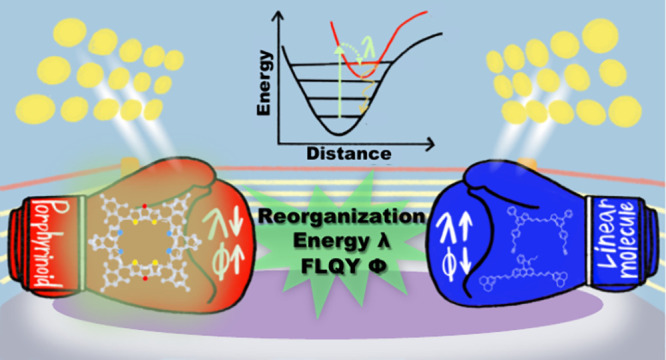

## Introduction

Organic molecules exhibiting second near-infrared (NIR-II, 1000–1700 nm) emission have attracted enormous attention because of their promising applications in guiding surgical resections of tumors in real-time, photoacoustic tomography, photothermal therapy, and complementing other imaging modalities.^1–11^ NIR-II absorbing and emitting fluorophores feature minimal tissue scattering and autofluorescence, deep-tissue penetration (≥10 mm), and enhanced signal-to-noise ratios,^12,13^ rendering them extremely useful in biomedical imaging. More importantly, NIR-II fluorescence imaging has recently achieved groundbreaking advancements in clinical surgeries on humans,^14–16^ although these clinical fluorescence-guided imaging techniques still rely on traditional NIR-I fluorescence using FDA-approved indocyanine green. These achievements highlight the critical need for developing NIR-II dyes with enhanced emission brightness and superior photostability, paving the way for their potential clinical applications.

Unfortunately, achieving appreciable NIR-II fluorescence in organic fluorophores remains highly challenging because of the predominant nonradiative deactivation pathways governed by the energy gap law in systems with a narrow energy gap.^17–20^ Consequently, the currently reported NIR-II fluorescent dyes with emissions exceeding 1200 nm exhibit extremely low quantum yields (QY), typically below 0.01% (Scheme 1).^21–27^ Aiming at circumventing the energy gap law by decreasing the reorganization energy (λ), a recent methodology has been applied to balance lowest-lying transition density within molecules, irrespective of their structural symmetry.^28^ As a result, this method enables intramolecular exciton delocalization with a virtual nodal plane, which successfully suppresses λ and thereby enhances QY.

**Scheme 1:**
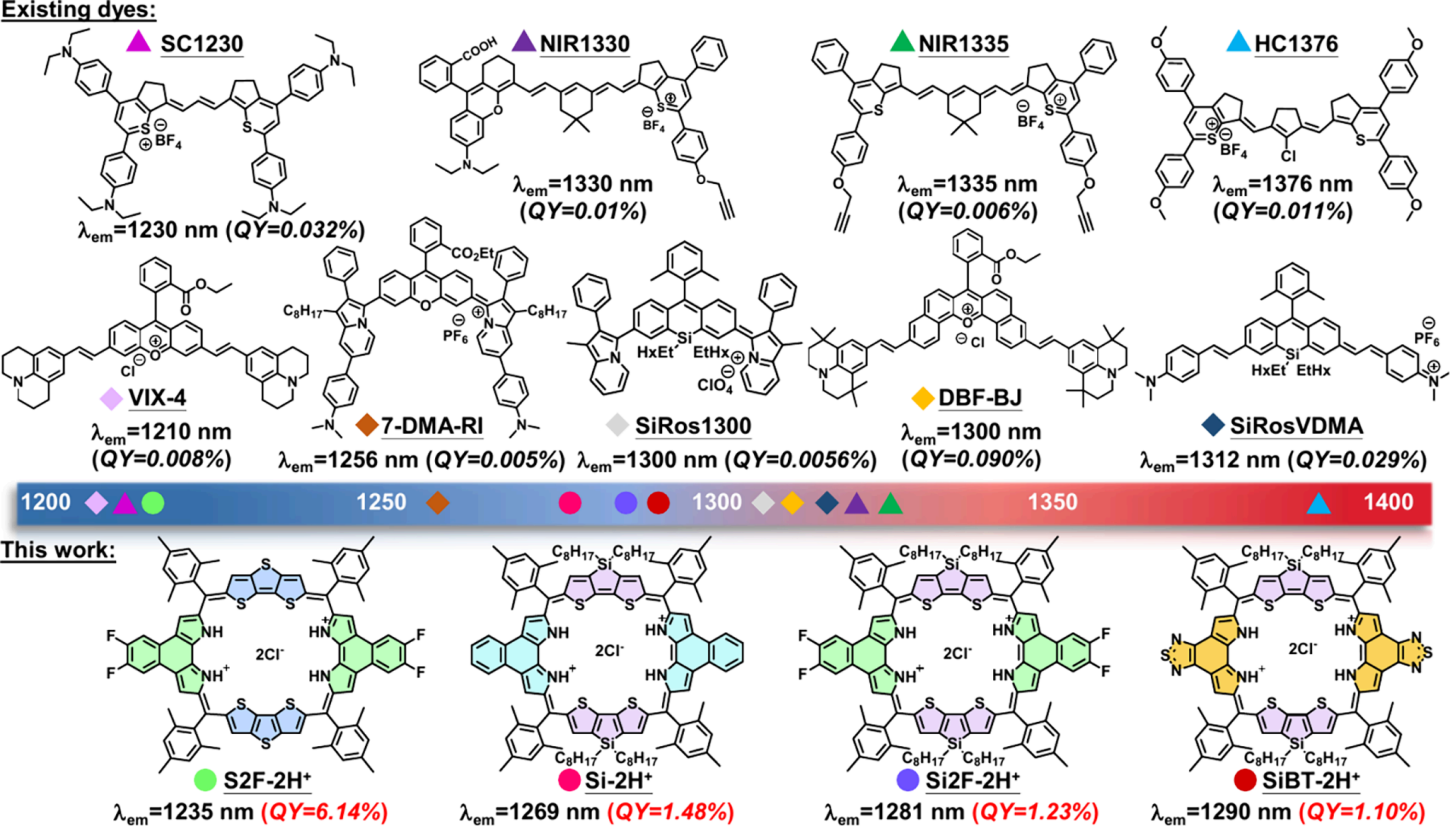
Representative Linear NIR-II Fluorophores, Including Polymethines and Xanthenes with λ_em_ > 1200 nm (Upper Panel) and 2D Porphyrinoids Developed in This Work (Bottom Panel)^a^

a The absorption and fluorescence peak maxima and quantum yields of the reported dyes in organic solvents can refer to SC1230,^28^ NIR1330,^22^ NIR1335,^22^ HC1376,^23^ VIX-4,^24^ 7-DMA-RI,^25^ Si-Ros1300,^26^ DBF-BJ,^22^ and SiRosVDMA.^27^ The QYs were determined using commercially available IR-1061 as a reference (details provided in the text).

Molecules with an odd number of carbon atoms exhibit a nodal plane of transition density centered on the middle carbon atom of the chain. This symmetry allows the transition density to propagate equally in both directions, effectively enabling the exciton to delocalize back and forth along the chain. This process halves the reorganization energy and thereby reduces the internal conversion rate as predicted by the energy gap law. Up to this stage, however, all small organic NIR-II emitters have been based on symmetric or asymmetric 1D linear polymethine and xanthene derivatives featuring intermediates with an odd number of carbon atoms (Scheme 1). Expanding this concept to pseudo-2D molecules, we boldly predicted that if symmetric, porphyrin-like structures with extensive π-electron delocalization can be achieved, significantly lower or even nearly zero reorganization energy (i.e., high QY) is anticipated for such molecules. Reported calculations also indicate that pseudo-2D expanded polycyclic molecules incorporating *D*_*2h*_ building blocks can achieve exceptionally small λ (<100 meV).^29,30^

To experimentally validate the proposed concept, we strategically designed and synthesized a series of porphyrinoids with a 34 π-electron aromatic framework (Scheme 1). First, a large and highly rigid porphyrin analog, **S2F-2H** ^**+**^, was synthesized using dithienothiophene and difluoronaphthobipyrrole as electron donor and acceptor units, respectively. **S2F-2H** ^**+**^ shows a fluorescence maximum at 1235 nm and a QY of 6.14% in CH_2_Cl_2_. To further redshift the emission wavelengths, a stronger electron-donating moiety, silolodithiophene, was introduced, yielding **Si-2H** ^**+**^ and **Si2F-2H** ^**+**^ with NIR-II emission maximized at 1269 and 1281 nm, respectively. Additionally, replacing naphthobipyrrole with a more electron-deficient benzothiazole group produced **SiBT-2H** ^+^, which exhibited an emission peak at 1290 nm and a QY of 1.10% in CH_2_Cl_2_. The resulting NIR-II emissive porphyrinoids exhibit remarkably high QYs ranging from 1.10% to an exceptional 6.14%, which represents a 34-to-183-fold improvement over conventional polymethines and a 38-to-220-fold enhancement compared to the existing xanthenes at similar emission wavelengths. To the best of our knowledge, these porphyrinoids represent the brightest NIR-II dyes reported to date with emissions of >1200 nm. Also, detailed density functional theory (DFT) calculations were performed to elucidate a comprehensive comparison of the photophysical properties of these porphyrinoids, including their Soret and Q bands. These proof-of-concept pseudo-2D porphyrin-like derivatives are utilized for *in vivo* high-contrast NIR-IIa (1300–1400 nm) vascular imaging, with AI-assisted simulations to achieve exceptionally high-resolution vasculature visualization.

## Results and Discussion

Our goal is to harness the energy gap law by reducing reorganization energy, designing highly symmetrical pseudo-2D ring-fused π-conjugated molecules to address the long-standing issue of extremely low QY in NIR-II dyes emitting beyond 1200 nm. Additionally, we introduced variations in donor and acceptor units to extend the fluorescence of porphyrin-like molecules into the NIR-IIa region (1300 nm), as longer wavelengths are advantageous for deep-tissue imaging in biological systems. Furthermore, we performed vascular imaging in live mice, utilizing AI assistance to enhance image resolution.

### Rational Design and Synthesis of 34π-Annulated Porphyrinoids

Scheme 2 illustrates the synthetic pathway for the 34π-annulated porphyrinoids developed in this study. Two types of donors were prepared: silolodithiophene-based (compound **1**) and dithienothiophene-based (compound **2**) derivatives, each featuring two hydroxyl groups on either side. Additionally, two types of acceptors, benzothiazolebipyrrole (compound **9**) and naphthobipyrrole (compound **13**), were synthesized for comparative studies. The synthesis of 5,6-difluoronaphthobipyrrole (compound **19**) involved a Suzuki–Miyaura reaction between 1,2-dibromo-4,5-difluorobenzene and *N*-triisopropylsilyl (TIPS)-protected pyrrole pinacol boronate, yielding compound **15**. Deprotection of the TIPS group using tetrabutylammonium fluoride (TBAF) produced compound **16**, which was subsequently protected with a tert-butoxycarbonyl (Boc) group to form compound **17**. Finally, oxidative coupling followed by Boc deprotection resulted in compound **19**. Lewis acid-catalyzed condensation of compound **1** with compounds **9**/**13**/**19**, or compound **2** with compound **19**, followed by oxidation and acid protonation to afford diprotonated aromatic form^31^ of compounds **SiBT-2H** ^**+**^, **Si-2H** ^**+**^, **Si2F-2H** ^**+**^, and **S2F-2H** ^**+**^, respectively.

**Scheme 2:**
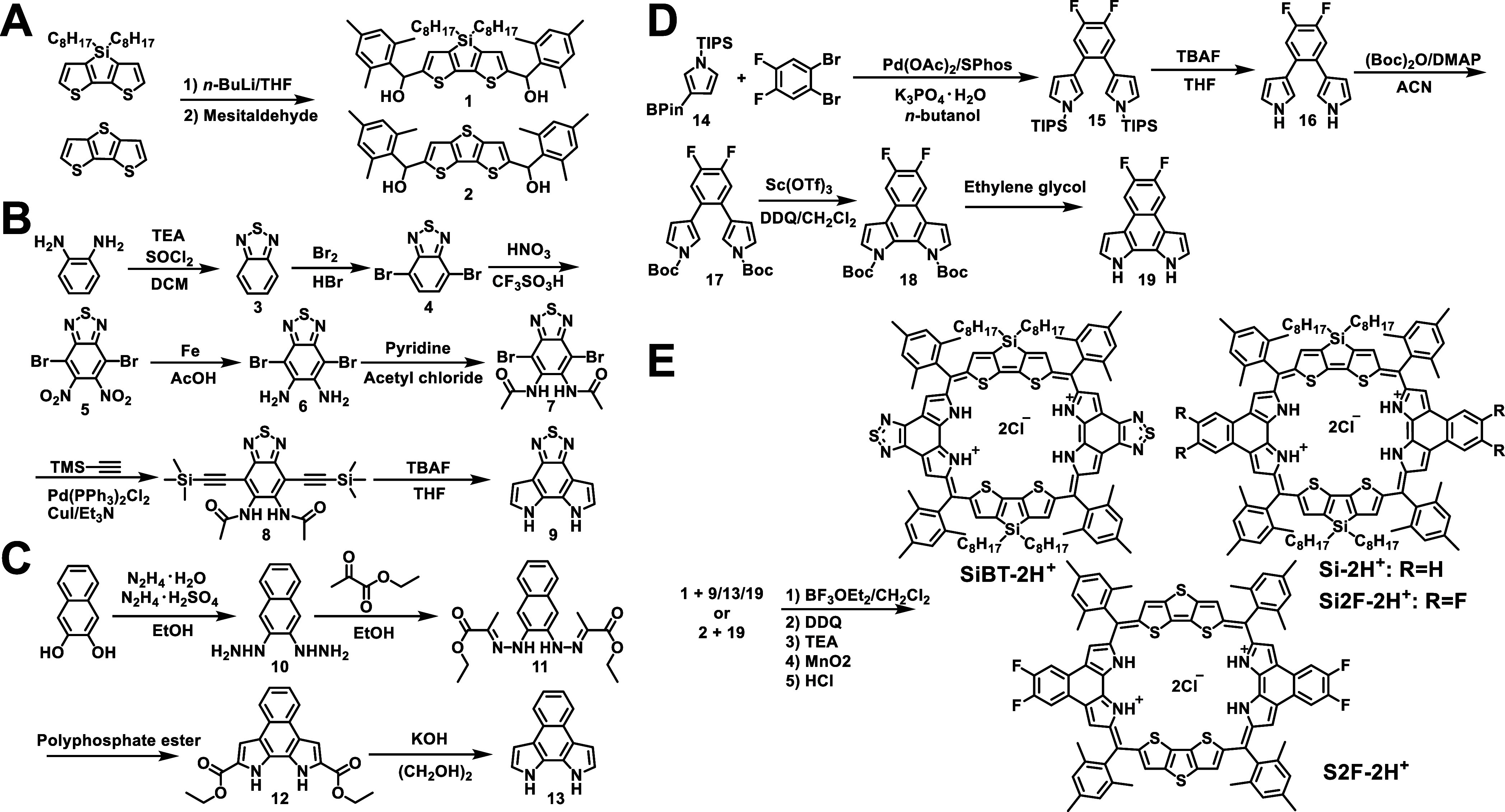
Synthetic routes of (A) donors, (B) benzothiazolebipyrrole-based acceptor, (C) naphthobipyrrole-based acceptor, (D) 5,6-difluoronaphthobipyrrole-based acceptor, and (E) NIR-II emissive 34π-annulated porphyrinoids in this study^a^

a For detailed synthesis and characterization procedures, please refer to the main text and the Supporting Information.

### Photophysical Properties

We successfully synthesized two types of 34π-annulated porphyrinoids (Figure 1A): (i) 5,6-difluoro-substituted naphthobipyrrole acceptor with dithieno- and silolodithiophene donors (**S2F-2H** ^**+**^ and **Si2F-2H** ^**+**^, respectively) and (ii) naphthobipyrrole and benzothiazolebipyrrole acceptors with silolodithiophene donor (**Si-2H** ^**+**^ and **SiBT-2H** ^**+**^, respectively). Figure 1B represents the absorption and emission spectra of these porphyrinoids in CH_2_Cl_2_. All compounds exhibit NIR-II emission beyond 1200 nm, with **SiBT-2H** ^**+**^ demonstrating a peak at 1290 nm, approaching the NIR-IIa region (1300–1400 nm). The extended emission wavelengths confirm the successful integration of a stronger electron-donating unit, silolodithiophene (compared to dithienothiophene), and a more enhanced electron-withdrawing group, benzothiazolebipyrrole (relative to (5,6-difluoro)­naphthobipyrrole). Furthermore, the absorption and emission peaks of **Si-2H** ^**+**^, **Si2F-2H** ^**+**^, and **S2F-2H** ^**+**^ display minimal shifts even in the polar solvent CH_3_CN, whereas **SiBT-2H** ^**+**^ appears to be highly dependent on the solvent polarity (Figures S1 and S2). The results suggest a relatively low reorganization energy, λ, for **Si-2H** ^**+**^, **Si2F-2H** ^**+**^, and **S2F-2H** ^**+**^, despite their noncoplanar geometry (vide infra). In contrast, **SiBT-2H** ^**+**^ shows phenomenal charge transfer properties, resulting in a pronounced redshift in emission wavelength. These porphyrinoids also display distinct absorption coefficients in their Soret and Q bands (Table 1), as elaborated in a later section.

**Figure 1:**
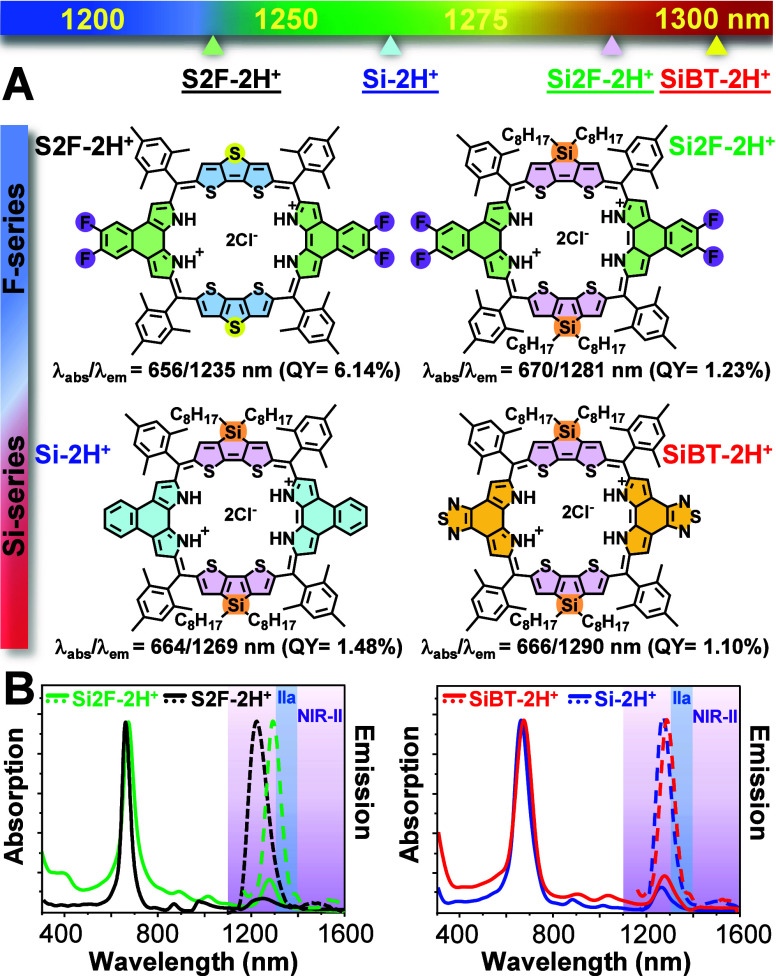
(A) Chemical structures and key optical properties of the studied 34 π-electron annulated porphyrinoids. (B) Their corresponding absorption and emission spectra.

**Table 1:** Photophysical Properties of Experimental Results and Theoretical Calculations of Porphyrinoid Series in CH_2_Cl_2_

	**experimental data**							**TD-PBE**38/6–31G*		
dye	λ_max_^abs^ (nm)^a^	λ_max_^abs^ (nm)^b^	λ_max_^em^ (nm)^c^	ε_max_ (M^–1^ cm^–1^)^d^	ε_max_ (M^–1^ cm^–1^)^e^	Φ^f^ (%)	brightness (cm^2^)^g^	λ_max_^abs^ (nm)^a^	λ_max_^abs^ (nm)^h^	λ_max_^em^ (nm)^c^
**Si-2H** ^**+**^	664	885, 1035, 1259	1269	1.52 × 10^4^	0.30 × 10^4^	1.48	224.96	633 (*f* ^i^ = 3.7982)	1243 (*f* = 0.4129)	1311
**SiBT-2H** ^**+**^	666	900, 1075, 1279	1290	3.71 × 10^4^	2.08 × 10^4^	1.10	408.10	625 (*f* = 3.7294)	1261 (*f* = 0.4975)	1340
**Si2F-2H** ^**+**^	670	899, 1013, 1272	1281	1.01 × 10^4^	0.50 × 10^4^	1.23	124.23	631 (*f* = 3.8037)	1258 (*f* = 0.4417)	1332
**S2F-2H** ^**+**^	656	864, 971, 1234	1235	3.57 × 10^4^	1.52 × 10^4^	6.14	2191.98	606 (*f* = 4.3523)^j^	1150 (*f* = 0.3974)^j^	1181
								615 (*f* = 4.1340)^k^	1190 (*f* = 0.4119)^k^	1263^k^

a Absorption maximum of Soret band.

b Absorption maximum of Q-band.

c Emission maximum.

d Absorption coefficient of Soret band.

e Absorption coefficient of the 3rd Q-band.

f Fluorescence quantum yield.

g Absorption coefficient (ε) x quantum yield (Φ).

h Absorption maximum of the 1st band.

i Oscillator strength (*f*).

j Wave-shaped structure.

k Bowl-shaped structure.

We employed the commercially available NIR-II dye IR-1061 (emission peak: 1090 nm in CH_2_Cl_2_) as a reference to quantify the relative QY of the study's dyes. The absolute quantum yield of IR-1061 is documented at 0.59% in CH_2_Cl_2_ after the correction for reabsorption/re-emission effects.^32^ The QYs of **Si-2H** ^**+**^, **Si2F-2H** ^+^, and **SiBT-2H** ^**+**^ were determined to be 1.48, 1.23, and 1.10%, respectively. Surprisingly, **S2F-2H** ^**+**^ exhibited a markedly higher QY of 6.14%, a value nearly 2 orders of magnitude greater than those of reported NIR-II fluorophores (Scheme 1). To elaborate on the underlying mechanisms and gain more fundamental insight into the structural-electronic relationships, we conducted comprehensive density functional theory (DFT) simulations for these porphyrinoid series.

### DFT Calculation

#### Conformation and Energetics

We performed DFT calculations and found that **Si-2H** ^**+**^, **Si2F-2H** ^**+**^, **SiBT-2H** ^**+**^, and **S2F-2H** ^**+**^ adopt a single bowl-shaped configuration in the absence of counterions (Figure S3). However, when paired with chloride anion in a charge-neutral state,^33^ these dyes exhibit two distinct conformations: bowl-shaped and wave-shaped (Figure 2). The BSSE-corrected binding energies of chloride ions and the dyes in both CH_2_Cl_2_ (ε = 8.93) are higher than 38 kcal/mol (Table S1), indicating the strong tendency of chloride association. The most electropositive region, as indicated by molecular electrostatic potential maps (MEP, Figure S4) is the indole hydrogens of the diprotonated dyes, between which the chloride anions are positioned in their energy optimized structure, indicating a maximized electrostatic stabilization. With the known problems of TDDFT, a charge-neutral model has the advantage of being more complete.^33^ As listed in Table 1, the absorption bands calculated by TDDFT are indeed closer to experimental observations for neutral systems containing dichloride ions (Tables 1 and Table S2, a test of different density functionals is in Table S3.) The clear difference in the preferential conformation is seen between **S2F-2H** ^**+**^ and the Si series is also seen in their energies: the wave-shaped conformation is important for **S2F-2H** ^**+**^ but not the Si series (Figure 2). Thermodynamic analysis at 300 K predicts bowl-shaped conformation populations of 97.68 (**Si-2H** ^**+**^), 99.19 (**SiBT-2H** ^**+**^), 97.68 (**Si2F-2H** ^**+**^), and 42.92% (**S2F-2H** ^**+**^). Therefore, the Si-series compounds are represented by their dominant bowl-shaped geometries in the subsequent discussion. In contrast, **S2F-2H** ^**+**^ is more likely in its wave-shaped configurations.

**Figure 2:**
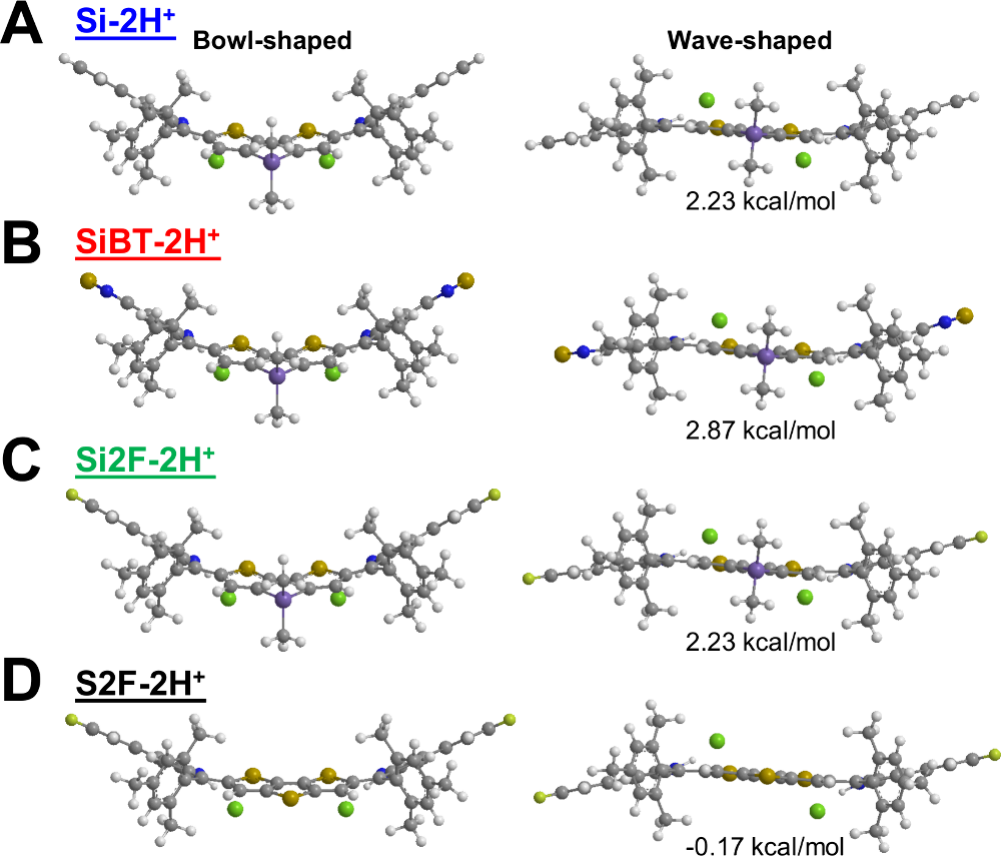
Optimized structures of (A) **Si-2H** ^**+**^, (B) **SiBT-2H** ^**+**^, (C) **Si2F-2H** ^**+**^, and (D) **S2F-2H** ^**+**^ with two chloride ions at the level of B3LYP-D3/6–31G*. The energies indicated are the relative to their bowl-shaped counterpart, calculated at the level of M06–2*X*/6–311+G**.

We further carried out the excited-state calculations using the PBE38 functional with the 6–31G* basis set, and the results are summarized in Table 1 alongside the experimental data. Both theoretical and experimental findings consistently indicate that the absorption and emission wavelengths of **S2F-2H** ^**+**^ are shorter than those of the silicon (Si) series. For the Si series, the order of the Q-band and emission wavelength remains relatively consistent: **SiBT-2H** ^**+**^ > **Si2F-2H** ^**+**^ > **Si-2H** ^**+**^. However, the Soret band displays an opposing trend in theoretical calculations compared to experimental observations, where the experimental ordering diverges. Despite discrepancies between theory and experiment, the energy differences in wavelengths across the Si series are not pronounced. Experimental results reveal a maximum Soret band difference of 7 meV (664 nm vs 670 nm) and a Q-band difference of 16 meV (1269 nm vs 1290 nm). In contrast, theoretical calculations show differences of 25 meV (633 nm vs 625 nm) for the Soret band and 14 meV (1243 nm vs 1261 nm) for the Q-band. These results highlight subtle variations in wavelength behavior between experimental and computational approaches. To gain more insight, we further calculated the highest occupied molecular orbital (HOMO) and lowest unoccupied molecular orbital (LUMO)­of these porphyrinoids. The benzothiazole group in **SiBT-2H** ^**+**^ and fluorine in **S2F-2H** ^**+**^ act as electron-withdrawing groups, which lower the LUMO energy of the dye and narrow the HOMO–LUMO energy gap (*E*_g_). This reduction in *E*_g_ typically shifts the absorption and emission wavelengths of the dye to longer wavelengths (redshift). In contrast, the silole ring in the Si series serves as an exceptionally strong electron donor, endowing these compounds with a significantly lower-lying LUMO compared to **S2F-2H** ^**+**^.

### Theoretical Insights for the Emission QY

QY is inversely related to the rate of internal conversion (IC) process, a radiationless transition process that depletes the excited population without emission. Through the energy gap law,^17^ the rate of IC is inevitably high for NIR transitions, due to the small energy gaps. However, IC rates can be suppressed by a small reorganization energy (λ), as the exponential decay dependence in the energy gap law is mainly due to the vibronic progression of the high-frequency modes; a small displacement in the ground and excited state leads to a much faster decay. A similar conclusion can be drawn from the Marcus theory,^34^ which assumes classical statistics for all the vibrations. In systems with smaller λ, the drop-in transition rate is faster in the inverted region. Since λ is related to the structural change in the transition, it can be computed using the following expression:

$$\lambda^{\mathrm{ground}}=E_{S0@S1}-E_{S0@S0}$$ 1

$$\lambda^{\mathrm{ex}}=E_{S1@S0}-E_{S1@S1}$$ 2

$$\lambda =\left(l^{\mathrm{ground}}+l^{\mathrm{ex}}\right)/2$$ 3

In eq 1, *E*_S0@S1_ and *E*_S0@S0_ denote the ground-state energy evaluated at the S1-optimized geometry and the S0-optimized geometry, respectively. Similarly, *E*_S1@S0_ and *E*_S1@S1_ in eq 2 represent the first excited-state energy calculated at the S0- and S1-optimized structures. The reorganization energy (λ) is is as typically represented by the average of both quantities. In the optimized excited-state (S_1_) structures with TDDFT (B3LYP-D3/6–31G*), we found that the Si-series compounds exclusively retained a bowl-shaped geometry, as attempts to stabilize a wave-shaped conformation were unsuccessful. On the other hand, for **S2F-2H** **^+^,** both bowl- and wave-shaped conformations can be obtained for the S_1_ state (Figure S5). As summarized in Table 2, all compounds exhibit remarkably low λ values (<30 meV). This confirms the great success of the pseudo-2D 34π-annulated porphyrinoid design in minimizing structural reorganization during electronic transitions. The λ trend follows: **SiBT-2H** ^**+**^ > **Si2F-2H** ^**+**^ > **Si-2H** ^**+**^ > **S2F-2H** ^**+**^ (major conformation). Intriguingly, this order inversely correlates with the QY observed experimentally in CH_2_Cl_2_, which is 1.10, 1.23, 1.48, and 6.14%. The generally low λ values confirm the success of this 34π-annulated porphyrinoid design in minimizing structural reorganization during electronic transitions. The observed inverse relationship between QY and λ highlights the critical role of suppressed structural reorganization in enhancing emission efficiency, as lower λ directly corresponds to higher QY. To the best of our knowledge, this is the lowest λ achieved so far for NIR-II fluorophores with emission >1200 nm.

**Table 2:** Reorganization Energies (meV) of **Si-2H** ^**+**^, **SiBT-2H** ^**+**^, **Si2F-2H** ^+^, and **S2F-2H** ^**+**^ at the B3LYP-D3/6-31G* Level

dye		λ^ground^	λ^εx^	λ
**Si-2H** ^**+**^		27	24	25.5
**SiBT-2H** ^**+**^		29	25	27.0
**Si2F-2H** ^**+**^		28	25	26.5
**S2F-2H** ^**+**^	major^a^	11	10	10.5
	minor^b^	31	27	29.0

a Wave-shaped structure.

b Bowl-shaped structure.

The reorganization energy can be further broken into contributions of normal modes.^35^ Unfortunately, systems with anions cannot be analyzed. As listed in Table S4, diprotonated dyes also have very low reorganization energy. The λ for bowl-shaped **S2F-2H** ^**+**^ is still larger than that of the Si system. Thus, we take **Si-2H** ^**+**^ and **S2F-2H** ^**+**^ for the normal-mode analysis (Figure S6). Both show that a major contribution comes from a very low frequency mode, where the molecule bends up and down. Another contribution from the high-frequency region (∼3047 cm^–1^) is the C–H stretching mode of the terminal methyl group, especially for **S2F-2H** ^**+**^. We note that the commonly seen alternating C–C stretching modes for aromatic molecules do not contribute significantly at all, indicating the physical origin of the current molecular design of very low λ and elevated emission QY.

The calculated *E*_g_, molecular orbital energy levels (eV), and orbital shapes are illustrated in Figure 3. **S2F-2H** ^**+**^ exhibits a larger *E*_g_ than the Si series, which correlates with its absorption peak occurring at a shorter wavelength and is consistent with the experimental data. Furthermore, the wave-shaped **S2F-2H** ^**+**^ structure possesses a larger *E*_g_ than its bowl-shaped counterpart, leading to a further blueshift in absorption. Among the Si series, **SiBT-2H** ^**+**^ (*E*_g_ = 1.601 eV) displays the longest absorption wavelength, while **Si-2H** ^**+**^ (*E*_g_ = 1.660 eV) exhibits the shortest. Theoretical oscillator strengths (*f*) confirm that **SiBT-2H** ^**+**^ has the strongest Q-band absorption, whereas **S2F-2H** ^**+**^ shows the weakest absorption intensity. These computational results align precisely with experimental observations, validating the significant interplay between the electronic structures, *E*_g_, and optical properties.

**Figure 3:**
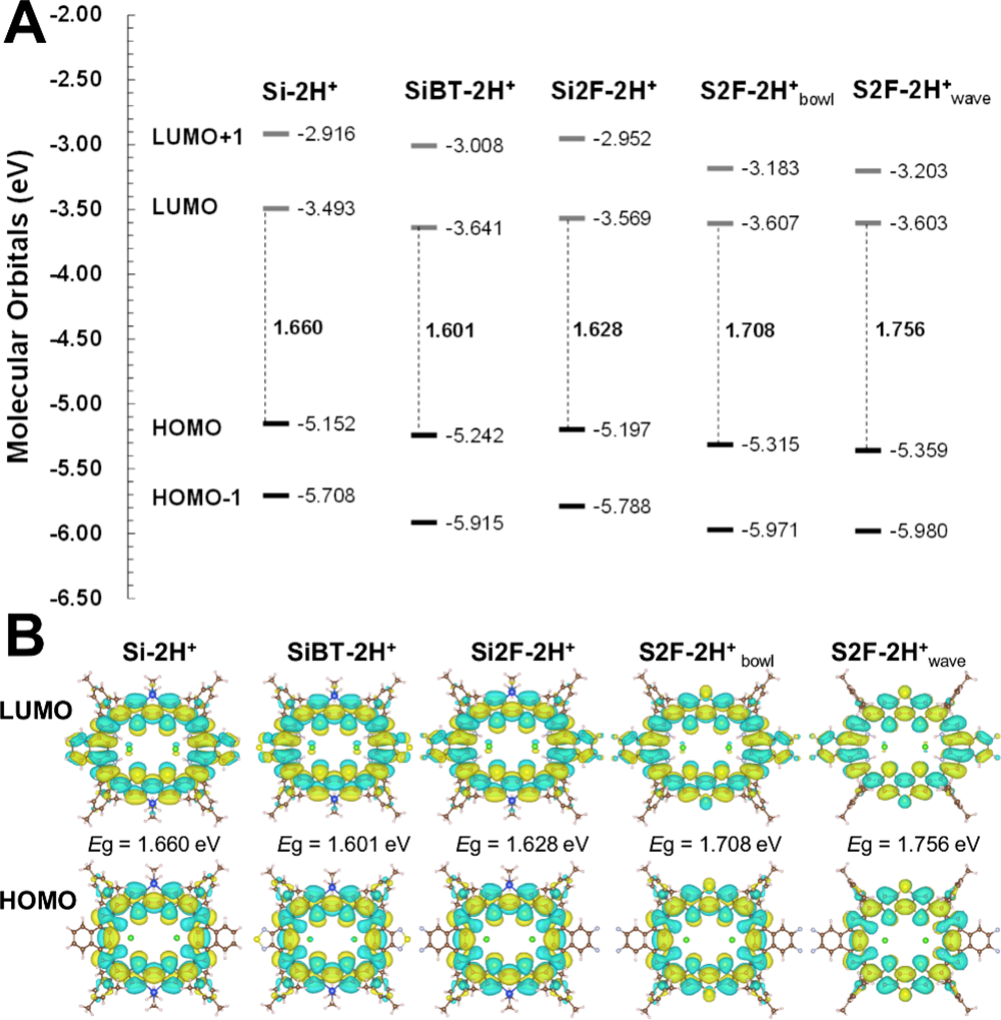
(A) Molecular orbital energy diagram of **Si-2H** ^**+**^, **SiBT-2H** ^**+**^, **Si2F-2H** ^**+**^, **S2F-2H** ^**+**^. Both bowl-shaped and wave-shaped of **S2F-2H** ^**+**^ were included, calculated at the PBE38/6–31G* level in CH_2_Cl_2_ CPCM solvent. HOMO–LUMO energy gaps are shown in the middle. (B) Their corresponding HOMO and LUMO contour surfaces.

### Preparation of Pdots and Investigation of Their Optical Properties

We further demonstrate the use of these porphyrinoids for *in vivo* NIR-II imaging. Four dyes were prepared as semiconducting polymer dots (Pdots) in pure aqueous solution via nanoprecipitation, as illustrated in Figure 4A. We synthesized and employed the sterically hindered conjugated polymer, **Pttc-4OTTQ** (Schemes S1 and S2) as a matrix to enclose porphyrinoids. Previous studies confirm that the **Pttc-4OTTQ** framework effectively suppresses dye aggregation within Pdots.^22,28^ Optical characterization of the porphyrinoid-loaded Pdots (Figure 4B, Table 3) revealed distinct spectral shifts (blue or red) in absorption and emission relative to their unencapsulated counterparts in CH_2_Cl_2_. These shifts can be attributed to polymer–dye molecular interactions and are further rationalized through DFT modeling of electronic structure dynamics.

**Figure 4:**
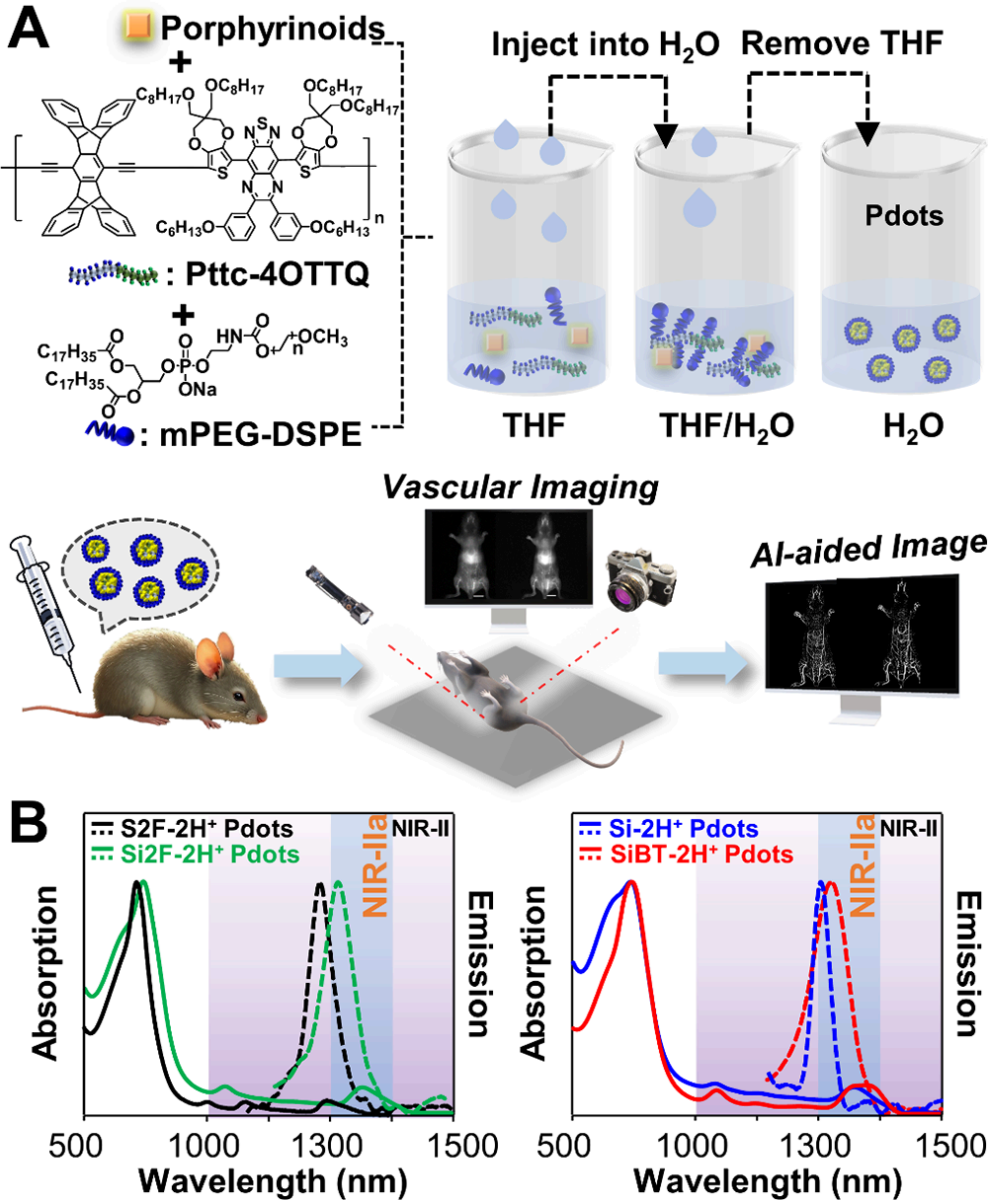
(A) Schematic illustration of the preparation of Pdots for vascular imaging in mice with the aid of AI techniques. (B) Absorption (solid lines) and emission (dashed lines) of **S2F-2H** ^**+**^(black lines), **Si2F-2H** ^**+**^ (green lines), **Si-2H** ^**+**^ (blue lines), and **SiBT-2H** ^**+**^ (red lines) Pdots in H_2_O.

**Table 3:** Summary of Optical Properties of Porphyrinoid-Based Pdots in Water

Pdots	λ_max_^abs^ (nm)	λ_max_^em^ (nm)^c^	S_2_–Stokes shift (nm)^d^	size (nm)^e^	Φ (%)
**Si-2H** ^**+**^	664^a^/1263^b^	1271	607	53	0.71
**SiBT-2H** ^**+**^	628^a^/1279^b^	1280	652	62	0.95
**Si2F-2H** ^**+**^	667^a^/1257^b^	1278	611	57	0.80
**S2F-2H** ^**+**^	668^a^/1218^b^	1220	552	65	1.79

a Absorption maximum of Soret band.

b Absorption maximum of the 3rd Q-band.

c Emission maximum.

d Stokes shift corresponding to Soret band.

e Hydrodynamic size.

The experimental method of making Pdots involves the competition of binding between the dyes, **Pttc-4OTTQ**, PEG-DSPE, and water. In order to further gain qualitative insights for the spectral change for the dyes in Pdots, a highly simplified stacking model can be illuminating, while maintaining the efficiency in the study.^36^ To model the structural interactions of dyes within Pdots, we hypothesize that the **Pttc-4OTTQ** copolymer coassembles with the dye, influencing the dye’s conformation. Within this copolymer, the Pttc unit is relatively inert and sterically bulky, and it possibly exhibits minimal stabilization energy with the dye. Thus, we studied the stacking interactions between the **4OTTQ** moiety and the dye (Scheme S3). With detailed results outlined in the Supporting Information, we found that most of the stacked structures found in Si-containing systems exhibit blueshift in excitation and larger HOMO–LUMO gaps, while the case of **S2F-2H** ^**+**^ shows a smaller band gap and redshift relative to their original undistorted structure. While our ensemble structures are rather limited, given the size of our system, the observed trend suggests that structural change in forming Pdots can be the main reason behind the spectral change.

### *In Vivo* Whole-Body Imaging in Mice with Pdots

We initially compared the fluorescence brightness of **S2F-2H** ^**+**^ and **SiBT-2H** ^**+**^ Pdots by varying long-pass filters (Figure 5 **A-B**) because **S2F-2H** ^**+**^ has the highest emission brightness while **SiBT-2H** ^**+**^ exhibits the longest emission wavelength. At the same Pdot concentration and under the same light power of 1064 nm illumination, we clearly observed that **S2F-2H** ^**+**^ Pdots outperform **SiBT-2H** ^**+**^ Pdots at shorter emission wavelengths (e.g., 1200 nm, Figure 5B,C). Notably, both samples exhibited signal saturation on the InGaAs camera when imaged with a 1100 nm filter, which was attributed to the high laser power (100 mW cm^–2^) required to detect photons through the 1400 nm filter. Under a 1400 nm long-pass filter (LPF), the emission intensity of **SiBT-2H** ^**+**^ Pdots is about 4 times higher than that of **S2F-2H** ^**+**^ Pdots. This highlights the importance of long emission wavelengths, which are advantageous for deep-tissue imaging with a high signal-to-background ratio (SBR). Based on these results, **SiBT-2H** ^**+**^ Pdots were prioritized for subsequent bioimaging studies.

**Figure 5:**
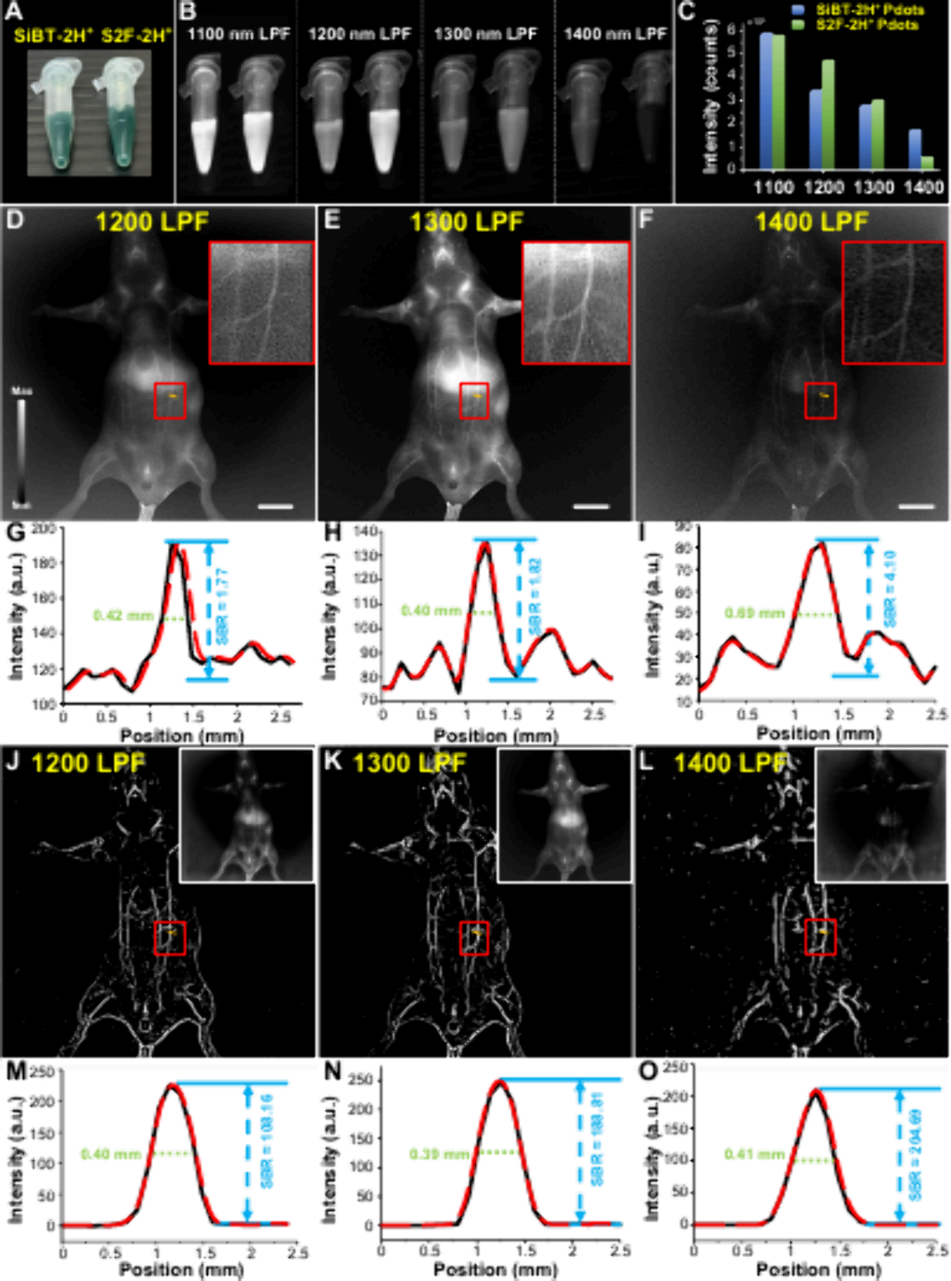
Comparison of (A) **SiBT-2H** ^**+**^ and **S2F-2H** ^**+**^ Pdots in water at the same concentration of 2 mg/mL under (B) irradiation of 1064 nm laser (100 mW cm^–2^) with different LPFs. (C) Mean fluorescence intensities of **SiBT-2H** ^**+**^ and **S2F-2H** ^**+**^ Pdots in (B). Whole-body fluorescence imaging of vascular structures in mice at the supine position injected by **SiBT-2H** ^**+**^ Pdots with a (D) 1200, (E) 1300, and (F) 1400 nm LPF, respectively. The scale bar is 10 mm. (G–I) Their corresponding cross-sectional intensities along the orange lines in (D–F). (J–L) AI-enhanced NIR-II images based on the original images in (D–F) and their corresponding (M–O) cross-sectional intensities along the orange lines in (J–L).

Prior to *in vivo* administration, we first assessed the cytotoxicity and photostability of **SiBT-2H** ^**+**^ Pdots. In vitro/vivo cytotoxicity assays (Figure S8) confirmed their minimal cellular toxicity, while exposure to 254 nm UV irradiation revealed exceptional resistance to photodegradation (Figure S9). Following these validations, **SiBT-2H** ^**+**^ Pdots were administered via tail vein injection into live mice, followed by real-time whole-body imaging using 1064 nm laser excitation. Pdots distributed in the body within 5 min postinjection, allowing high-resolution visualization of vasculature (Figure 5D–F). The SBR of **SiBT-2H** ^**+**^ Pdots on the main blood vessel was measured to be 1.77 with the use of a 1200 nm LPF (Figure 5D), and improved to 1.82 and 4.10 for 1300 and 1400 nm LPF, respectively (Figure 5E,F). This trend is consistent with the reduced background autofluorescence at longer wavelengths. Despite the superior SBR achieved with the 1400 nm filter, a marked decline in emission intensity of **SiBT-2H** ^**+**^ Pdots compromised the image clarity. This issue remains to be addressed for optimal deep-tissue imaging applications.

### Utilization of AI Processing Technologies in Imaging

In an effort to address the aforementioned challenge, we employed AI processing technology to enhance the image quality. The in vivo NIR-II fluorescence imaging data presented in Figure 5J (1200 nm LPF), Figure 5K (1300 nm LPF), and Figure 5L (1400 nm LPF) were processed using a CycleGAN deep-learning model^11,37,38^ and further enhanced with a Frangi vesselness filter^39^ to improve vascular contrast and structural clarity. The integration of deep learning and vessel enhancement algorithms provided a significant improvement in imaging resolution, contrast, and SBR, particularly in longer-wavelength NIR-II imaging. The application of CycleGAN enabled the transformation of standard NIR-IIa images into high-fidelity representations resembling NIR-IIb imaging, effectively reducing light scattering and enhancing contrast. (Figure S11A) The Frangi vesselness filter was subsequently applied to highlight tubular structures, further improving the visibility of fine blood vessels and enhancing feature clarity (Figure S11B). This combination of deep learning and computational imaging techniques facilitated a more accurate delineation of vascular structures compared to unprocessed NIR-IIa images. Quantitative analysis of the fluorescence intensity profiles in panels Figure 5M–O demonstrated the progressive enhancement of image quality across different long-pass filter conditions. At 1200 nm LPF, the vessel structures were well-defined, with a full-width at half-maximum (fwhm) of 0.40 mm and an SBR of approximately 108.16. However, some background noise remained, which limited the overall contrast improvement. At 1300 nm LPF, a slight reduction in fwhm to 0.39 mm suggested improved spatial resolution, while the SBR increased significantly to approximately 188.81. This indicates reduced background interference and sharper vascular definition at 1300 nm LPF as compared to 1200 nm LPF. At 1400 nm LPF, the highest SBR of approximately 204.69 was achieved while maintaining a fwhm of 0.41 mm. The results suggest that the deep-learning enhancement combined with vessel filtering is particularly effective at longer wavelengths where light scattering is minimized. These results demonstrate the promising potential of artificial intelligence-assisted NIR-II imaging for high-resolution vascular imaging, lymphatic mapping, and tumor margin delineation. The observed improvements in contrast and resolution confirm that CycleGAN-based enhancement can bridge the gap between conventional NIR-II imaging and high-performance NIR-IIb imaging, allowing the use of organic fluorophores to achieve imaging performance comparable to toxic inorganic probes. Future work should focus on further optimizing the deep-learning models and validating their applicability in diverse preclinical and clinical imaging scenarios to facilitate broader adoption of this technology in biomedical research and clinical practice.

## Conclusions

Overcoming the energy gap law is critical for designing ultrabright NIR-II emitters, as the energy gap aligning with the second overtone (n = 3) of high-frequency C–H vibrations accelerates nonradiative decay via internal conversion. This process is highly dependent on vibrational reorganization energy λ (eq 3). To address this, we introduce porphyrin-like emitters as a novel class of NIR-II fluorophores engineered to minimize λ through rational molecular design. The concept lies in reducing λ by strategically synthesizing pseudo-2D 34π-annulated porphyrinoids that consist of dithienothiophene/silolodithiophene as the donor and naphthobipyrrole/benzothiazole as the acceptor. Bearing this aim, **Si-2H** ^**+**^, **SiBT-2H** ^**+**^, **Si2F-2H** ^**+**^, and **S2F-2H** ^**+**^ were obtained. The proof of concept is supported by experimental evidence showing a very small Stokes shift (<11 nm) and ultralow computed λ < 29 meV, which confirms suppressed nonradiative pathways. The ultrahigh QY of **S2F-2H** ^**+**^ (6.14%) and long emission wavelength of **SiBT-2H** ^**+**^ (1290 nm) surpass conventional polymethine and xanthene derivatives. DFT calculations revealed how structural conformations (e.g., bowl-shaped vs wave-shaped) and stacking modes (e.g., a-stacked vs b-stacked) govern the optical properties. **S2F-2H** ^**+**^ was further prepared as Pdots to successfully demonstrate its *in vivo* noninvasive fluorescence imaging of vasculature structures. We believe that this design paradigm is generalizable to other porphyrin derivatives to unlock pathways for advanced NIR-II/III emitters in precision bioimaging, diagnostics, and therapeutic monitoring. By decoupling brightness from energy gap law limitations, this work bridges molecular engineering and NIR-II bioimaging.

## Supplementary Material



## Notes

Details of experimental section, including synthetic procedures, characterization, computational approach, photophysical measurements, and AI-imaging (PDF)
