# Supplementary material for: Energy Gap Law-Harnessing Design of Highly Second Near-Infrared Emissive 34π-Annulated Porphyrinoids for In Vivo Imaging
Source: J Am Chem Soc. 2025 Jun 11;147(25):21940–9. doi: 10.1021/jacs.5c05151 (PMC12203586; doi:10.1021/jacs.5c05151)
Supplement: Supplementary file 1 [file ja5c05151_si_001.pdf]

Energy Gap Law-Harnessing Design of Highly NIR-II Emissive 34π-Annulated Porphyrinoids for in Vivo Imaging

Yi-Chen Tsai,[a†] Yan-Chang Chen,[a†] Hsiu-Feng Lu,[b†] Kai-Min Chan,[c] Syue-Liang Lin,[d] Pin-Xuan Lin,[a] Ricardas Rotomskis,[e] Simona Steponkiene,[e] Tung-Kung Wu,[f] Ming-Hsien Chan,[g] Ja-an Annie Ho,[h] Yu-Fen Huang,[c,i,j] Chao-Ping Hsu,[b,k]* and Yang-Hsiang Chan[a,l,m]**

[a]*Department of Applied Chemistry, National Yang Ming Chiao Tung University, Hsinchu, 300, Taiwan, R.O.C.*
[b]*Institute of Chemistry, Academia Sinica, Taipei, 115, Taiwan, R.O.C.*
[c]*Department of Biomedical Engineering and Environmental Sciences, National Tsing Hua University, Hsinchu, 300, Taiwan, R.O.C.*
[d]*Biomedical Department of Biotechnology and Laboratory Science in Medicine, National Yang Ming Chiao Tung University, Taipei, Taiwan*
[e]*Biomedical Physics Laboratory of National Cancer Institute, Baublio 3B, LT-08406, Vilnius, Lithuania*
[f]*Department of Biological Science, College of Engineering Bioscience, Center for Emergent Functional Matter Science, National Yang Ming Chiao Tung University, Hsinchu, 300, Taiwan, R.O.C.*
[g]*Department of Biomedical Imaging and Radiological Sciences, National Yang Ming Chiao Tung University, Taipei, Taiwan*
[h]*Department of Biochemical Science and Technology, National Taiwan University, Taipei, 106, Taiwan, R.O.C.*
[i]*Institute of Analytical and Environmental Sciences, National Tsing Hua University, Hsinchu, 300, Taiwan, R.O.C.*
[j]*School of Pharmacy, College of Pharmacy, Kaohsiung Medical University, Kaohsiung 807, Taiwan, R.O.C.*
[k]*National Center for Theoretical Sciences, Taipei, 106, Taiwan, R.O.C.*
[l]*Center for Emergent Functional Matter Science, National Yang Ming Chiao Tung University, Hsinchu, 300, Taiwan, R.O.C.*
[m]*Department of Medicinal and Applied Chemistry, Kaohsiung Medical University, Kaohsiung, 807, Taiwan, R.O.C.*

† Authors contributed equally to this work.

Supporting Information

Table of Contents

Table of Contents......S2
Experimental Procedures......S3
Scheme S1......S7
Scheme S2......S9
NMR Spectra... ......S14
Figure S1......S34
Figure S2......S34
Computational Details- Detailed analysis of Pdot modeled dyes......S35
Scheme S3......S36
Table S1... ......S37
Table S2... ......S37
Table S3... ......S37
Table S4... ......S37
Table S5... ......S38
Figure S3......S38
Figure S4......S38
Figure S5......S39
Figure S6......S39
Scheme S4......S39
Figure S7......S41
Figure S8......S41
Figure S9......S42
Figure S10......S42
Figure S11......S43
Figure S12......S44
Figure S13......S45
Figure S14......S46
Figure S15......S47
Figure S16......S47
References......S48

Experimental Procedures

Materials. The chemicals used in the experiments were purchased from Alfa Aesar, Sigma-Aldrich, TCI, and Acros. All chemicals were used as received unless described otherwise. DSPE-based lipid mPEG-DPSE (M_w=2000) is obtained from Laysan Bio, Inc. High-purity water (18.2 MΩ•cm) was used throughout the experiment. All ^{1}HNMR and ^{13}CNMR spectra were recorded on Agilent 400-MR DD2 or JEOL JNM-ECZ400S/L1 spectrometers. Compounds **2**,[1] **3-6**,[2,3] **10-13**,[1] **14-19**,[4] **20-21**,[5,6] and **Pttc**[7] were synthesized as reported before.

Synthesis of (4,4-dioctyl-4H-silolo[3,2-b:4,5-b']dithiophene-2,6-diyl)bis(mesitylmethanol), [**1**]. 4,4-dioctyl-4H-silolo[3,2-b:4,5-b']dithiophene (200 mg, 0.48 mmol) was placed into a round-bottom flask under N_2 atmosphere with 8 mL of dry THF. The mixture was cooled to -78 °C and 0.57 mL of *n*-BuLi (2.5 M, 1.44 mmol) was added dropwise and stirred for 1 h. After that, 2,4,6-trimethylbenzaldehyde (215 mg, 1.45 mmol) was added slowly at -78 °C and then heated to 70 °C for stirring for another 5 h. After the reaction, THF was evaporated by a rotary evaporator and then CH_2Cl_2 was added to extract with brine three times. After dried by anhydrous $MgSO_4$, the solvent was removed under reduced pressure and the product was purified by column chromatography on silica gel with hexane/ethyl acetate (20:1, v/v) as eluent to afford 153 mg (44%) of compound **1** as yellow liquid. ^{1}H NMR (400 MHz, $CDCl_3$) δ = 6.87 (s, 4H), 6.53 (dq, J = 8.6, 1.3 Hz, 2H), 6.43 (d, J = 4.3 Hz, 2H), 2.34 (s, 12H), 2.30 (s, 6H), 1.32 – 1.27 (m, 3H), 1.22 – 1.06 (m, 17H), 0.85 – 0.77 (m, 10H), 0.72 – 0.64 (m, 6H). ^{13}C NMR (101 MHz, $(CD_3)_2SO$) δ = 148.56, 147.60, 141.59, 137.50, 136.74, 135.68, 130.06, 126.30, 35.65, 28.90, 28.64, 28.58, 22.88, 20.94, 20.79, 20.55, 20.38, 20.23, 17.59, 17.55, 14.16, 14.05, 10.68. HRMS (FD, $[M]^+$) for $C_{44}H_{62}O_2SiS_2$ 714.39660, found: 714.39624.

Synthesis of N,N'-(4,7-dibromobenzo[c][1,2,5]thiadiazole-5,6-diyl)diacetamide, [7]. Compound **6** (200 mg, 1 mmol) was added in a two-neck round-bottom flask under N_2 atmosphere and 10 mL of dry CH_2Cl_2 was added to the flask, followed by the slow addition of pyridine (0.15 mL, 1.85 mmol) via a dropping funnel at 0 °C. After stirring for 10 min, acetic chloride (0.13 mL, 1.85 mmol) was added slowly and let the mixture to warm up to room temperature for stirring for 5 h. After the reaction, the resulting mixture was extracted using CH_2Cl_2 and brine, followed by drying over $MgSO_4$. The product was further purified by reprecipitation using hexane/ethyl acetate (3:1 v/v) to afford 43 mg (18%) of compound **6** as a yellow solid. ^{1}H NMR (400 MHz, $(CD_3)_2SO$) δ = 10.00 (s, 2H), 2.08 (s, 6H). ^{13}C NMR (101 MHz, $(CD_3)_2SO$) δ = 171.50, 145.68, 145.28, 122.34, 26.28. HR-MS (ESI): calculated for $C_{10}H_9Br_2N_4O_2S$ $[M+H]^+$: 406.8807 , found, 408.8787.

*Synthesis of N,N'-(4,7-bis((trimethylsilyl)ethynyl)benzo[c][1,2,5]thiadiazole-5,6-diyl)diacetamide, [**8**]*. Compound **7** (50 mg, 0.12 mmol), $PdCl_2(PPh_3)_2$ (8.4 mg, 0.012mmol), CuI (4.6 mg, 0.024 mmol), and PPh_3 (3.14 mg, 0.012 mmol) were added in a two-neck round-bottom flask under N_2 atmosphere, followed by the addition of dry triethylamine (2 mL, 0.06 M). Tetramethylsilane (32 mg, 0.36 mmol) was added dropwise into the mixture and was then heated to 80 ^{o}C for 5 h. After the reaction, the mixture was filtered through a celite pad and extracted using ethyl acetate/brine for three times. After drying over $MgSO_4$, the solution was evaporated by a rotary evaporator and purified by column chromatography on silica gel with hexane/ethyl acetate (2:1, v/v) as eluent to get 15 mg (28%) of compound **8** as a white feather-like solid. ^{1}H NMR (400 MHz, $CDCl_3$) δ = 8.12 (s, 2H), 2.25 (s, 6H), 0.34 (s, 18H). ^{13}C NMR (101 MHz, $CDCl_3$) δ 169.21, 152.26, 132.11, 132.01, 131.97, 128.57, 128.45, 110.37, 96.90, 23.75. HRMS (FD, $[M]^+$) for $C_{20}H_{26}N_4O_2Si_2S$ calcd. 442.13095 found: 442.13176.

*Synthesis of 6,7-dihydropyrrolo[3,2-g][1,2,5]thiadiazolo[3,4-e]indole, [**9**].* Compound **8** (20 mg, 0.04 mmol) was added in a two-neck round-bottom flask under N_2 atmosphere, followed by the addition of 4 mL dry THF and tetrabutylammonium fluoride (1M, 0.06 mL). The reaction was heated to 70 ^{o}C for 8 h. After cooling to room temperature, the reaction mixture was added with ethyl acetate and then extracted with brine three times. After dried by anhydrous $MgSO_4$, the solvent was removed under reduced pressure and the product was purified by column chromatography on silica gel with ethyl acetate/CH_2Cl_2 (1:20, v/v) as eluent to afford 4 g (46%) of compound **9** as a yellow solid. ^{1}H NMR (400 MHz, $CDCl_3$) δ = 10.74 (s, 2H), 7.28 (s, 2H), 7.02 (s, 2H). ^{13}C NMR (101 MHz, $(CD_3)_2CO$) δ = 205.30, 149.67, 129.66, 125.17, 121.05, 113.68, 104.29, 28.93. HRMS (FD, $[M]^+$) for $C_{10}H_6N_4S$ calcd. 214.03077 found: 214.03011.

*Synthesis of 3-(4,4,5,5-tetramethyl-1,3,2-dioxaborolan-2-yl)-1-(triisopropylsilyl)-1H-pyrrole, [**14**].*[4] 3-Bromo-1-(triisopropylsilyl)pyrrole (1.46 g, 4.83 mmol) and 20 mL of dry THF were added into a round-bottom flask under N_2 atmosphere. The mixture was cooled to -78 ^{o}C and 3.4 mL of *n*-BuLi (1.6 M, 5.44 mmol) was added dropwise and stirred for 1 h. After that, 2-isopropoxy-4,4,5,5-tetramethyl-1,3,2-dioxaborolane (1.2 mL, 5.92 mmol) was added slowly at -78 ^{o}C and then warmed up to room temperature for stirring for another 8 h. After the reaction, THF was evaporated by a rotary evaporator and then CH_2Cl_2 was added to extract with brine three times. After dried by anhydrous $MgSO_4$, the solvent was removed under reduced pressure, resulting in the formation of a crude product. The crude product was further purified by column chromatography on silica gel using ethyl acetate/CH_2Cl_2 (1:20, v/v) as eluent to yield 1.10 g (66%) of compound **14** as colorless viscous liquid. ^{1}H NMR (400 MHz, $CDCl_3$) δ = 7.23 (dd, *J* = 1.8, 1.3 Hz, 1H), 6.81 (dd, *J* = 2.7, 1.9 Hz, 1H),

6.62 (dd, J = 2.7, 1.3 Hz, 1H), 1.46 (m, J = 15.0, 7.6 Hz, 4H), 1.32 (s, 12H), 1.09 (d, J = 7.5 Hz, 18H).

General procedures for the synthesis of compounds ***Si-BT-2H$^+$, Si-2H$^+$, Si2F-2H$^+$, S2F-2H$^+$, S-2H$^+$, SBT-2H$^+$.*** Compound **1** or **2** (0.14 mmol) and compound **9** or **13** or **19** (0.14 mmol) were dissolved in 200 mL of CH_2Cl_2 was placed in a round-bottom flask. Boron trifluoride (0.07 mmol) was quickly added into the mixture and color of the solution turned purple immediately. The reaction was stirred for 3 h at room temperature and the color of the solution changed to deep blue gradually. After that, 2,3-dichloro-5,6-dicyano-*p*-benzoquinone (0.42 mmol) was added and allowed the reaction to proceed under air for 1 h. Subsequently, triethylamine (0.62 mmol) was added and stirred for 5 min. The mixture was filtered through a neutral Al_2O_3 flash column and then an excess amount of MnO_2 (approximately 1 g) was added and allowed the reaction to proceed under air for 30 min. The residual MnO_2 was filtered through a celite pad to obtain a dark purple solution. The crude product was further purified using silica gel column chromatography with MeOH/CH_2Cl_2 (1:100, v/v) as eluent. Afterward, ethanolic HCl (2 drops in 1 mL ethanol) was added and stirred for 30 min to obtain the reduced product, while the reaction was monitored via TLC. Finally, the solvent was removed under reduced pressure and then washed with HPLC-grade hexane, and further re-precipitated with DCM/MeOH to obtain a deep blue solid with a golden sheen.

Compound **SiBT-2H$^+$** was obtained as a blue solid (7 mg, 3%). ^{1}H NMR (600 MHz, CD_2Cl_2) δ = 11.68 (s, 4H), δ 10.96 (s, 2H), 7.94 (s, 9H), 7.48 (s, 4H), 3.10 (s, 13H), 2.30 (s, 24H), 2.01 (s, 13H), 1.54 (s, 21H), 1.42–1.38 (m, 12H), 0.88 (d, J = 7.4 Hz, 25H). ^{13}C NMR (151 MHz, CD_2Cl_2) δ = 146.47, 140.12, 131.85, 129.79, 128.83, 128.48, 36.77, 36.28, 35.48, 31.88, 30.02, 29.73, 29.64, 29.55, 29.52, 29.48, 29.46, 29.39, 29.26, 29.22, 29.06, 27.12, 25.29, 22.67, 22.64, 21.35, 13.83, 13.57, 10.47. HRMS (FD, [M]$^+$) for $C_{108}H_{122}N_8Si_2S_6$ calcd. 1778.76497, found: 1778.76603.

Compound **Si-2H$^+$** was obtained as a blue solid (15 mg, 6%). ^{1}H NMR (600 MHz, $CDCl_3$) δ = 12.42 (s, 4H), 12.00 (s, 4H), 10.07 (s, 4H), 8.42 (d, J = 6.1 Hz, 4H), 8.10 (s, 8H), 3.25 (s, 12H), 2.33 (s, 24H), 2.08–1.96 (m, 12H), 1.88–1.71 (m, 20H), 1.37–1.32 (m, 13H), 0.93 (d, J = 29.6 Hz, 24H), -5.64 (s, 3H). ^{13}C NMR (101 MHz, $CDCl_3$) δ = 151.98, 142.93, 140.84, 140.64, 139.69, 139.32, 133.48, 131.32, 130.91, 129.88, 129.09, 123.94, 121.91, 120.90, 37.04, 36.63, 31.89, 29.71, 29.50, 29.43, 29.30, 29.21, 27.16, 23.16, 22.67, 22.18, 19.20, 14.28, 14.10, 10.84, 1.04. HRMS (FD, [M]$^+$) for $C_{108}H_{122}N_8Si_2S_6$ calcd. 1778.76896, found: 1778.76282.

Compound **Si2F-2H$^+$** was obtained as a blue solid (10 mg, 4%). ^{1}H NMR (600 MHz, CD_2Cl_2) δ = 11.71 (s, 4H), 11.07 (s, 4H), 9.34 (s, 5H), 7.99 (s, 10H), 7.06 (s, 3H), 3.14 (s, 14H), 2.26 (d, J = 9.1

Hz, 25H), 1.60 (s, 13H), 1.31 (s, 20H), 0.82 (d, J = 7.4 Hz, 13H), 0.59 (t, J = 7.4 Hz, 26H). ^{13}C NMR (101 MHz, CD_2Cl_2) δ = 152.50, 150.04, 139.44, 129.79, 128.86, 114.80, 113.77, 90.71, 70.48, 36.84, 36.37, 35.71, 31.87, 31.58, 29.68, 29.47, 29.28, 29.16, 27.12, 22.81, 22.65, 21.75, 21.49, 19.67, 18.78, 13.87, 13.71, 10.53. ^{19}F NMR (376 MHz, $CDCl_3$) δ = -137.58. HRMS (FD, $[M]^+$) for $C_{116}H_{128}N_4F_4Si_2S_4$ calcd. 1836.84910, found: 1836.84894.

Compound **S2F-2H$^+$** was obtained as a blue solid (20 mg, 7%). ^{1}H NMR (600 MHz, CD_2Cl_2) δ = 7.19 (t, J = 9.5 Hz, 4H), 6.92 (s, 8H), 6.31 (s, 4H), 6.10 (s, 4H), 2.33 (s, 12H), 2.17 (s, 24H). ^{13}C NMR (151 MHz, CD_2Cl_2) δ = 161.26, 155.69, 149.00, 146.47, 146.16, 145.44, 138.25, 136.59, 136.11, 134.66, 129.86 (d, J = 17.1 Hz), 128.16, 126.10, 29.65, 19.15.^{19}F NMR (376 MHz, CD_2Cl_2) δ = -137.88. HRMS (FD, $[M]^+$) for $C_{84}H_{52}F_8N_4S_6$ calcd. 1388.42599, found: 1388.27742.

Compound **S-2H$^+$** was obtained as a blue solid (22 mg, 12%). ^{1}H NMR (400 MHz, CD_2Cl_2) δ 7.40 (m, J = 6.1, 3.3, 1.0 Hz, 4H), 7.17 – 7.12 (m, 4H), 6.91 (t, J = 1.7 Hz, 8H), 6.39 – 6.36 (m, 4H), 6.09 – 6.05 (m, 4H), 2.31 (d, J = 2.5 Hz, 12H), 2.17 (d, J = 2.5 Hz, 24H). LRMS (FD, $[M]^+$) for $C_{84}H_{60}N_4S_6$ calcd. 1316.31, found: 1319.2.

Compound **SBT-2H$^+$** was obtained as a blue solid (16 mg, 9%). ^{1}H NMR (400 MHz, CD_2Cl_2) δ 13.16 (s, 4H), 12.38 (s, 4H), 8.16 (d, J = 4.9 Hz, 8H), 3.25 (s, 13H), 2.36 (s, 24H), -3.64 (s, 4H). HRMS (FD, $[M]^+$) for $C_{76}H_{54}N_8S_8$ calcd. 1334.22316, found: 1334.22375.

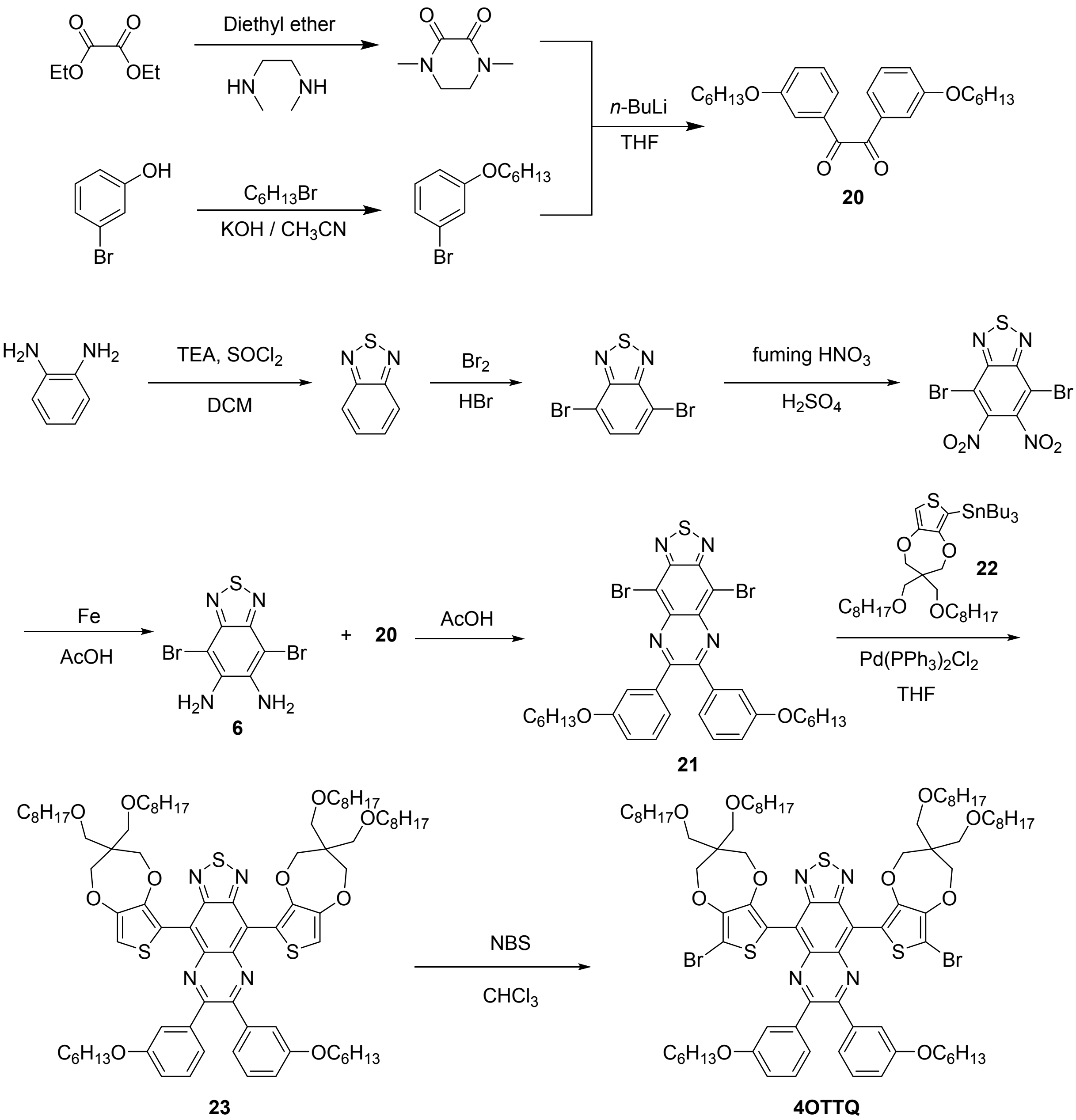

Scheme S1. Synthetic routes of the acceptor core (4OTTQ) of the conjugate polymer.

Synthesis of 3-(4,4,5,5-tetramethyl-1,3,2-dioxaborolan-2-yl)-1-(triisopropylsilyl)-1H-pyrrole, [22]. 3,3-bis((octyloxy)methyl)-3,4-dihydro-2H-thieno[3,4-b][1,4]dioxepine (250 mg, 0.57 mmol) was placed into a round-bottom flask under N_2 atmosphere with 6 mL of dry THF. The mixture was cooled to -78 ^{o}C and 0.43 mL of *n*-BuLi (1.6 M, 0.68 mmol) was added dropwise and stirred for 1 h. After that, tributyltin chloride (222 mg, 0.68 mmol) was added slowly at -78 ^{o}C and then heated to room temperature for stirring for another 8 h. After the reaction, THF was evaporated by a rotary evaporator and then CH_2Cl_2 was added to extract with brine three times. The crude product is roughly purified using flash column chromatography and immediately used for the next step to afford 332 mg (80%) of compound **22** as yellow liquid. ^{1}H NMR (600 MHz, $CDCl_3$) δ 6.44 (s, 1H),

4.05 – 3.87 (m, 5H), 3.46 (d, J = 3.5 Hz, 5H), 3.28 (d, J = 5.8 Hz, 5H), 1.65 – 1.43 (m, 6H), 1.36 – 1.23 (m, 25H), 1.06 (d, J = 8.1 Hz, 2H), 0.88 (dt, J = 17.6, 7.2 Hz, 21H). ^{13}C NMR (101 MHz, $CDCl_3$) δ 149.71, 111.33, 104.83, 70.00, 69.84, 47.78, 39.64, 30.70, 29.14, 29.00, 27.86, 27.21, 24.04, 23.10, 13.67, 11.13, 10.56. HRMS (FD, $[M]^+$) for $C_{37}H_{70}O_4SSn$ calcd. 730.40113, found: 730.40097.

Synthesis of 3-(4,4,5,5-tetramethyl-1,3,2-dioxaborolan-2-yl)-1-(triisopropylsilyl)-1H-pyrrole, [23]. Compound **21** (100 mg, 0.14 mmol), compound **22** (313 mg, 0.43 mmol), and $Pd(PPh_3)Cl_2$ (44 mg, 0.06 mmol) were added into a flask under nitrogen atmosphere. To this mixture, 10 mL of dry toluene was added. The resulting mixture was then heated at 100 °C for 48 h. After cooling to room temperature, the solvent was removed and the product was added with CH_2Cl_2 to extract with brine for three time. Afterwards, the organic solvent was removed under reduced pressure and the product was purified by column chromatography on silica gel with hexane/CH_2Cl_2 (2:1, v/v) as eluent afford 89 mg (45%) of compound **23** as a deep-red solid. ^{1}H NMR (600 MHz, $CDCl_3$) δ = 7.38 – 7.34 (m, 2H), 7.24 – 7.18 (m, 4H), 6.93 (m, *J* = 6.5, 2.6 Hz, 2H), 6.86 (s, 2H), 4.13 (d, *J* = 29.9 Hz, 8H), 3.88 (t, *J* = 6.6 Hz, 4H), 3.44 (qd, *J* = 8.8, 4.6 Hz, 8H), 3.25 (d, *J* = 5.8 Hz, 8H), 1.77 – 1.68 (m, 6H), 1.59 – 1.15 (m, 54H), 0.88 – 0.85 (m, 19H). ^{13}C NMR (101 MHz, $CDCl_3$) δ = 158.87, 153.27, 152.78, 149.71, 148.82, 139.68, 136.98, 128.94, 122.67, 122.06, 116.82, 115.49, 114.75, 108.02, 73.70, 73.59, 71.67, 69.24, 68.09. HRMS (FD, $[M]^+$) for $C_{108}H_{122}N_8Si_2S_6$ calcd. 1416.81611, found: 1416.81621.

*Synthesis of 4,9-bis(8-bromo-3,3-bis((octyloxy)methyl)-3,4-dihydro-2H-thieno[3,4-b][1,4]dioxepin-6-yl)-6,7-bis(3-(hexyloxy)phenyl)-[1,2,5]thiadiazolo[3,4-g]quinoxaline (**4OTTQ**).* In a single-neck flask was added compound **23** (138 mg, 0.1 mmol) and 3 mL of $CHCl_3$ and then wrapped in aluminum foil. *N*-bromosuccinimide (40 mg, 0.22 mmol) in $CHCl_3$ was then added slowing via a dropping funnel and stirred at room temperature in the dark for 8 h. After the reaction, the mixture was extracted with CH_2Cl_2 and $Na_2S_2O_3$. Afterwards, the organic solvent was removed under reduced pressure and the product was purified by column chromatography on silica gel with hexane/CH_2Cl_2 (1:1, v/v) as eluent afford 126 mg (80%) of compound **4OTTQ** as a deep-red solid. were added in a flask under nitrogen atmosphere. To this mixture, 10 mL of dry DMF was added. The resulting mixture was then heated at 130 °C for 12 h. After cooling to room temperature, the product was added with CH_2Cl_2 to extract with brine for three time. Afterwards, the organic solvent was removed under reduced pressure and the product was purified by column chromatography on silica gel with hexane/CH_2Cl_2 (1:1, v/v) as eluent. ^{1}H NMR (600 MHz, $CDCl_3$) δ = 7.47 (t, *J* = 2.1 Hz, 1H), 7.18 (t, *J* = 7.9 Hz, 1H), 7.11 (m, *J* = 7.7, 1.3 Hz, 1H), 6.94 (ddd, *J* = 8.3, 2.6, 1.0 Hz, 1H),

4.17 (d, J = 47.6 Hz, 4H), 3.95 (t, J = 6.4 Hz, 2H), 3.45 (s, 4H), 3.24 (m, J = 5.9, 1.5 Hz, 4H), 1.80 – 1.72 (m, 2H), 1.45 (m, J = 10.2, 4.4 Hz, 5H), 1.35 – 1.21 (m, 24H), 0.85 (m, J = 5.7, 3.0 Hz, 9H). ^{13}C NMR (101 MHz, $CDCl_3$) δ = 159.14, 153.47, 148.02, 147.86, 139.38, 136.72, 128.92, 122.73, 121.11, 115.00, 114.28, 97.21, 71.68, 69.21, 68.17, 47.94, 31.82, 31.59, 29.51, 29.41, 29.28, 29.20, 26.12, 25.79, 22.63, 14.07, 14.05. HRMS (FD, $[M]^+$) for $C_{108}H_{122}N_8Si_2S_6$ calcd. 1572.63713, found: 1572.63686.

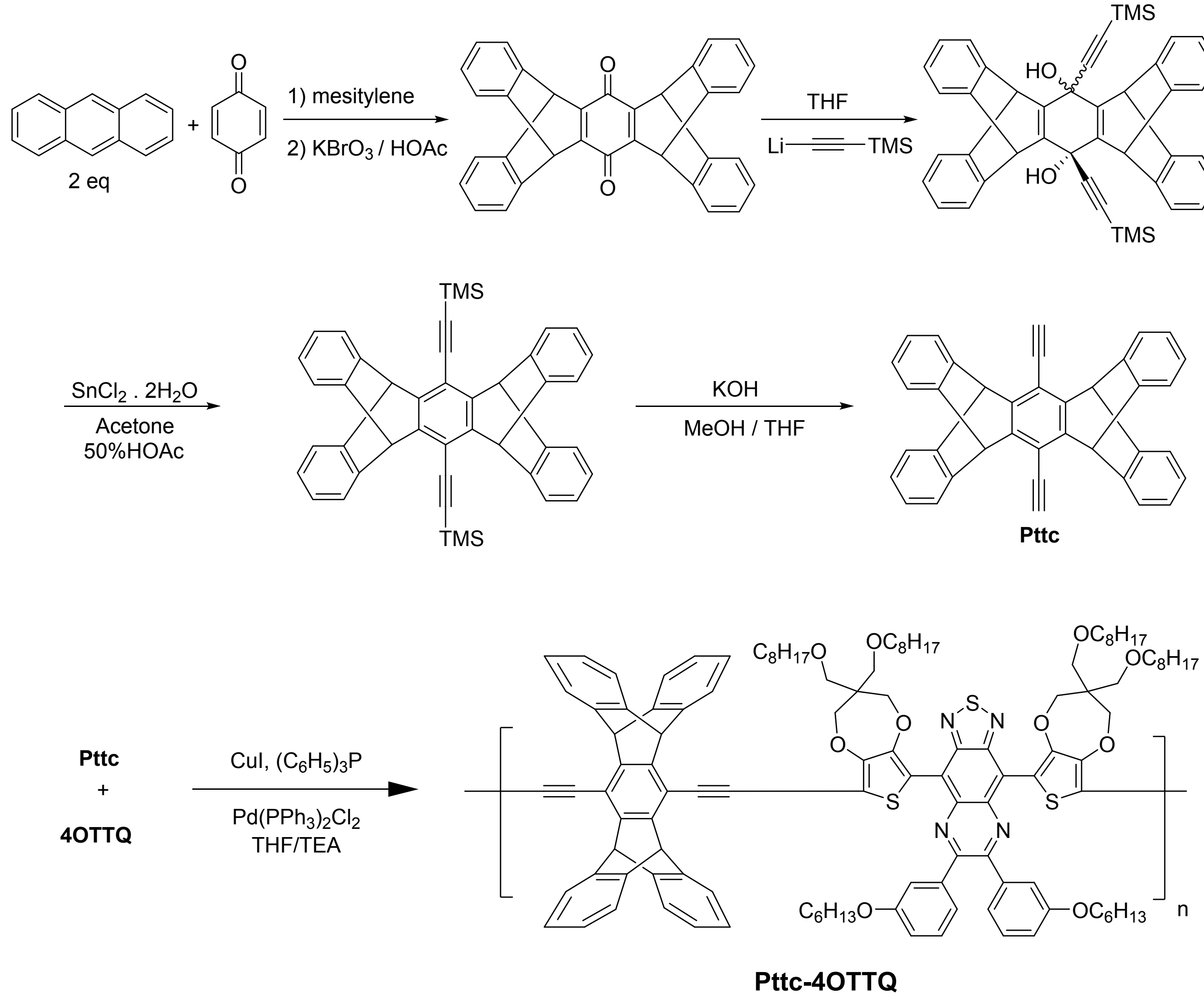

Scheme S2. Synthetic routes for the synthesis of Pttc-4OTTQ polymer.

*Synthesis of **Pttc-4OTTQ conjugated polymer**.* For the synthesis of conjugated polymers, we employed Sonogashira polycondensation reaction as displayed in Scheme S2. Briefly, **Pttc** (16 mg, 0.03 mmol), compound **4OTTQ** (52 mg, 0.03 mmol), CuI (0.6 mg, 0.003 mmol), PPh_3 (2 mg, 0.008 mmol), 0.5 mL of triethylamine, and $Pd(PPh_3)_4$ (1.2 mg, 0.001 mmol) were used in the polymerization crossing-coupling in mixture of dry THF/toluene (3:1, v/v) for 72 h at 95 °C. After the reaction, phenylacetylene (50 μL) was injected into the mixture and stirred for 1 h, followed by

the addition of bromobenzene (50 μL). After the polymerization, the products were purified by reprecipitation in cold methanol/acetone and washed with copious cold acetone/methanol. The products were dissolved in CH_2Cl_2 to extract with brine for three times to obtain 30-40 mg of polymer as a deep-green solid. Pttc-4OTTQ: M_n = 4434, M_w = 8384, PDI = 1.89.

Preparation of Pdots. Typically, 90 μL of semiconducting polymer Pttc-4OTTQ solution (1 mg/mL in THF), 3.0 mg of mPEG-DSPE-2000 (Laysan Bio. Inc.), and 20 μL of octaphyrin dyes were mixed well in 0.4 mL THF. The THF solutions containing polymers were injected into 4 mL H_2O under vigorous sonication. After that, THF was removed under reduced pressure at room temperature. The resulting Pdot solution was passed through a 0.2 μm cellulose acetate syringe filter and were ready for use. The as-prepared Pdot solution was optically stable for at least two weeks at room temperature in the dark. For in vivo imaging, the Pdot solution was concentrated with a centrifugal filter (100 kDa, MWCO) to have the final concentration of 2-9 mg/mL.

Characterization of Pdots. The average particle size was determined by dynamic light scattering and transmission electron microscopy (TEM). Dynamic light scattering measurements were performed by a Beckman Coulter N5 submicron particle size analyzer. TEM images of the synthesized Pdots were acquired using a Hitachi HT7700 transmission electron microscope at an acceleration voltage of 100 kV. For TEM, a drop of Pdot aqueous solution was placed onto a carbon-coated grid and allowed to evaporate at room temperature. The absorption spectra of Pdots were measured using UV-visible spectroscopy (Dynamica Halo DB20S, Dynamica Scientific, for 400-1100 nm) and BWTek Sol 1.7 (Sensors Unlimited, Inc., for 900-1700 nm). The fluorescence spectra were collected using a FS5 spectrofluorometer (Edinburgh Instruments Ltd, UK) under 985 or 1064 nm laser excitation.

The fluorescence quantum yields (QY) of the polymers or monomers were determined from the relative fluorescence quantum yields by comparing with the IR-1061 dye (QY = 0.59 % in CH_2Cl_2) as the reference.

The equation used was as follows:

$$QY_s = QY_r \times (K_s/K_r) \times (n_s/n_r)^2$$

Where subscripts s and r denote sample and reference, respectively. K is the slope of the integrated fluorescence intensity against the absorbance plot (linear fitting for at least five points). n is the refractive index of the solvent. The maximal absorptions of all samples were carefully controlled to lower than 0.1 for all measurements to eliminate self-quenching or re-absorption/re-emission issues.

The optical parameters used for the measurements were as follows: octaphyrins derivatives vs. IR-1061 (λ_{ex} = 925 nm, λ_{em} =1050-1400 nm).

Computational Method. All geometry optimization and frequency analyses of **Si-2H$^+$**, **Si2F-2H$^+$**, **SiBT-2H$^+$** and **S2F-2H$^+$**, both in the ground state and the first excited states using DFT at the B3LYP-D3/6-31G* level, and *n*-alkyl groups for **Si-2H$^+$**, **Si2F-2H$^+$**, **SiBT-2H$^+$**, and **O4TTQ** are replaced by methyl or ethyl groups for better computational efficiency. The structure optimization calculation and frequency analysis of the **dye・4OTTQ** complex were also completed in B3LYP-D3/6-31G*. Theoretical absorption optical properties have been calculated on ground state geometries using TD-PBE38/6-31G* with CH_2Cl_2 as solvent using the conductor-like polarizable continuum model (CPCM). Pentyl acetate is used to model with **Pdot** condition which is the aliphatic part of mPEG-DSPE. All the DFT calculations were carried out employing Gaussian 16 Revision A.03. (Gaussian 16, Revision A.03, Frisch, M. J.; Trucks, G. W.; Schlegel, H. B.; Scuseria, G. E.; Robb, M. A.; Cheeseman, J. R.; Scalmani, G.; Barone, V.; Petersson, G. A.; Nakatsuji, H.; Li, X.; Caricato, M.; Marenich, A. V.; Bloino, J.; Janesko, B. G.; Gomperts, R.; Mennucci, B.; Hratchian, H. P.; Ortiz, J. V.; Izmaylov, A. F.; Sonnenberg, J. L.; Williams-Young, D.; Ding, F.; Lipparini, F.; Egidi, F.; Goings, J.; Peng, B.; Petrone, A.; Henderson, T.; Ranasinghe, D.; Zakrzewski, V. G.; Gao, J.; Rega, N.; Zheng, G.; Liang, W.; Hada, M.; Ehara, M.; Toyota, K.; Fukuda, R.; Hasegawa, J.; Ishida, M.; Nakajima, T.; Honda, Y.; Kitao, O.; Nakai, H.; Vreven, T.; Throssell, K.; Montgomery, J. A., Jr.; Peralta, J. E.; Ogliaro, F.; Bearpark, M. J.; Heyd, J. J.; Brothers, E. N.; Kudin, K. N.; Staroverov, V. N.; Keith, T. A.; Kobayashi, R.; Normand, J.; Raghavachari, K.; Rendell, A. P.; Burant, J. C.; Iyengar, S. S.; Tomasi, J.; Cossi, M.; Millam, J. M.; Klene, M.; Adamo, C.; Cammi, R.; Ochterski, J. W.; Martin, R. L.; Morokuma, K.; Farkas, O.; Foresman, J. B.; Fox, D. J. Gaussian, Inc., Wallingford CT, 2016.)

MTT Assay. The cellular cytotoxicity of the Pdots was examined on HeLa cells. The number of viable cells was determined using the MTT assay with 3-(4,5-dimethylthiazole-2-yl)-2,5-phenyltetrazolium bromide. HeLa cells were first seeded in each well of a 24-well culture plate and then incubated with various concentrations of Pdots (5 μg/mL, 10 μg/mL, and 20 μg/mL) for 6 h, 12 h, and 24 h. After that, 20 μL (5 mg/mL) of MTT aqueous solution was added to each well and the cells were further incubated for 4 h at 37 °C to deoxidize MTT. The medium was then washed out and 300 μL of DMSO was added into each well to dissolve formazam crystals. Absorbance was

measured by a BioTek ELx800 microplate reader at 570 nm, while the cells cultured with the pure medium (e.g., without Pdots) served as controls.

In Vivo Fluorescence Blood Vasculature Imaging in Mice with Pdots. All animal experiments were conducted in compliance with protocols authorized by the Institutional Animal Care and Use Committee (IACUC #1100509) at NYCU. Female nude mice (BALB/cAnN.Cg-Foxn1nu/CrlNarl), aged five weeks, were acquired from the National Laboratory Animal Center and maintained in a pathogen-free environment at 24 °C with a standardized 12-h light/dark cycle. Prior to imaging, mice (n = 5 per group) were anesthetized via a rodent ventilator system delivering 2% isoflurane in air. Each subject received an intravenous tail vein injection of 100-200 μL of Pdots (5.5 mg/mL). In vivo fluorescence was tracked over time using a custom NIR-II imaging system equipped with a 1064 nm excitation laser and an InGaAs camera (Ninox 640 SU, Raptor Photonics). Emission signals were collected through a long-pass filter (1200-1400 nm; Thorlabs). Imaging parameters included a camera temperature of -80 °C, a 10 MHz analog-to-digital conversion rate, high gain settings, and exposure times of 500-1500 ms. Laser power density was maintained between 20-100 mW cm^{-2} during acquisitions.

AI-Asisted Imaging Processing. All deep-learning models were trained on an NVIDIA RTX 3090 GPU using PyTorch 1.10. The entire pipeline, including preprocessing, deep-learning transformation, vessel enhancement, and evaluation, was executed on a Windows 11 computing system with 64G ram. To enhance the contrast and structural clarity of in vivo NIR-II fluorescence imaging, this study applied deep-learning-based image processing, integrating a CycleGAN-based image-to-image translation model with Frangi vesselness filtering. This approach was adapted from previously established deep-learning methodologies for biomedical imaging[8,9] and optimized for improved vascular visualization in NIR-II fluorescence imaging. The initial fluorescence images, as shown in the first row labeled "origin," were acquired under different long-pass filter (LPF) conditions. These images exhibited significant light scattering and low contrast, limiting their ability to resolve fine vascular structures. To address these issues, a CycleGAN model was employed, building upon prior research in deep-learning-based image enhancement. The model was trained by Pytorch (epoch~250) on a dataset consisting of NIR-IIa (900-1300 nm) and NIR-IIb (1500-1700 nm) fluorescence images, allowing it to learn the transformation of low-contrast images into high-fidelity representations resembling those obtained in longer-wavelength NIR-IIb imaging. CycleGAN, originally developed for unpaired image-to-image translation,[10] was optimized in this study by fine-

tuning its loss functions to preserve vascular structures while suppressing background artifacts. The model was implemented with a U-Net-based generator for improved spatial feature retention and a PatchGAN-based discriminator, focusing on local structure refinement. This allowed the generation of enhanced images that exhibited better contrast and reduced scattering artifacts, as shown in the second row labeled "CycleGAN." Following the deep-learning transformation, a Frangi vesselness filter was applied to further enhance vascular features. This filtering method, widely used for tubular structure enhancement in biomedical imaging,[11] was adapted for NIR-II imaging to improve vessel contrast and suppress non-vascular structures. The filter was implemented in Python (OpenCV, scikit-image) and operated at multiple scales to improve vessel contrast while suppressing non-vascular structures. The filter was applied at multiple scales to capture both fine and large vessel structures while minimizing background noise.

^{1}H NMR of **compound 1**

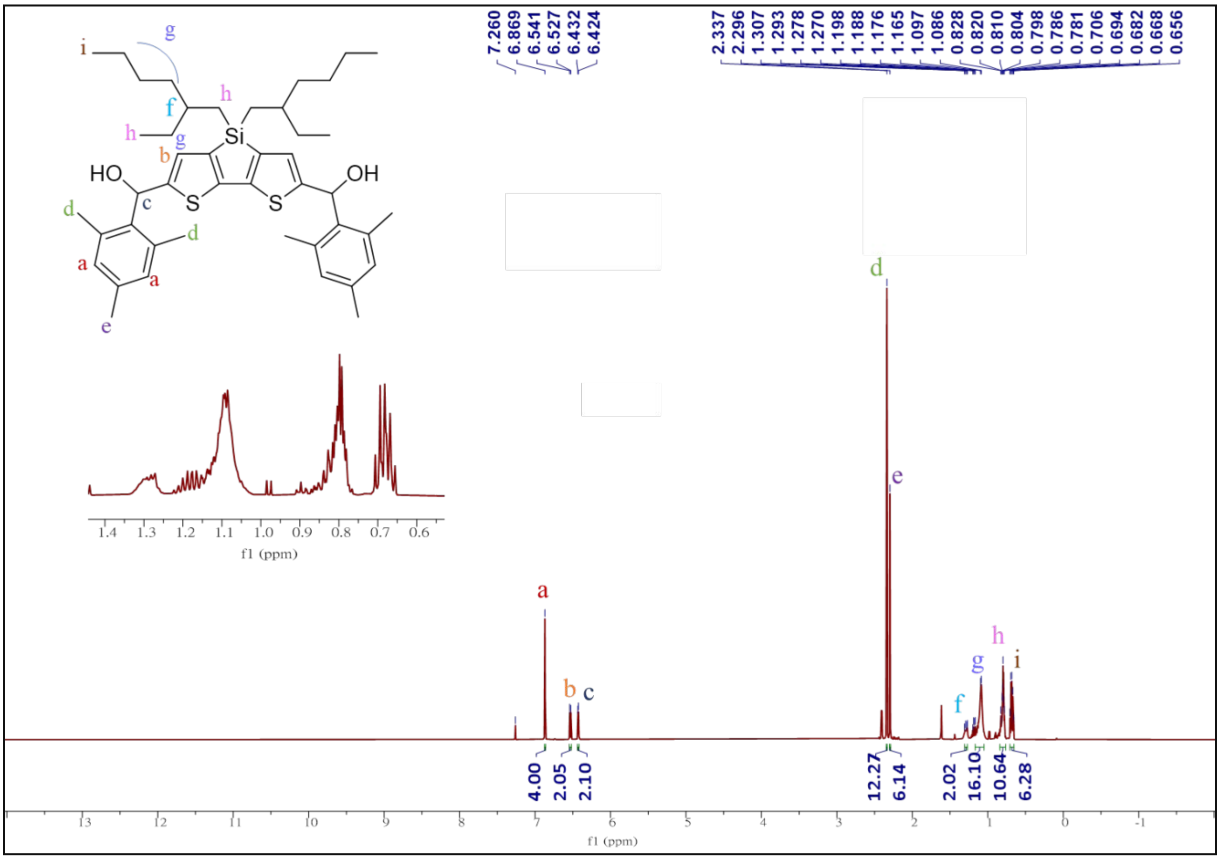

^{13}C NMR of **compound 1**

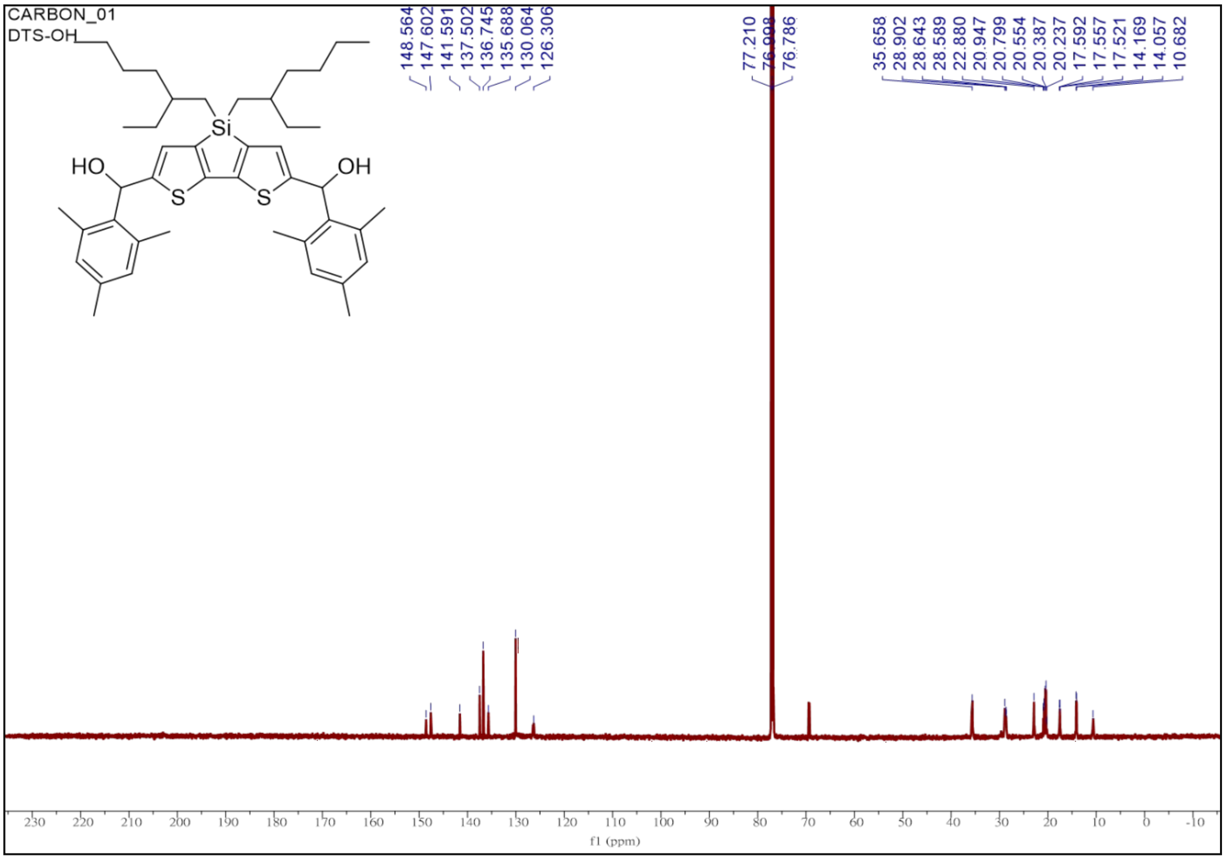

HR-Mass of **compound 1**

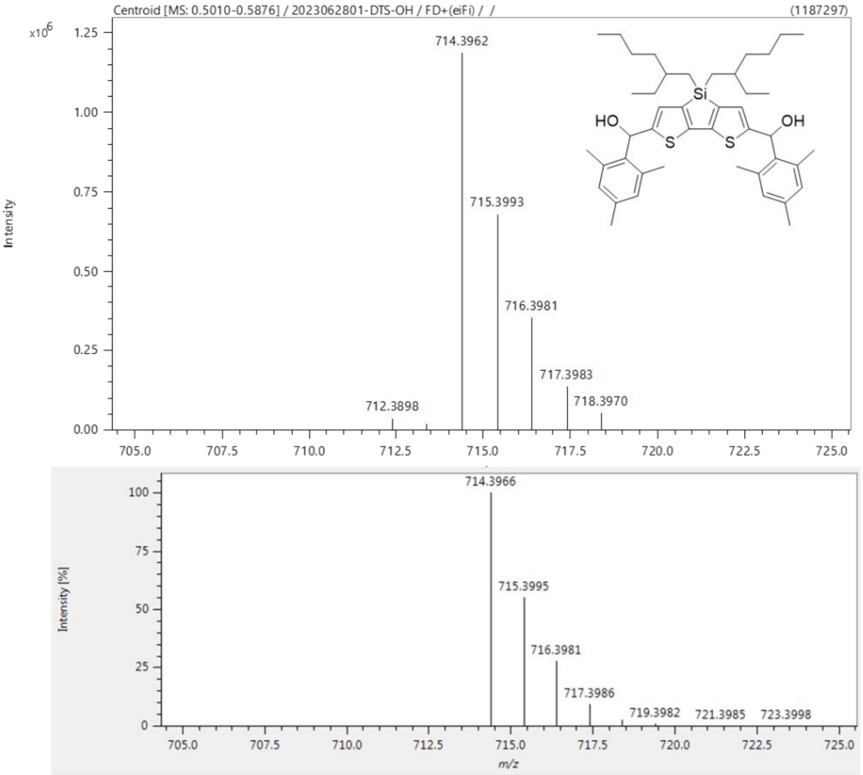

	Mass	Intensity	Formula	Calculated Mass	Mass Difference [mDa]	Mass Difference [ppm]	DBE
▸	714.39624	1187297.41	C44 H62 O2 Si S2	714.39660	-0.36	-0.50	15.0

^{1}H NMR of **compound 7**

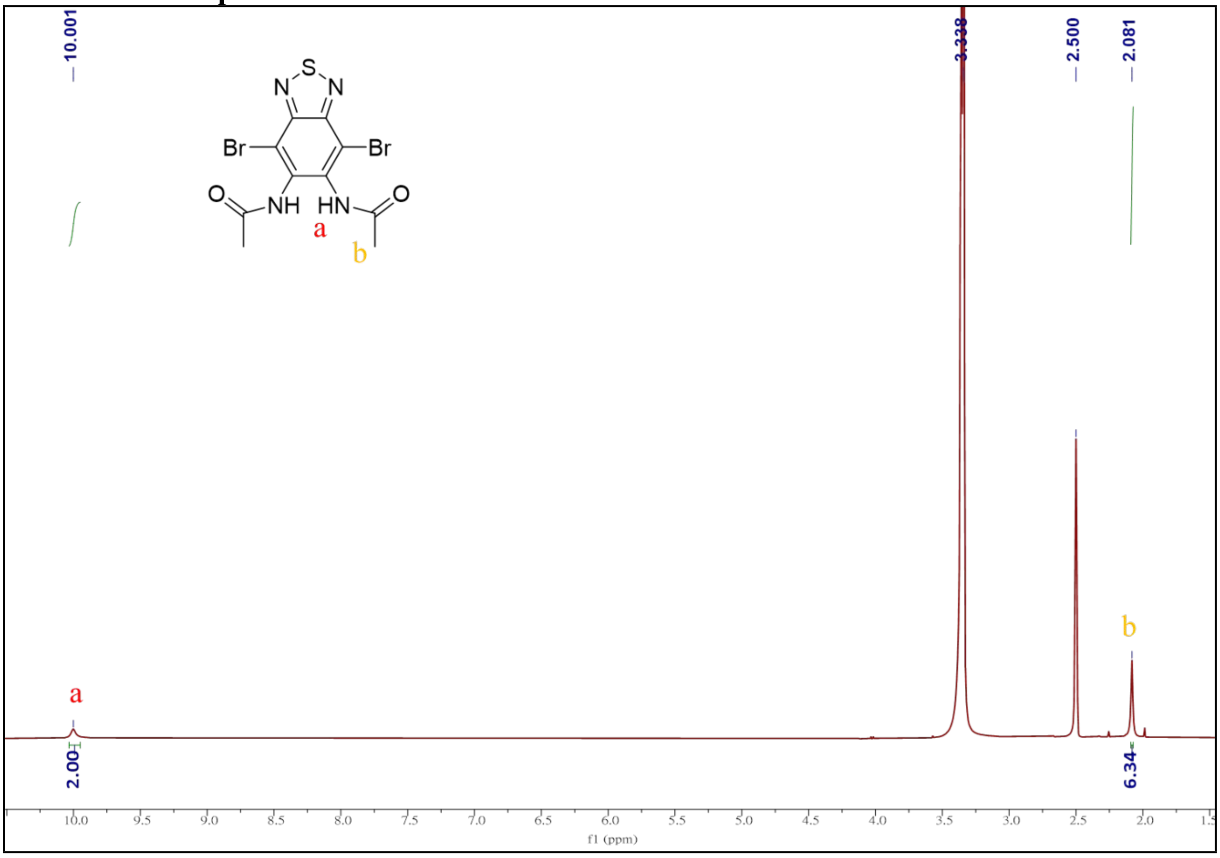

^{13}C NMR of **compound 7**

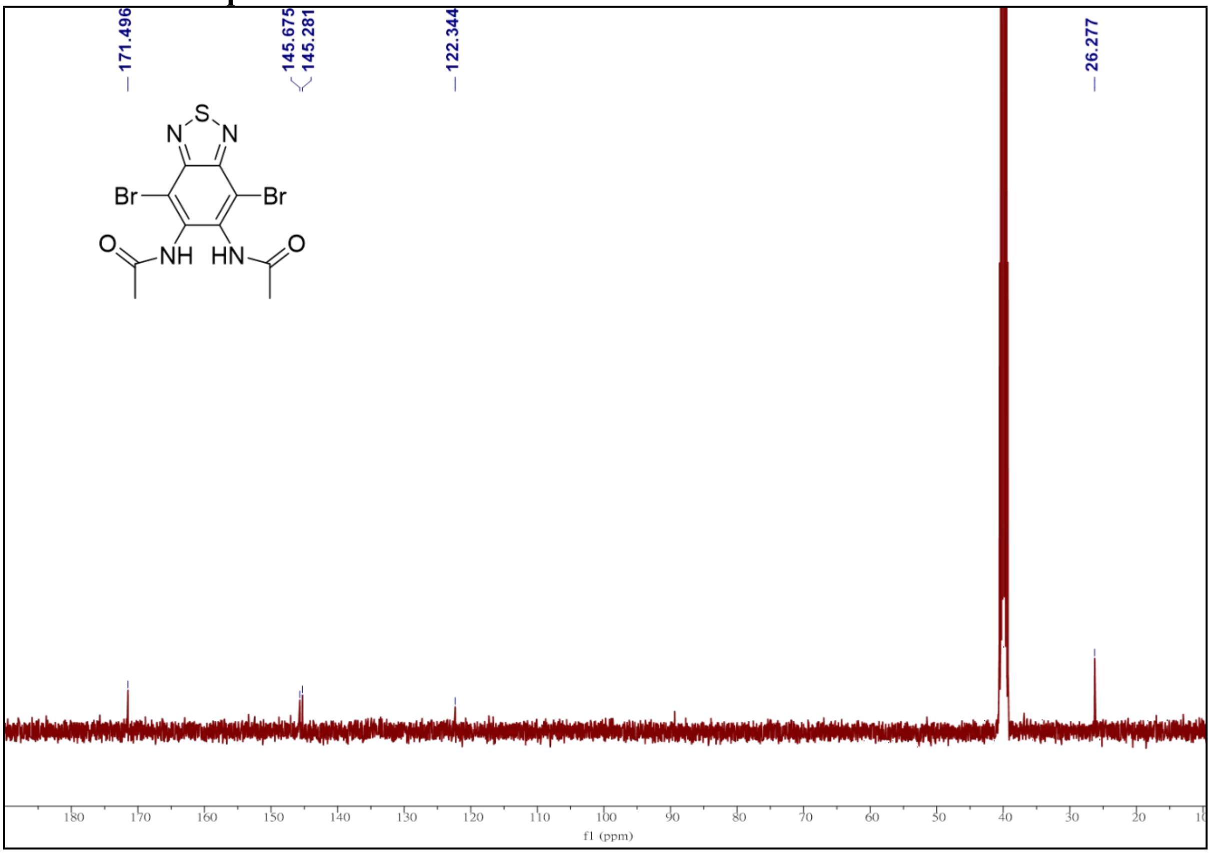

HR-Mass of **compound 7**

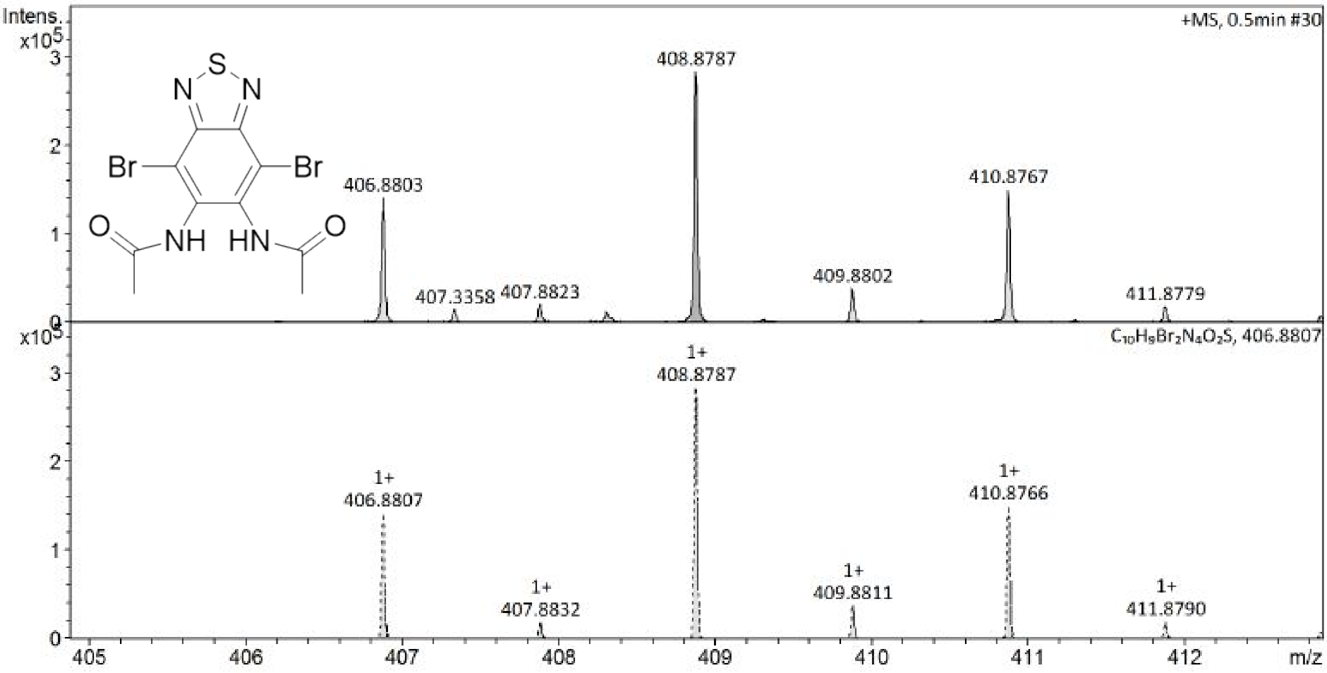

^{1}H NMR of **compound 8**

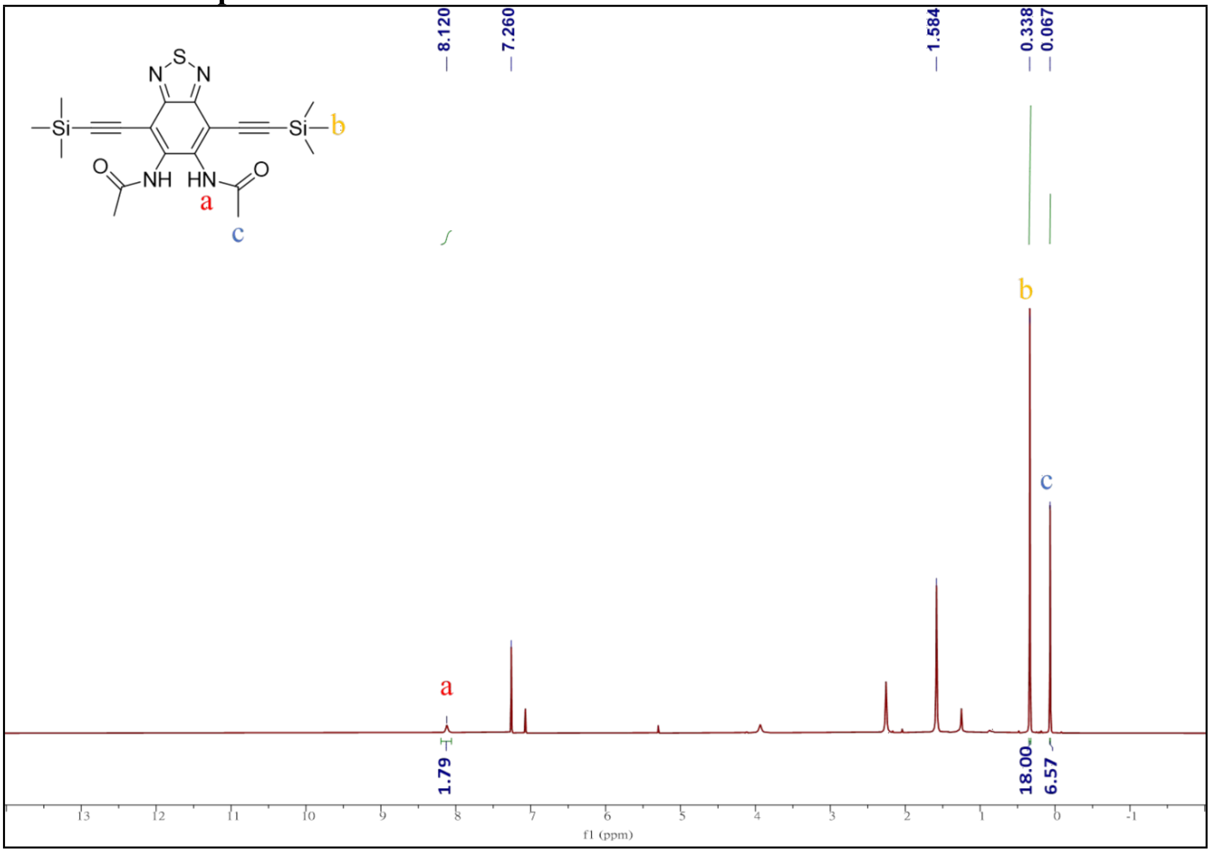

^{13}C NMR of **compound 8**

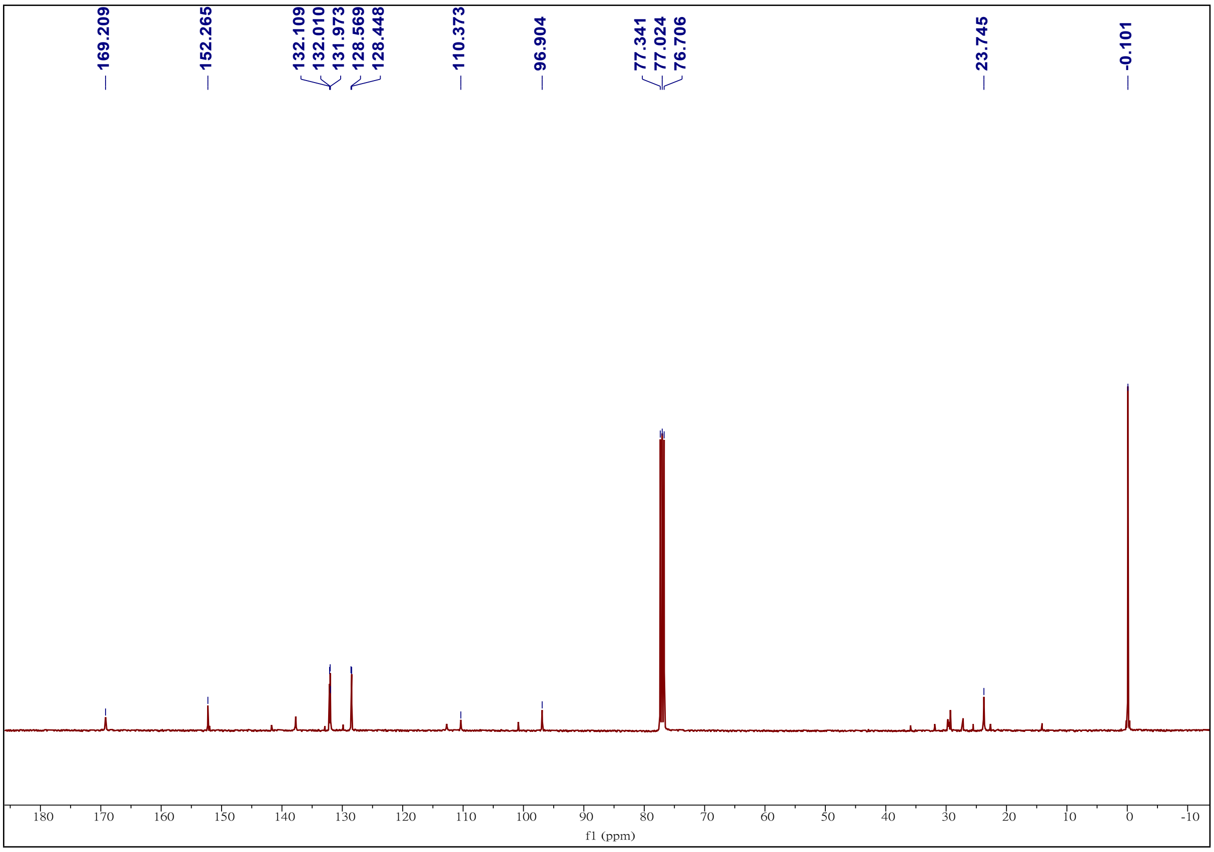

HR-Mass of **compound 8**

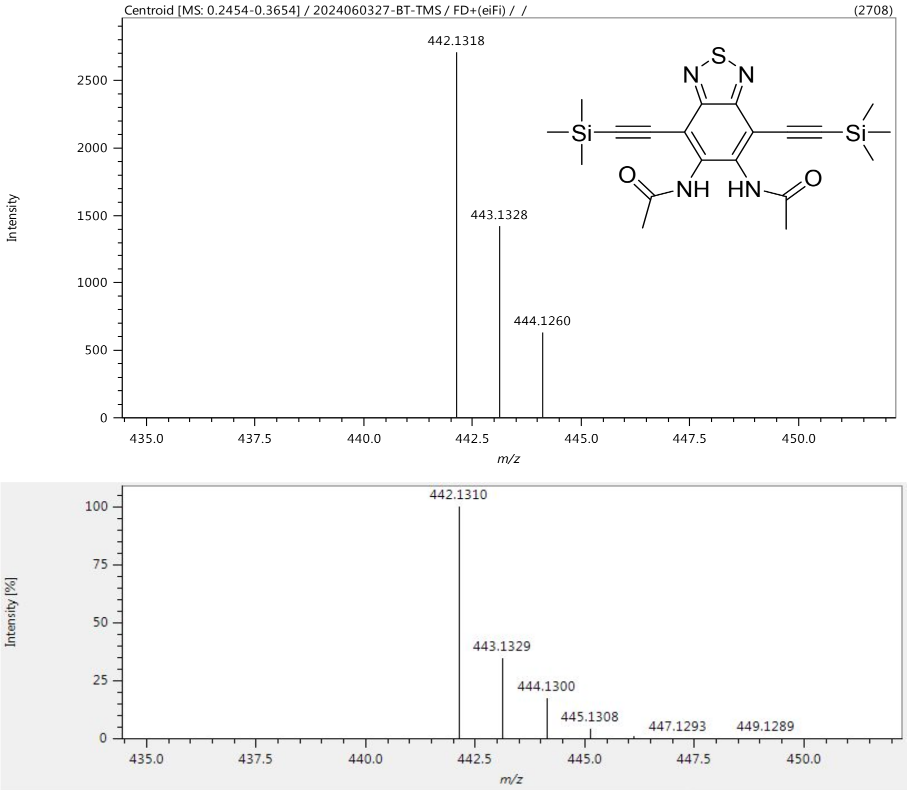

	Mass	Intensity	Formula	Calculated Mass	Mass Difference [mDa]	Mass Difference [ppm]	DBE
▶	442.13176	2708.20	C20 H26 N4 O2 Si2 S	442.13095	0.81	1.84	12.0

^{1}H NMR of **compound 9**

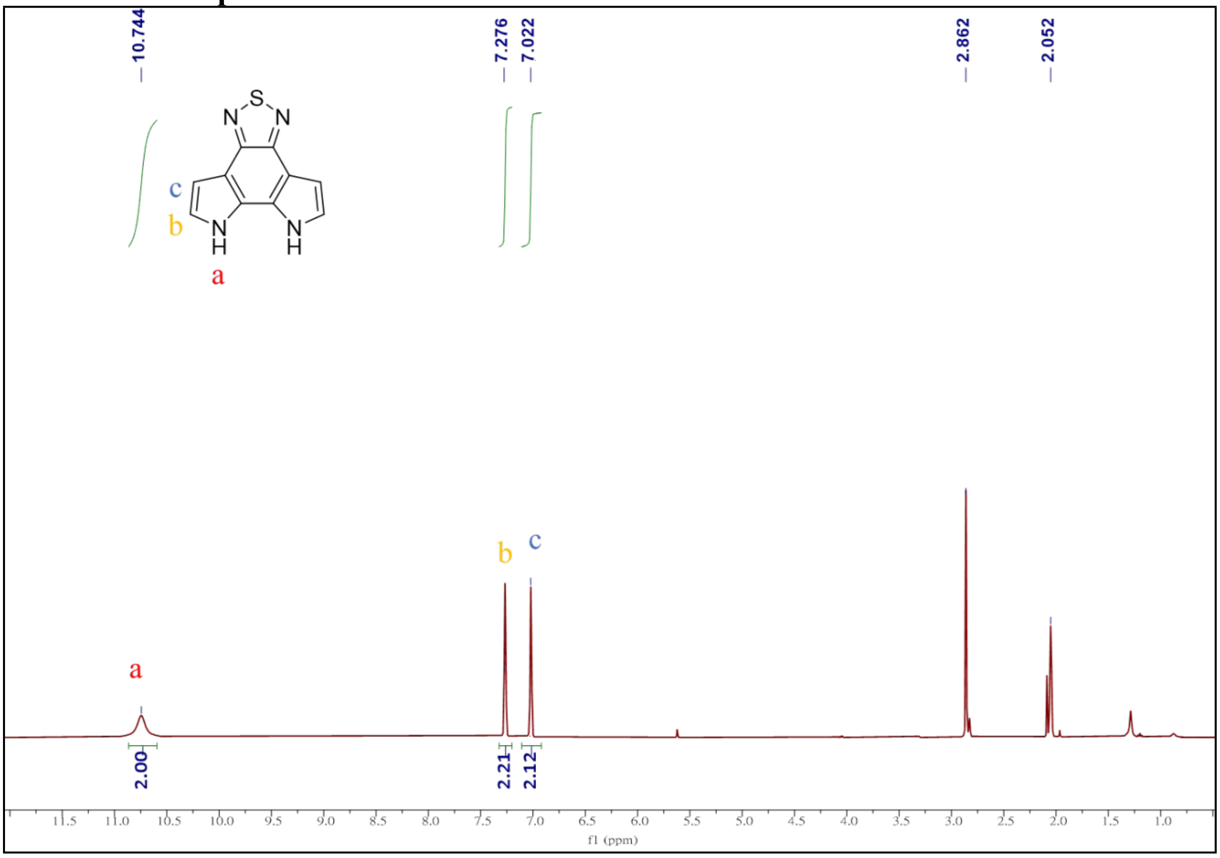

^{13}C NMR of **compound 9**

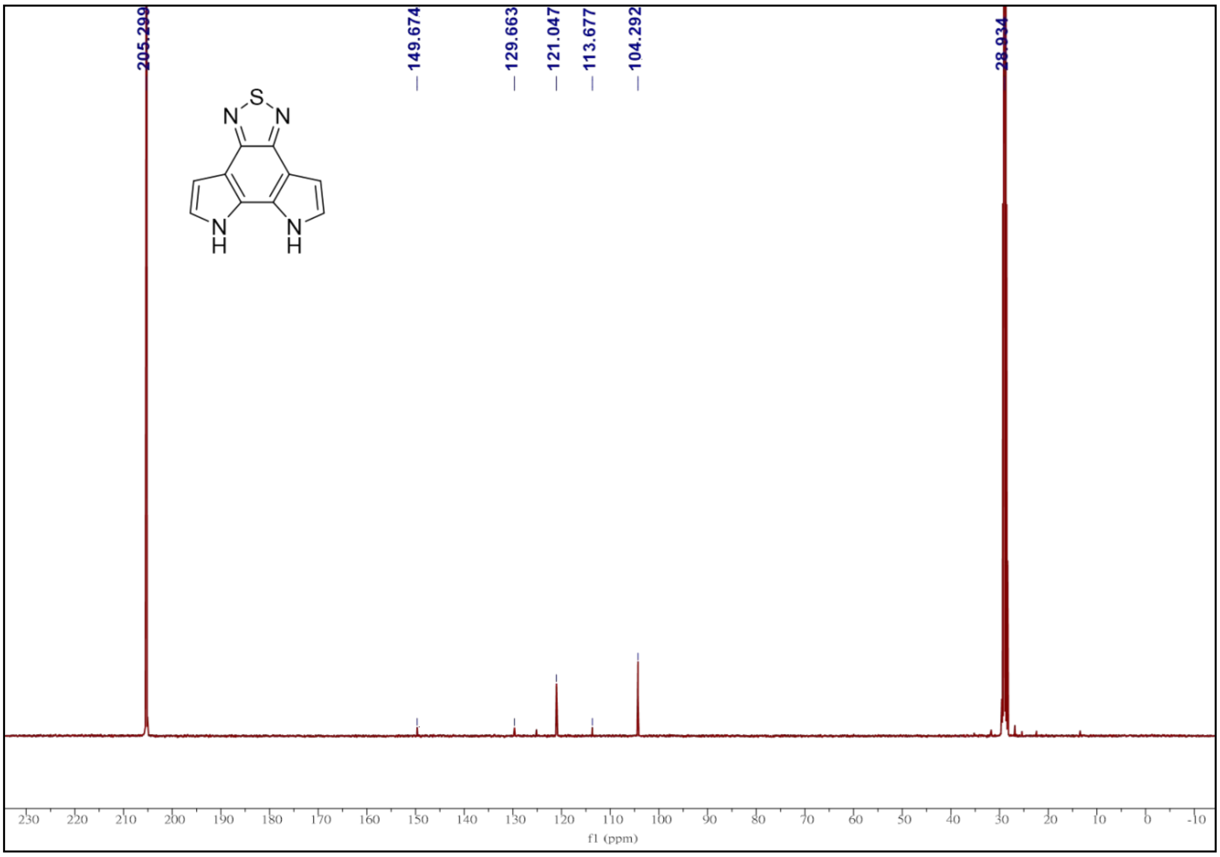

HR-Mass of **compound 9**

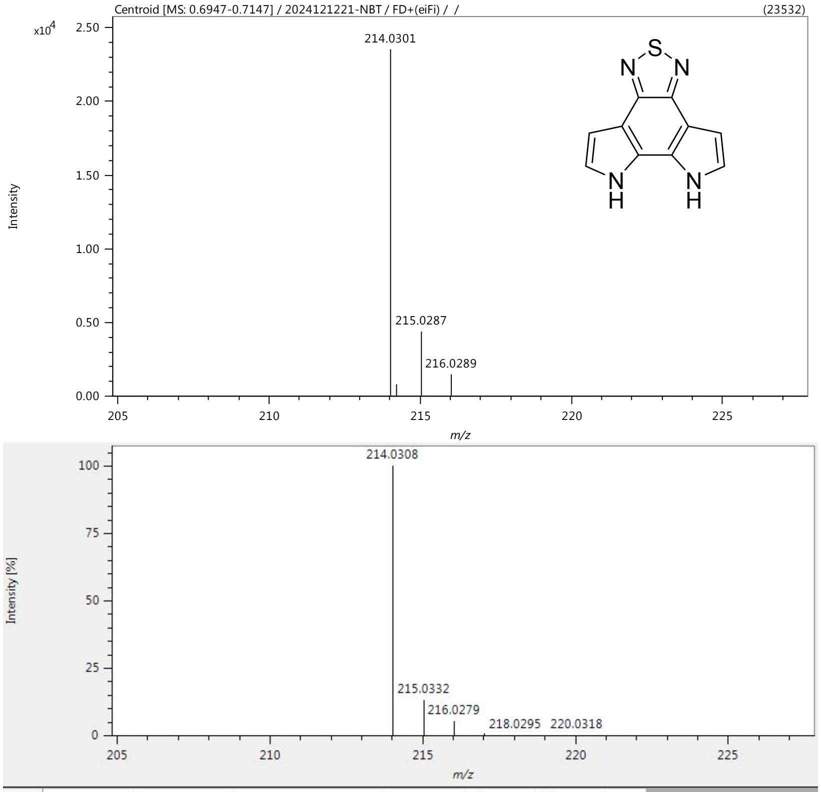

Mass	Intensity	Formula	Calculated Mass	Mass Difference [mDa]	Mass Difference [ppm]	DBE
214.03011	23531.60	C10 H6 N4 S	214.03077	-0.66	-3.07	10.0

^{1}H NMR of **compound 14**

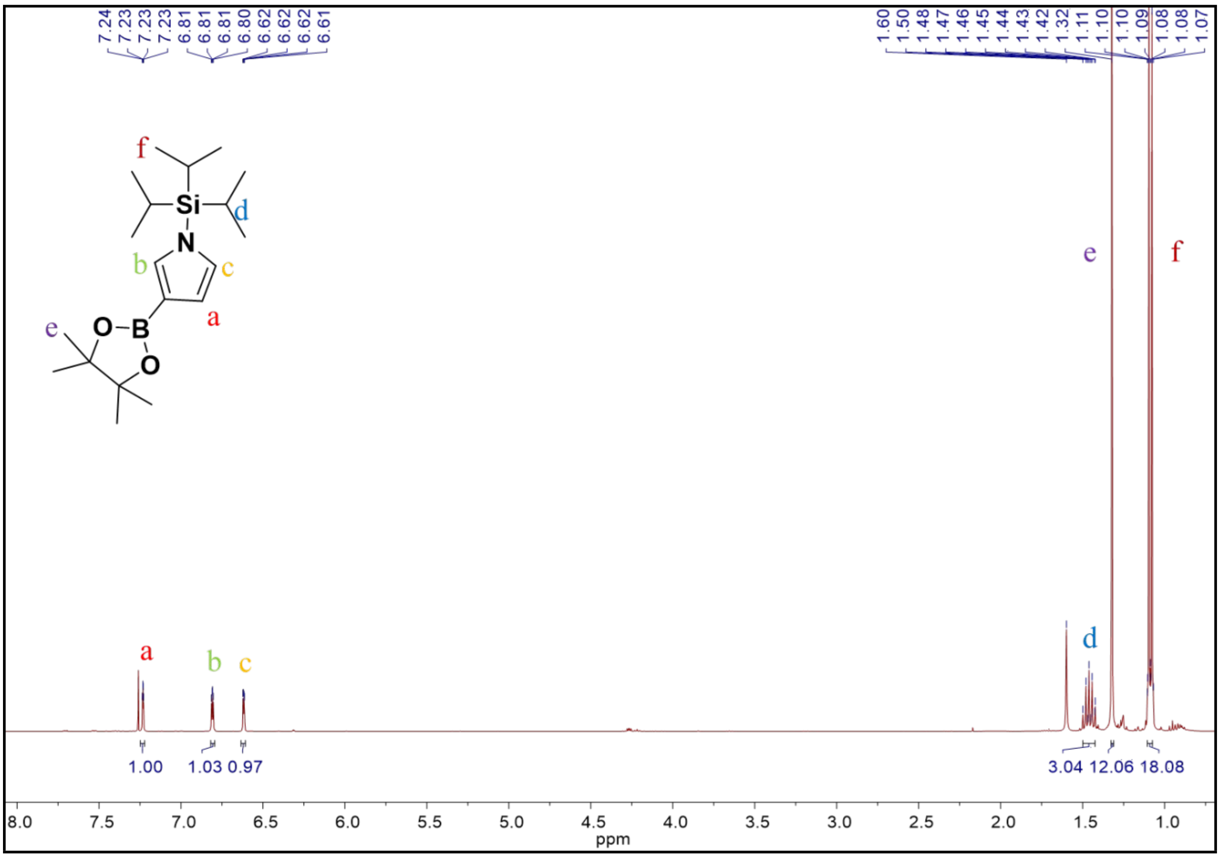

^{1}H NMR of **compound SiBT-2H**

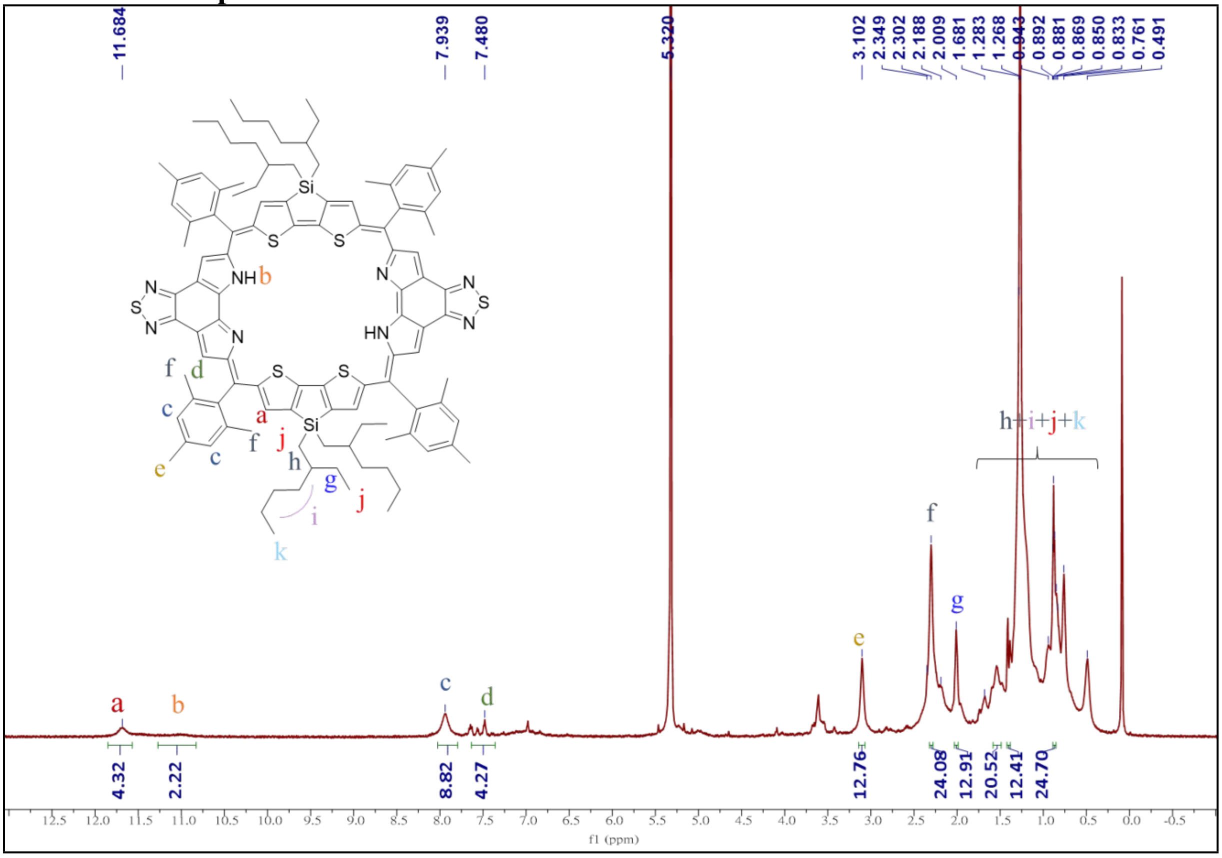

^{13}C NMR of **compound SiBT-2H**

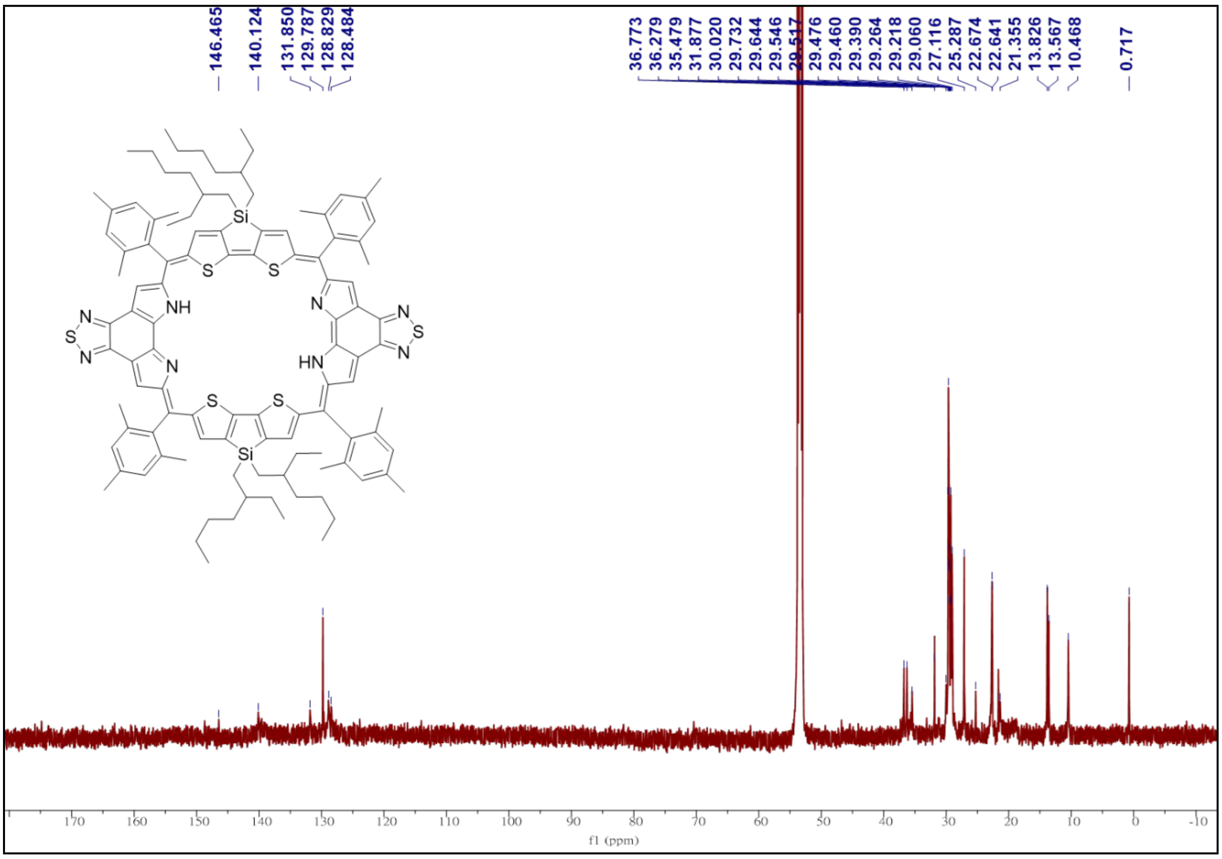

HR-Mass of **compound SiBT-2H**

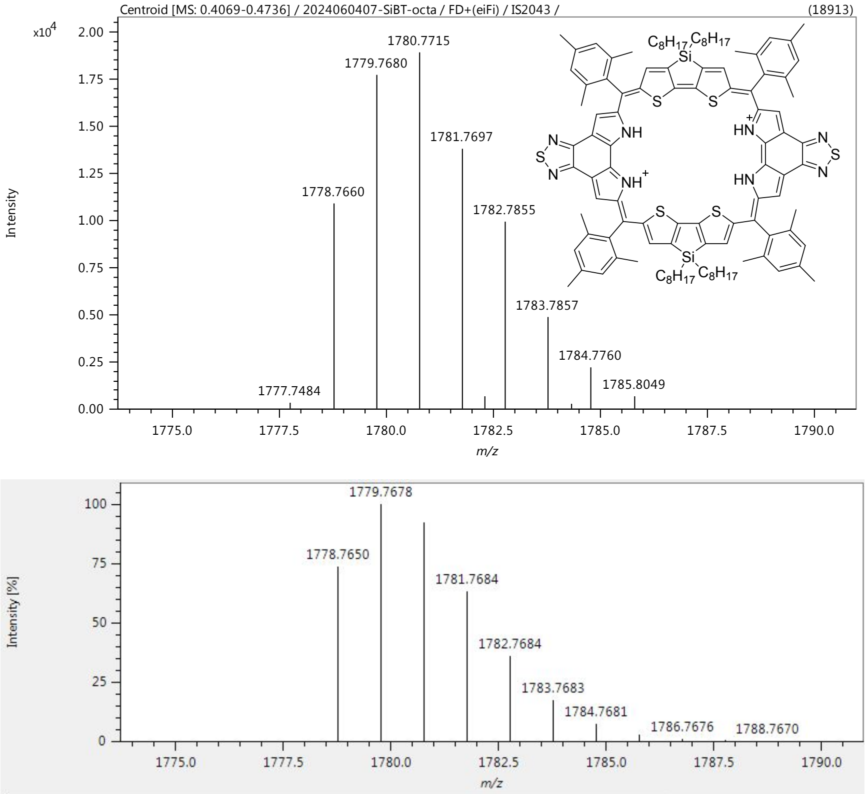

	Mass	Intensity	Formula	Calculated Mass	Mass Difference [mDa]	Mass Difference [ppm]	DBE
▸	1778.76603	10879.38	C108 H122 N8 Si2 S6	1778.76497	1.05	0.59	54.0

^{1}H NMR of **compound Si-2H**

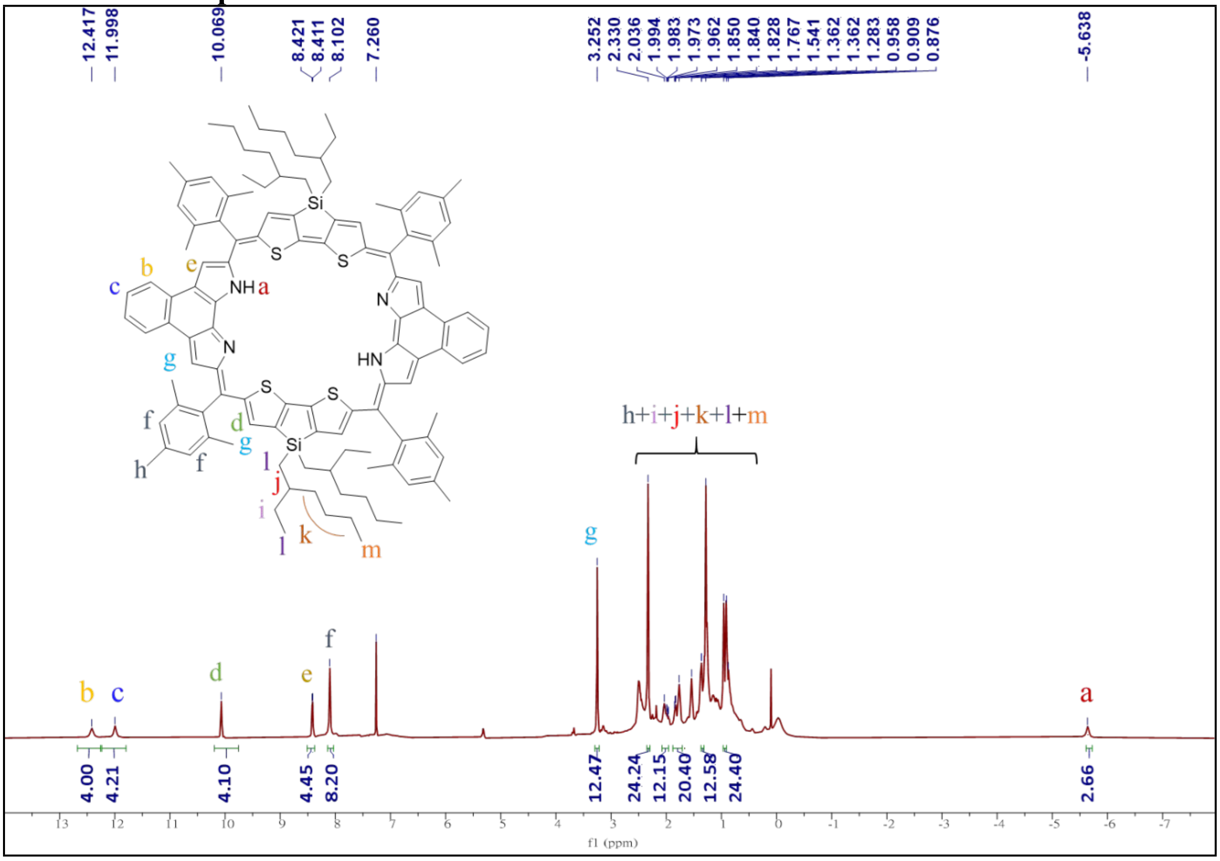

^{13}C NMR of **compound Si-2H**

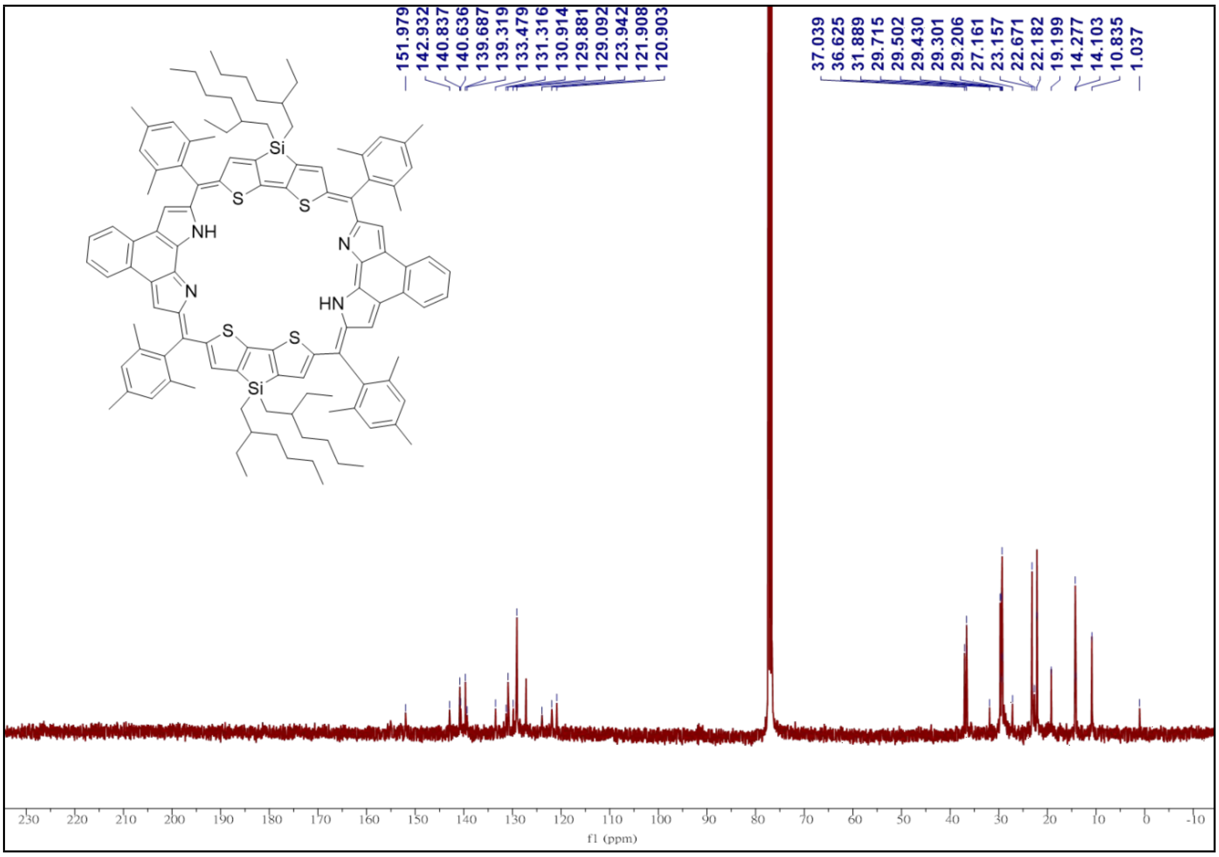

HR-Mass of **compound Si-2H**

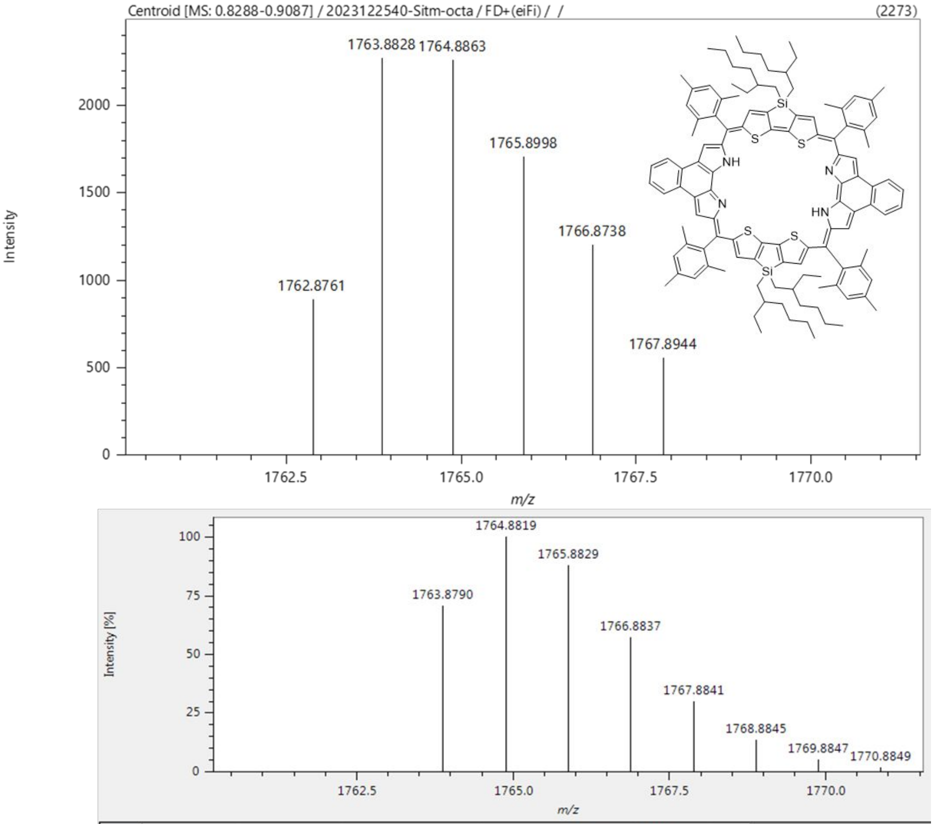

Mass	Intensity	Formula	Calculated Mass	Mass Difference [mDa]	Mass Difference [ppm]	DBE
1763.88282	2272.91	C116 H131 N4 Si2 S4	1763.87896	3.86	2.19	55.5

^{1}H NMR of **compound Si2F-2H**

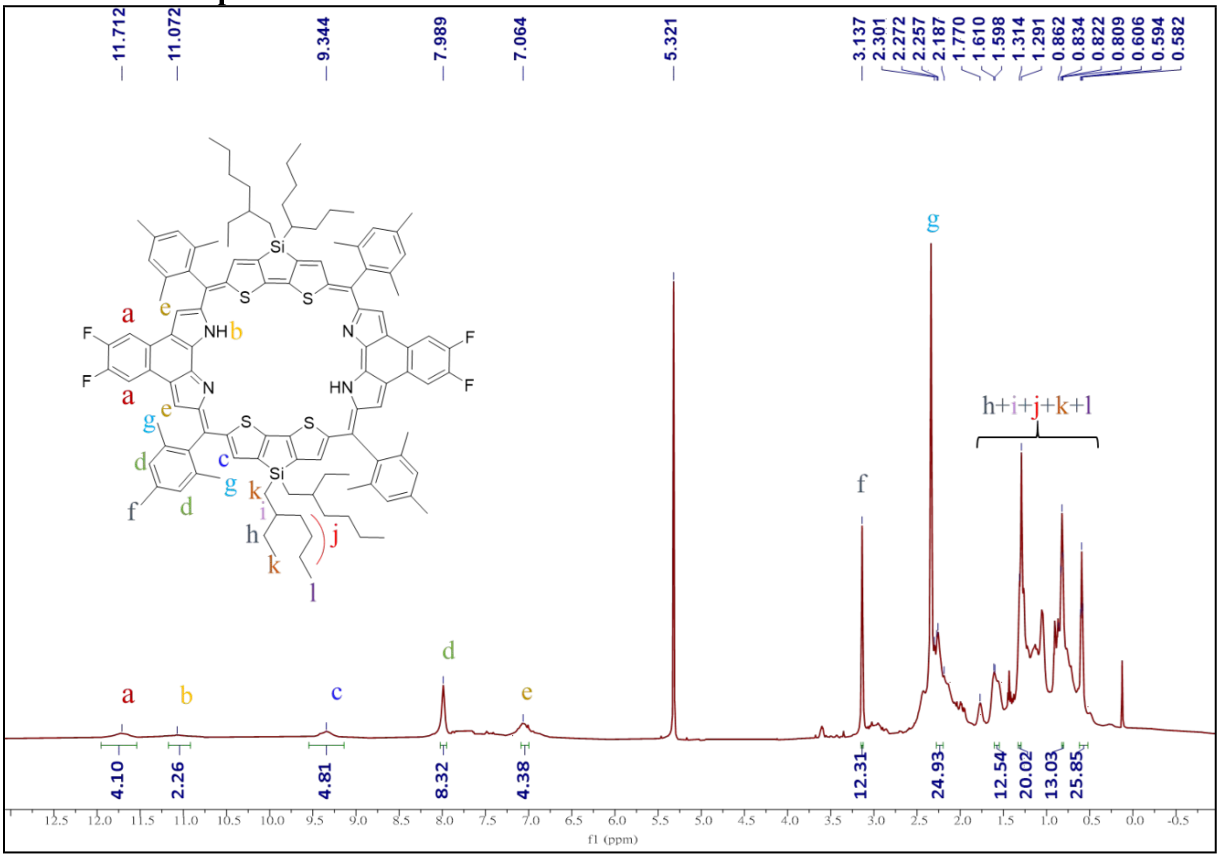

^{13}C NMR of **compound Si2F-2H**

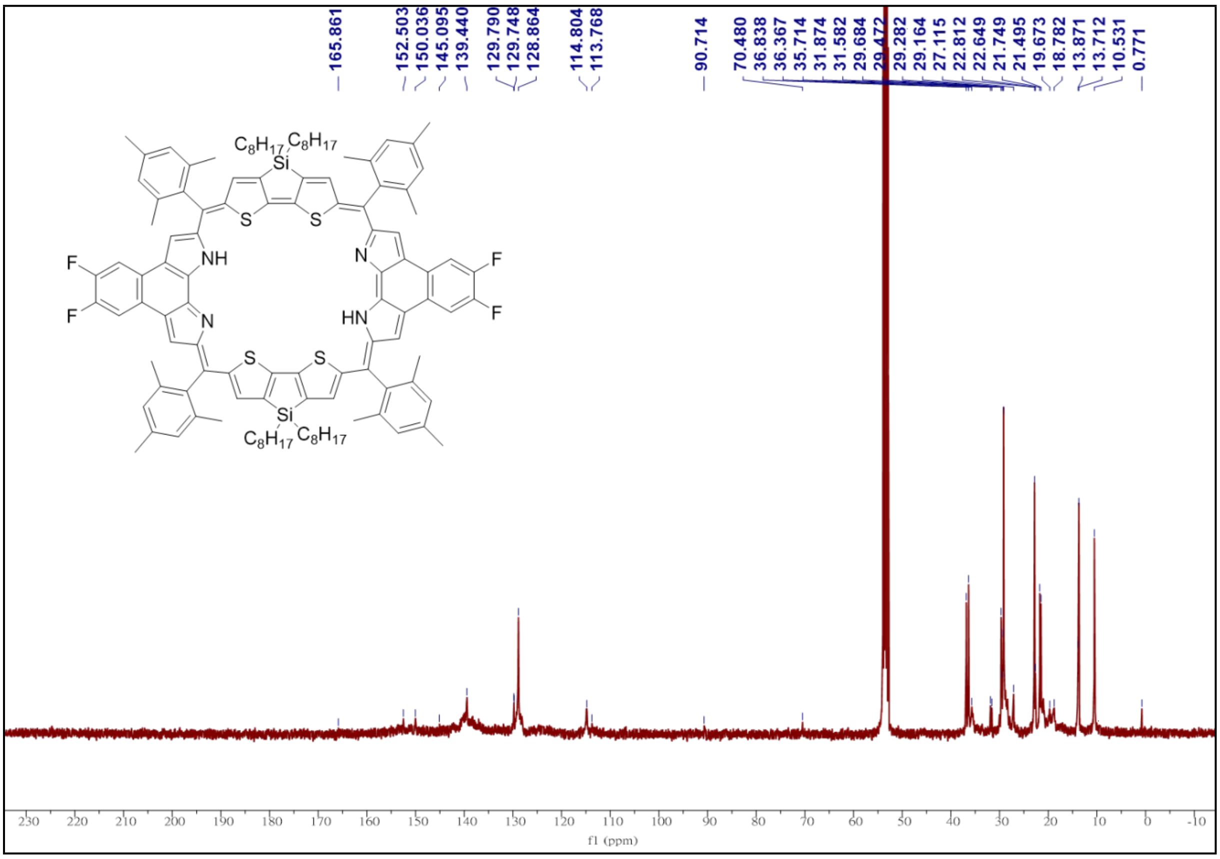

^{19}F NMR of **compound Si2F-2H**

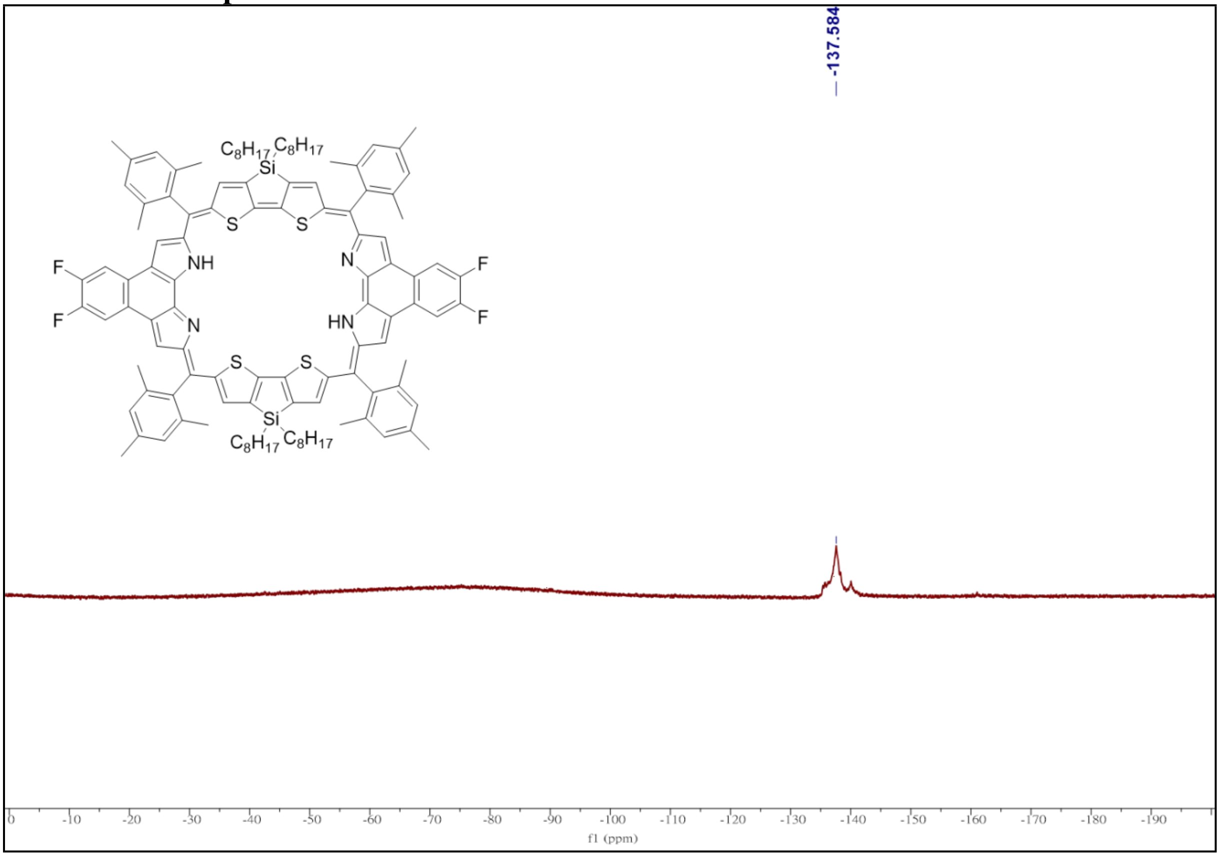

HR-Mass of **compound Si2F-2H**

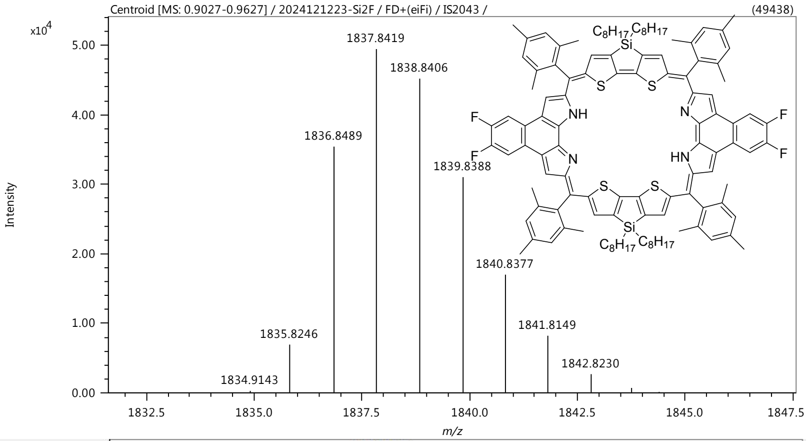

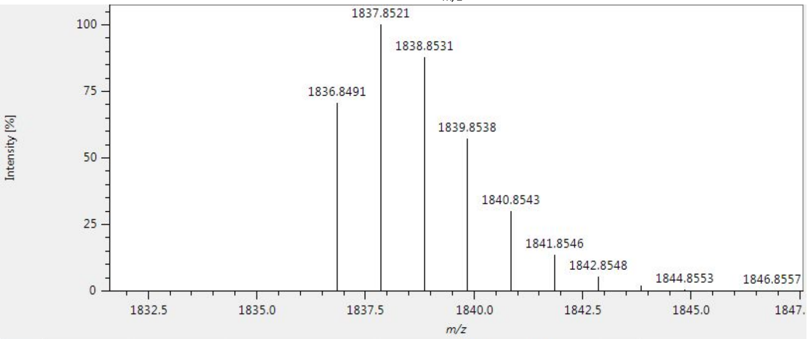

Mass	Intensity	Formula	Calculated Mass	Mass Difference [mDa]	Mass Difference [ppm]	DBE
1836.84894	35373.11	C116 H128 N4 F4 Si2 S4	1836.84910	-0.16	-0.09	55.0

^{1}H NMR of **compound S2F-2H**

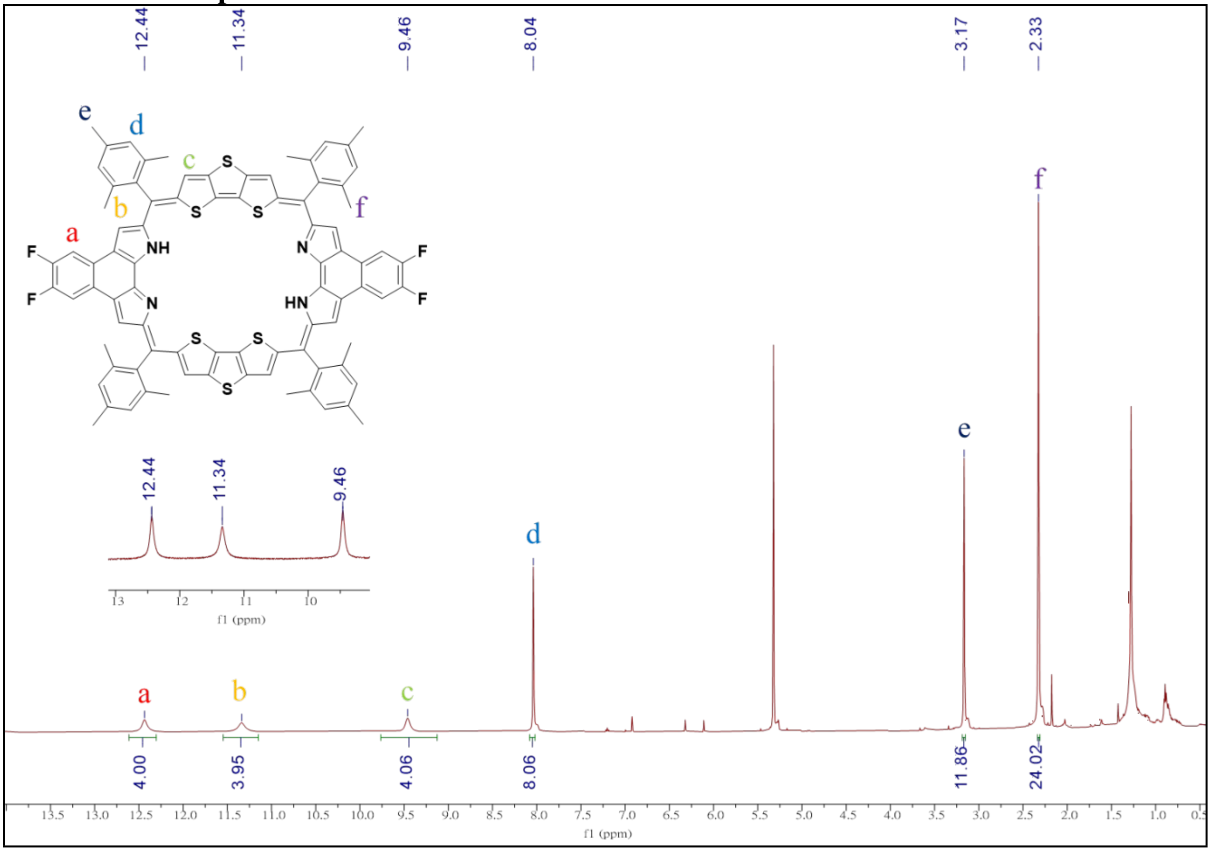

^{13}C NMR of **compound S2F-2H**

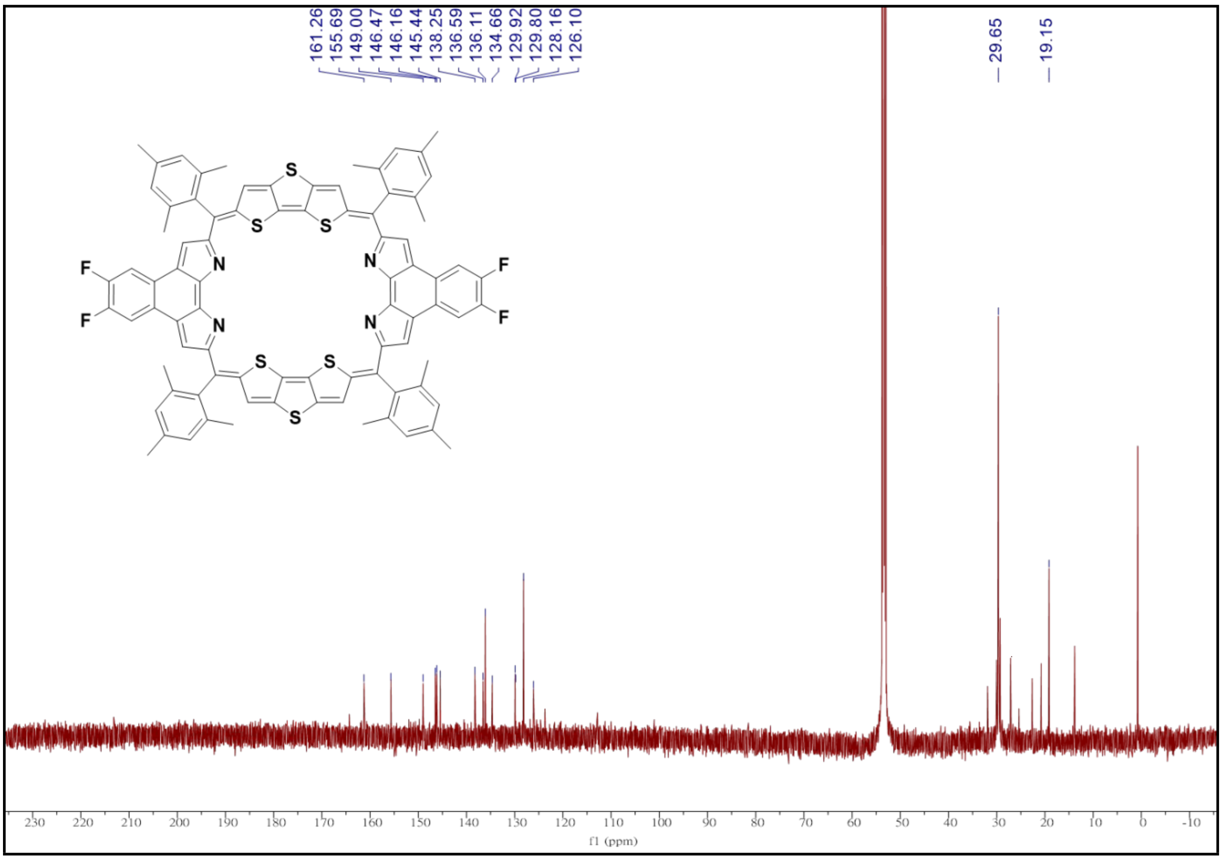

^{19}F NMR of **compound S2F-2H**

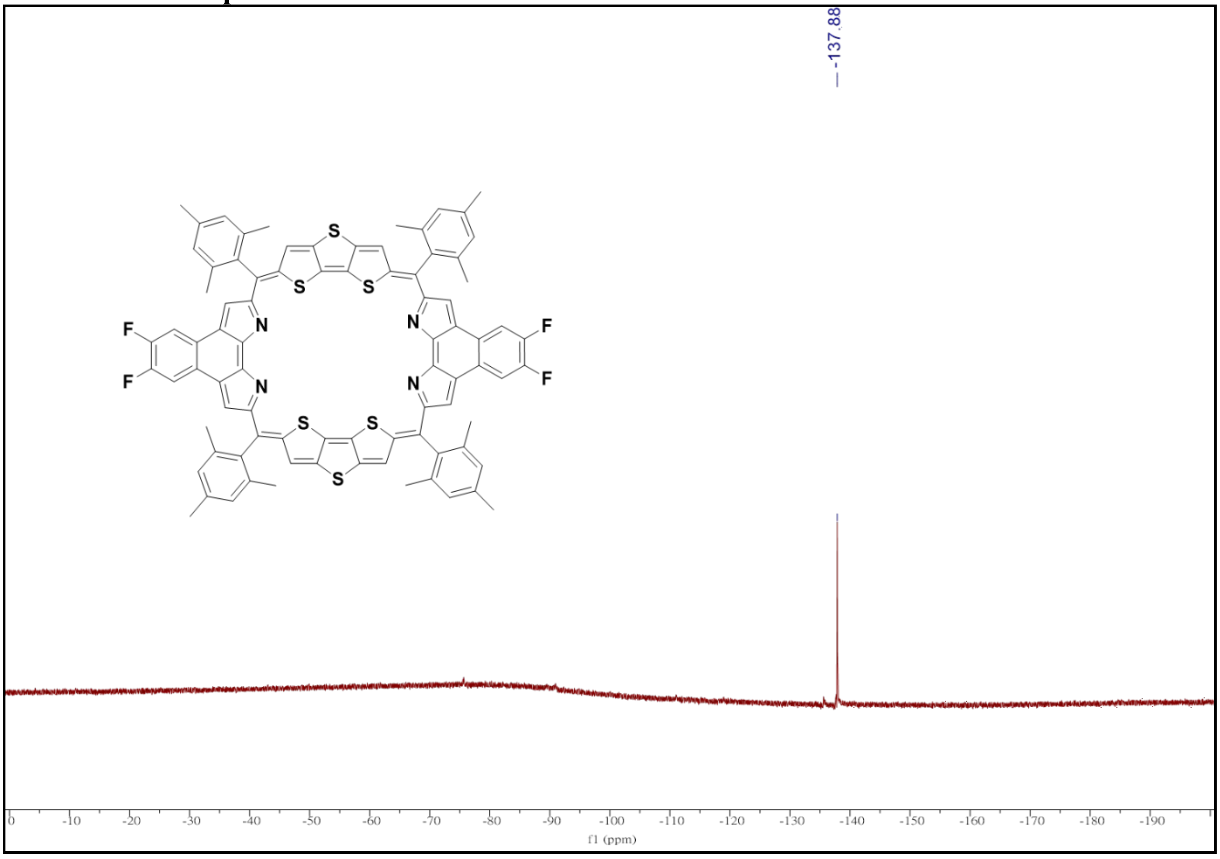

HR-Mass of **compound S2F-2H**

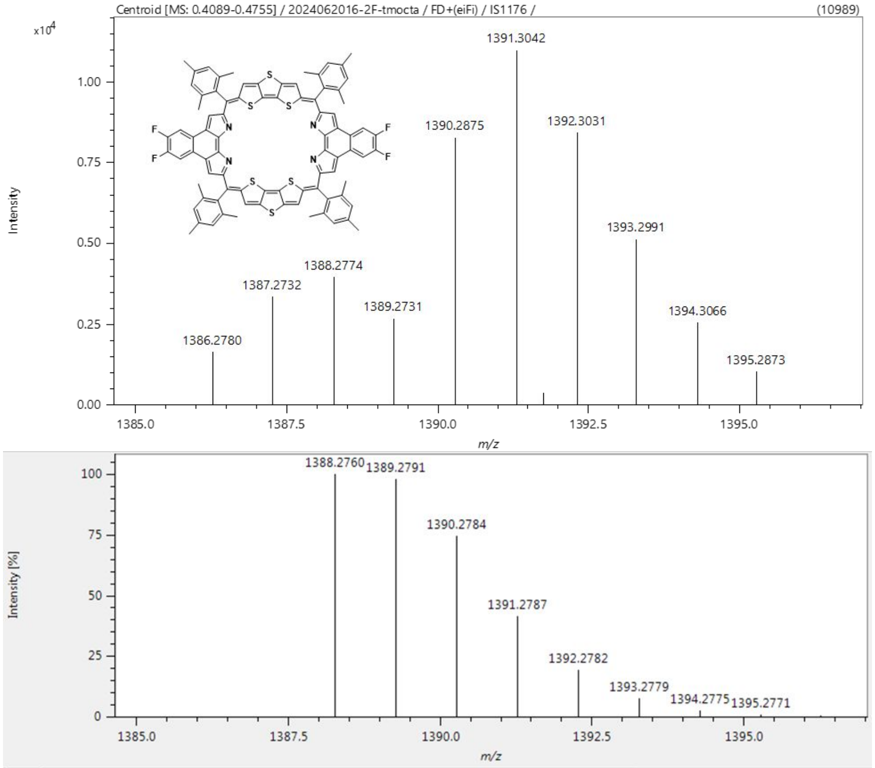

Mass	Intensity	Formula	Calculated Mass	Mass Difference [mDa]	Mass Difference [ppm]	DBE
1388.27742	3956.57	C84 H56 N4 F4 S6	1388.27599	1.44	1.04	57.0

^{1}H NMR of **compound 22**

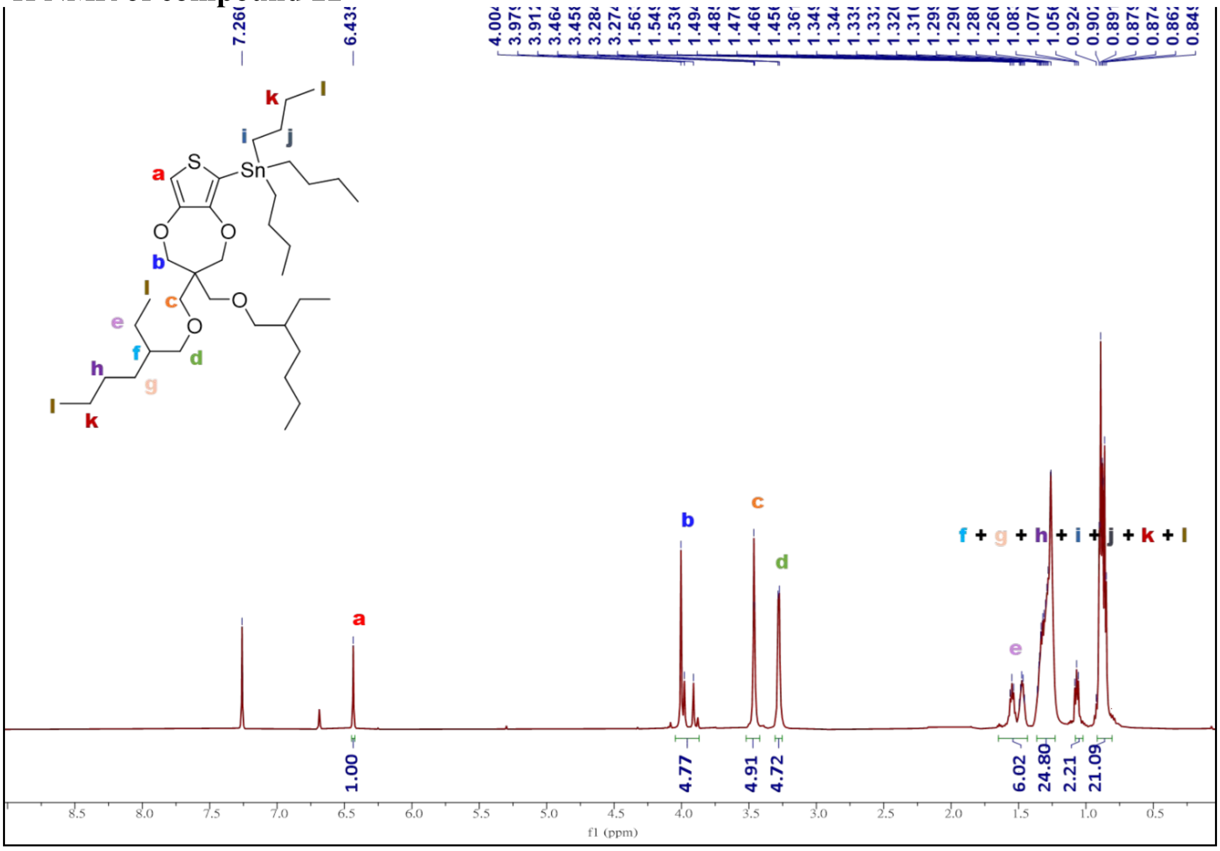

^{13}C NMR of **compound 22**

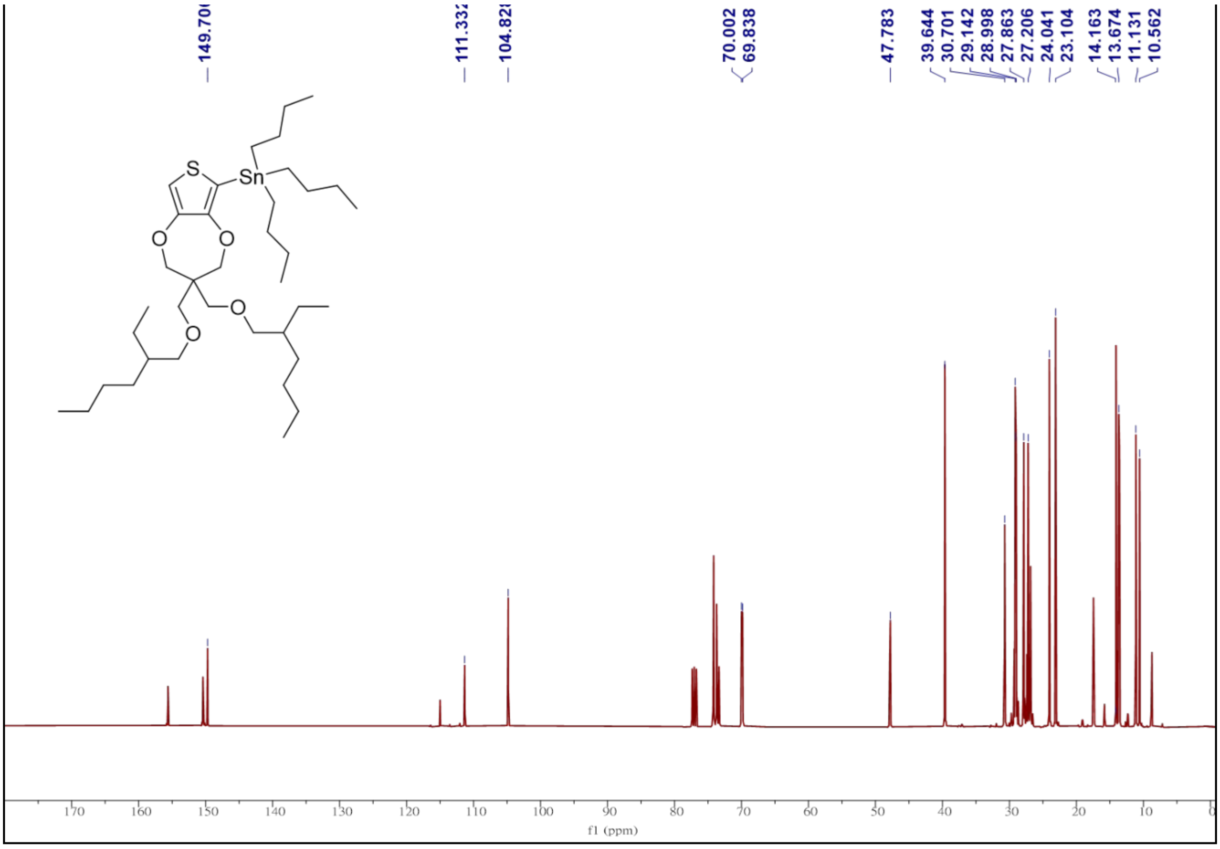

HR-Mass of **compound 22**

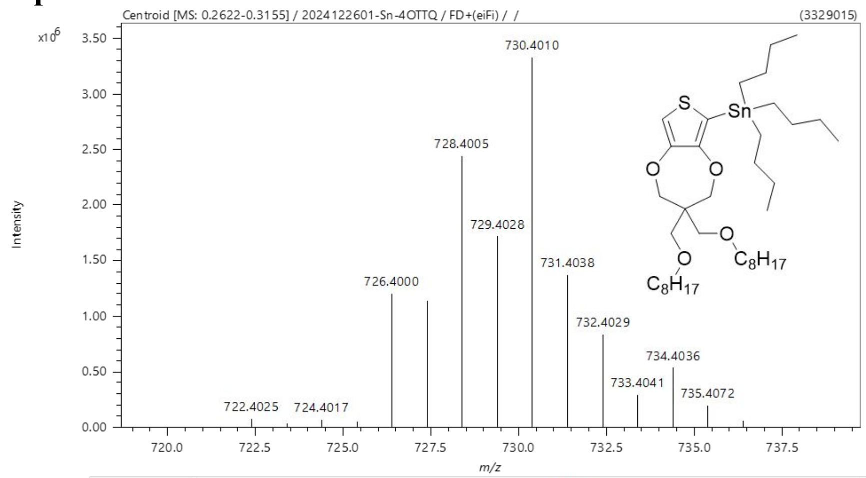

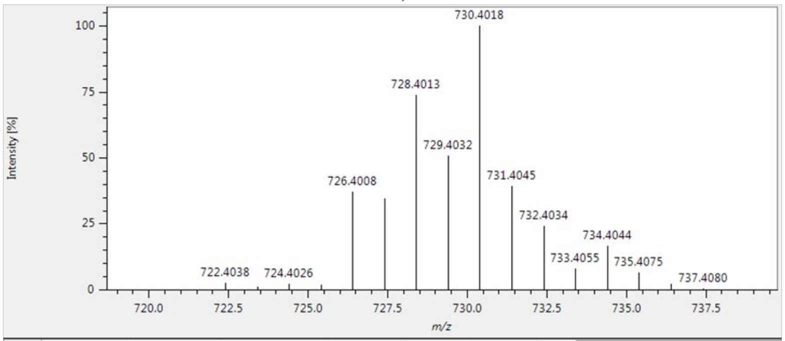

Mass	Intensity	Formula	Calculated Mass	Mass Difference [mDa]	Mass Difference [ppm]	DBE
730.40097	3329014.77	C37 H70 O4 S Sn	730.40113	-0.16	-0.22	4.0

^{1}H NMR of **compound 23**

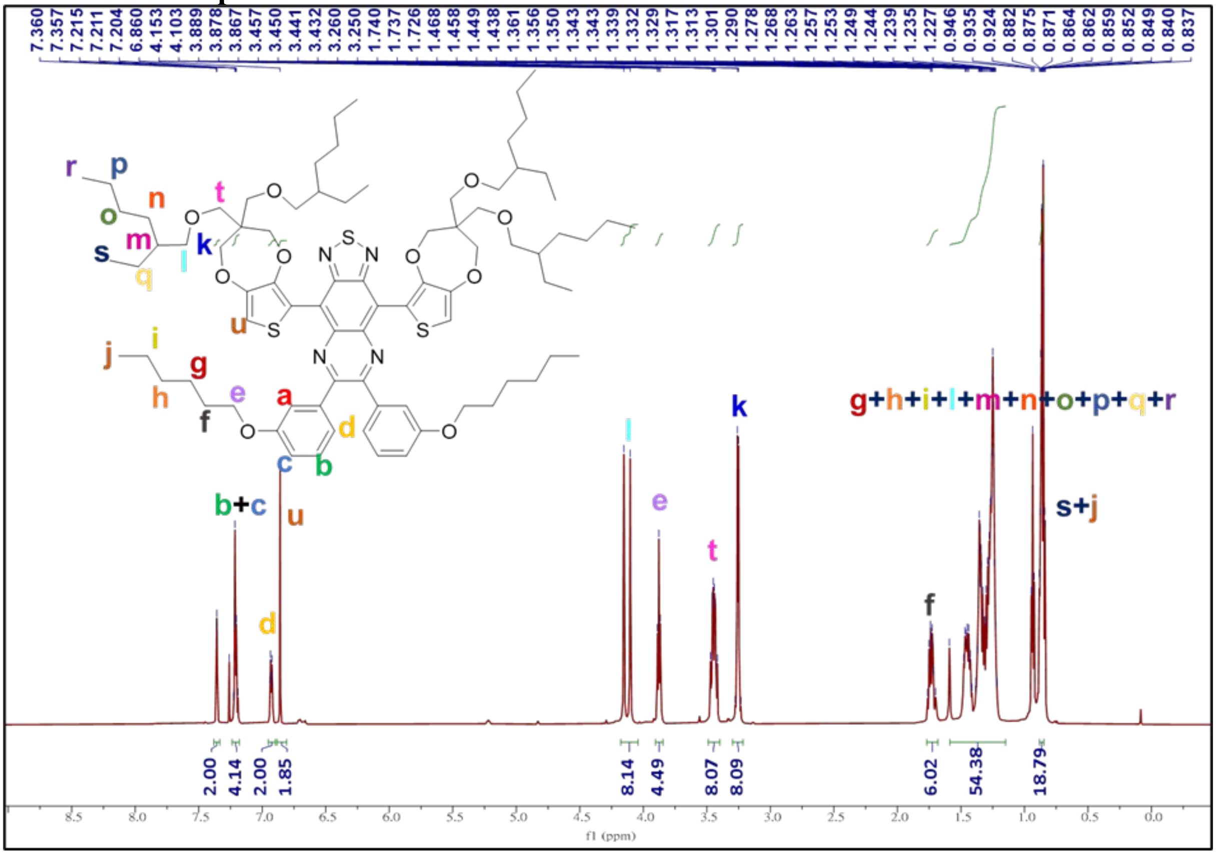

^{13}C NMR of **compound 23**

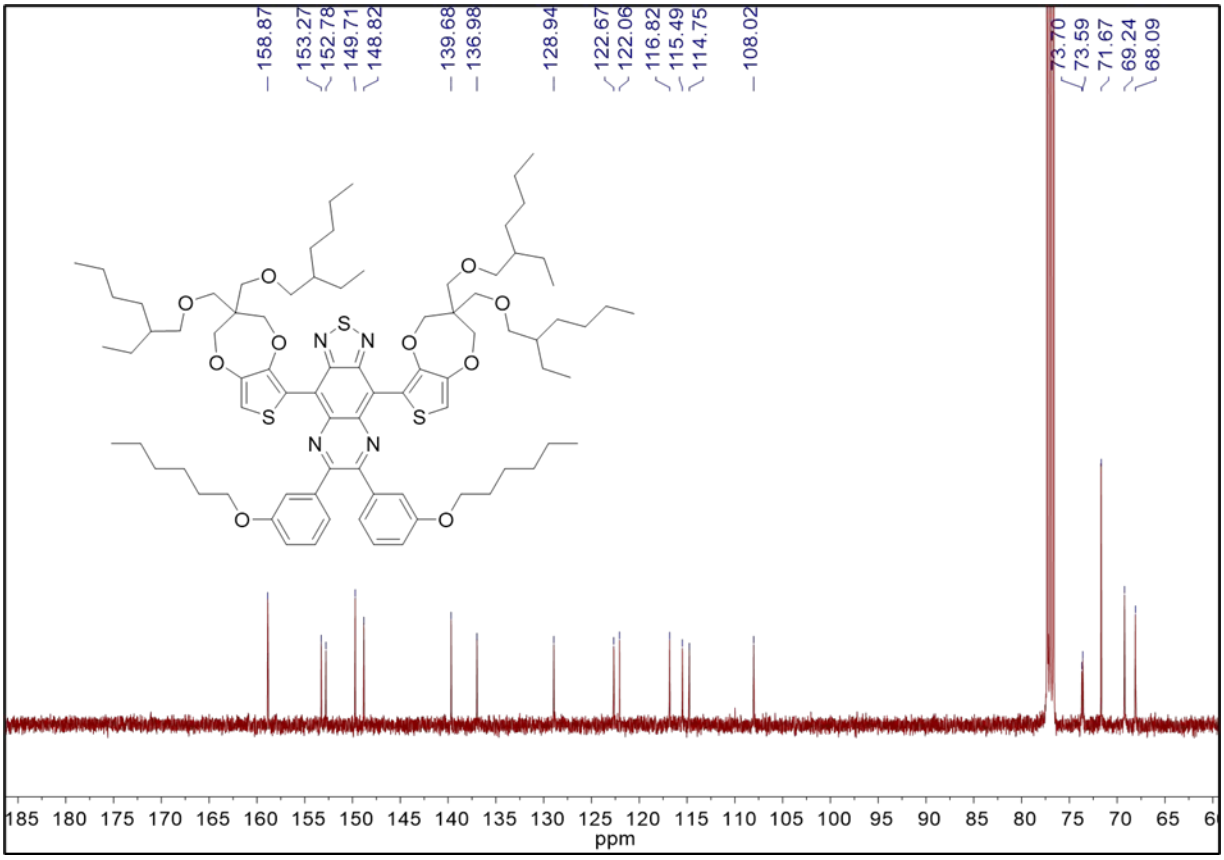

HR-Mass of **compound 23**

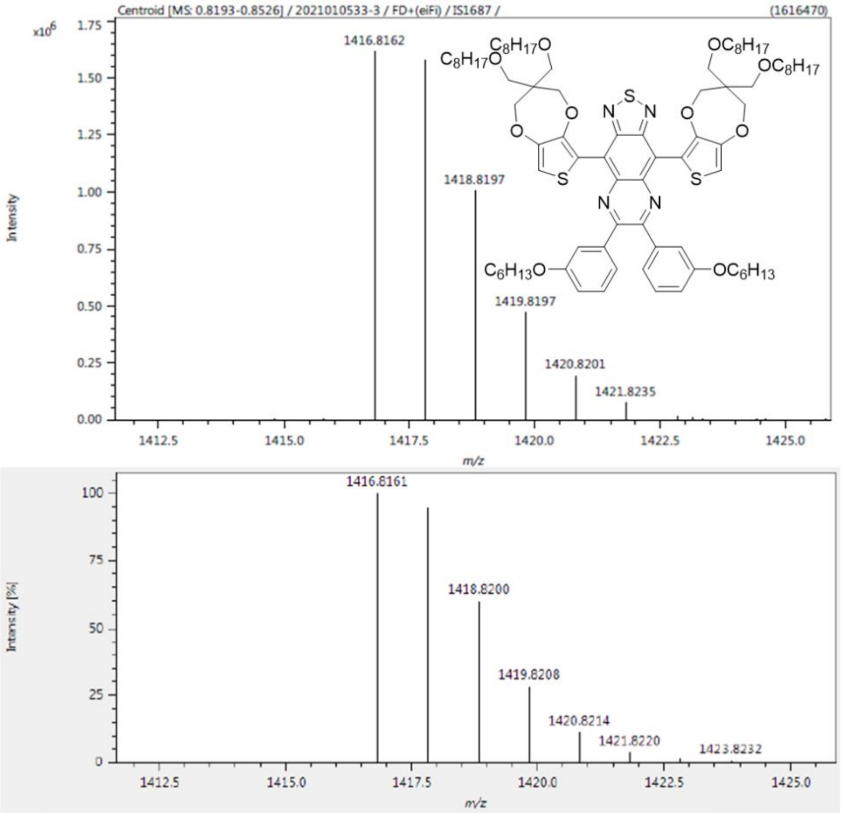

	Mass	Intensity	Formula	Calculated Mass	Mass Difference [mDa]	Mass Difference [ppm]	DBE
▸	1416.81621	1616470.08	C82 H120 N4 O10 S3	1416.81611	0.10	0.07	25.0

^{1}H NMR of **compound 24**

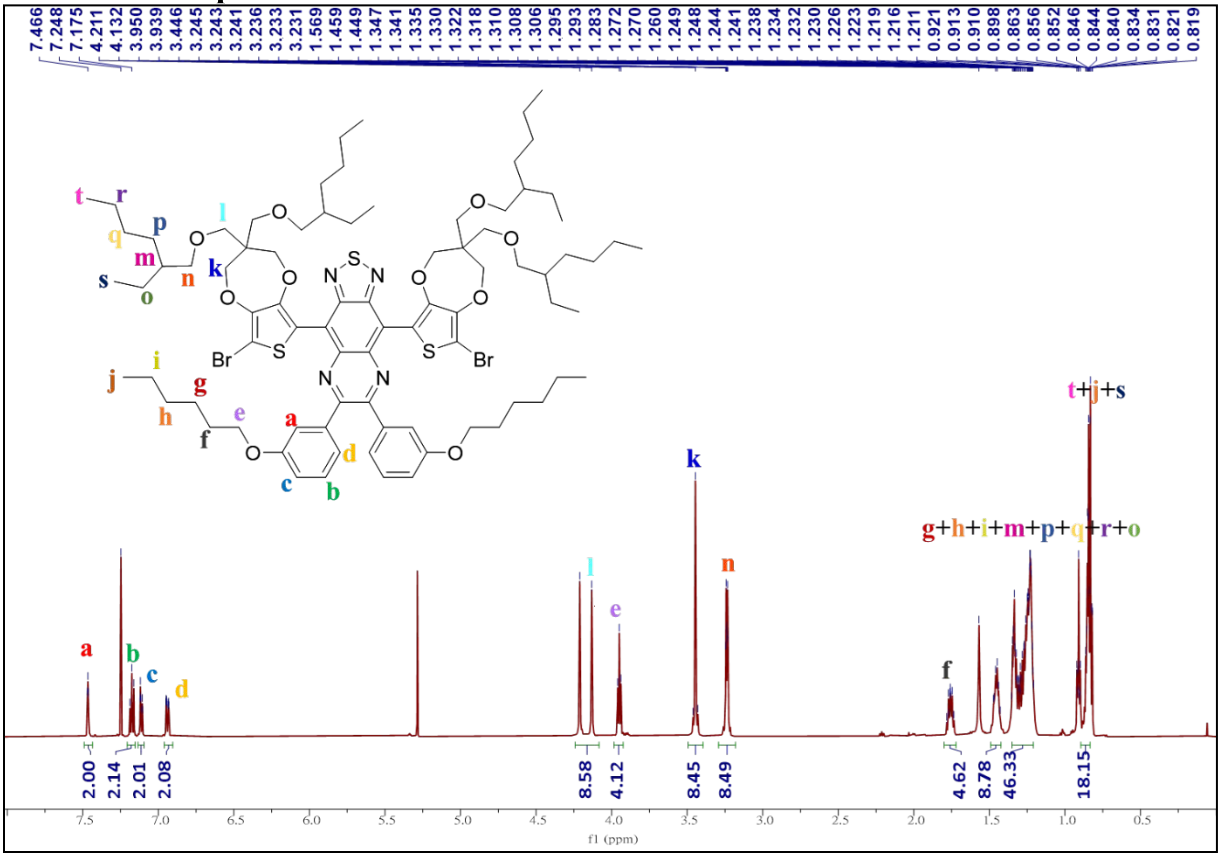

^{13}C NMR of **compound 24**

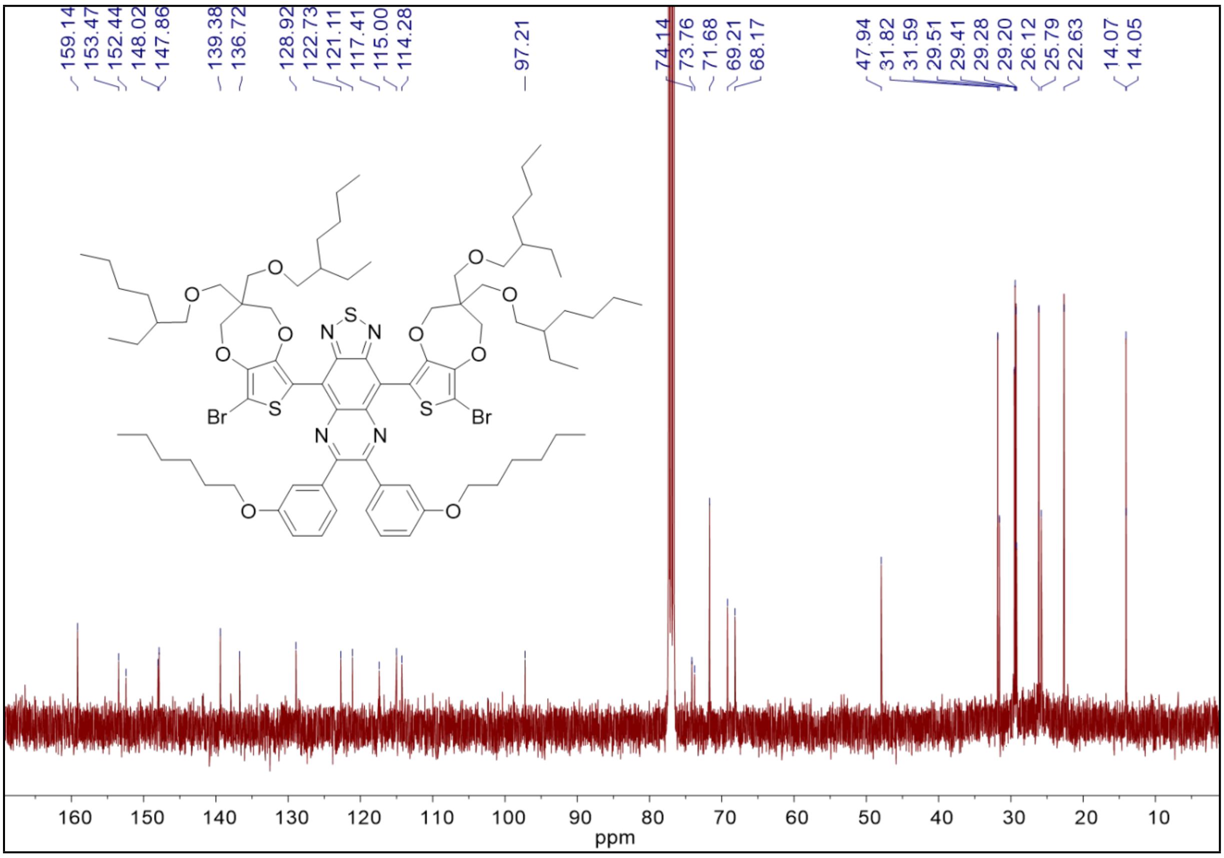

HR-Mass of **compound 24**

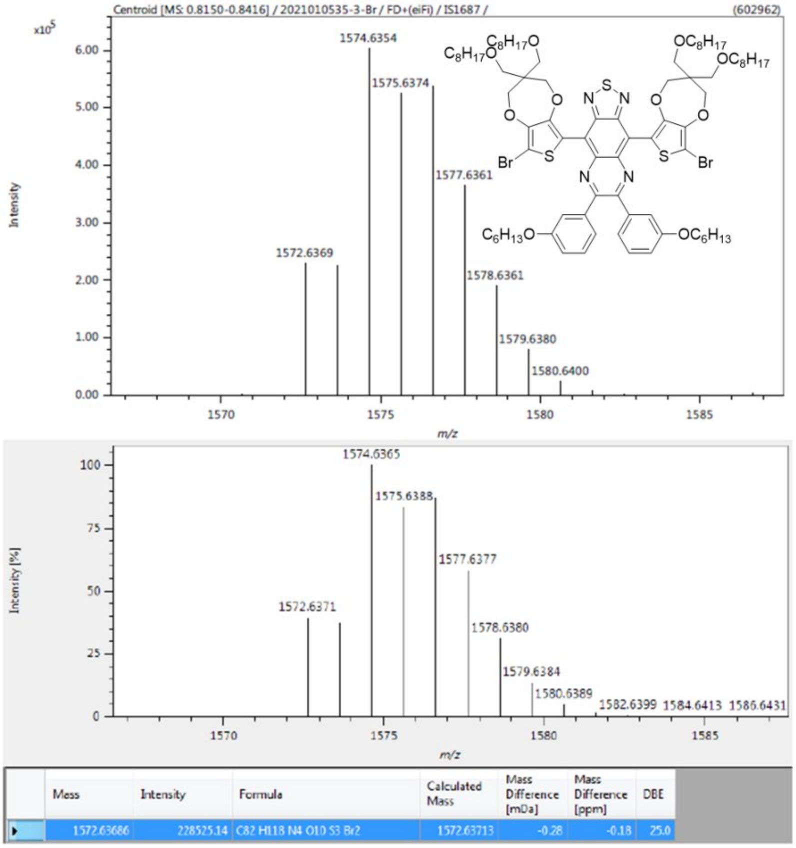

^{1}H NMR of **compound S-2H**

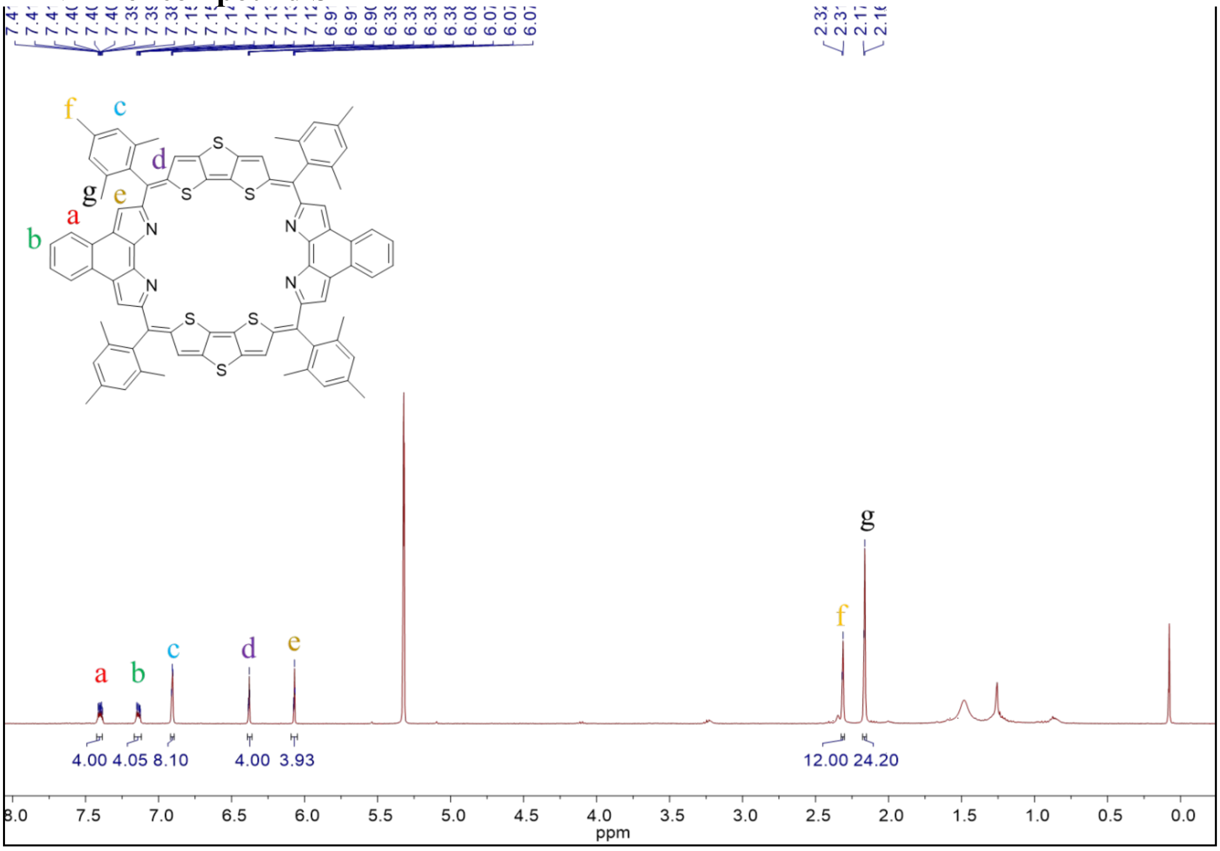

LR-FD of **compound S-2H**

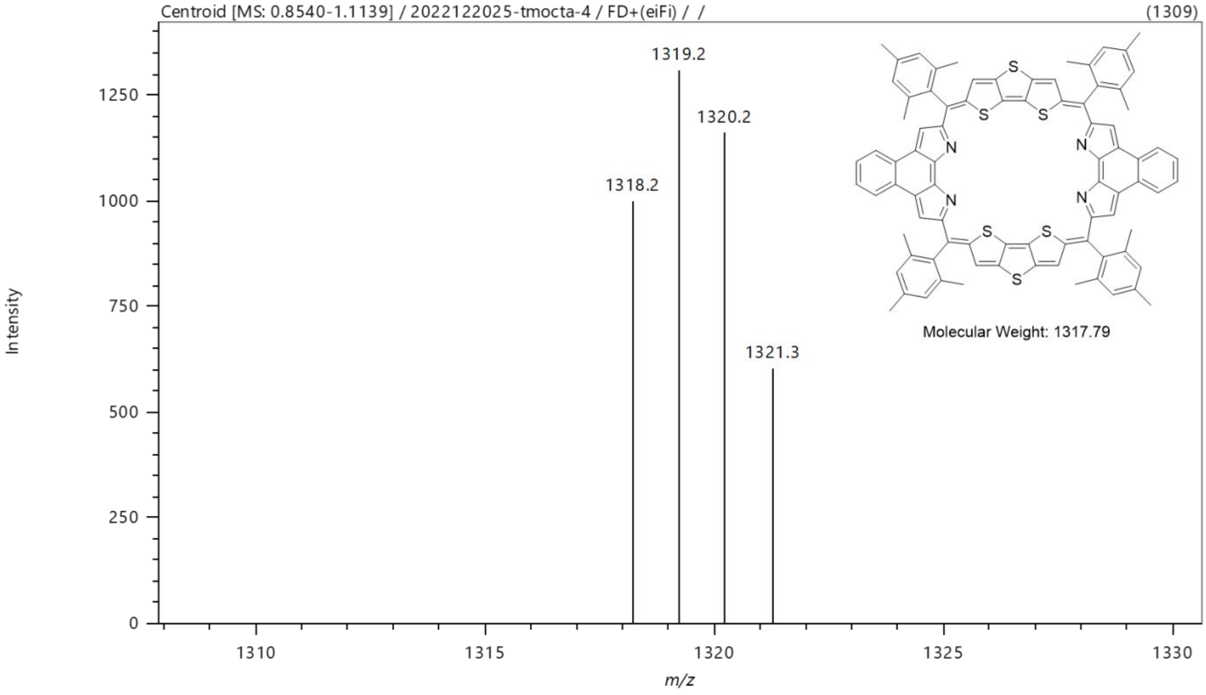

^{1}H NMR of **compound SBT-2H^{+}**

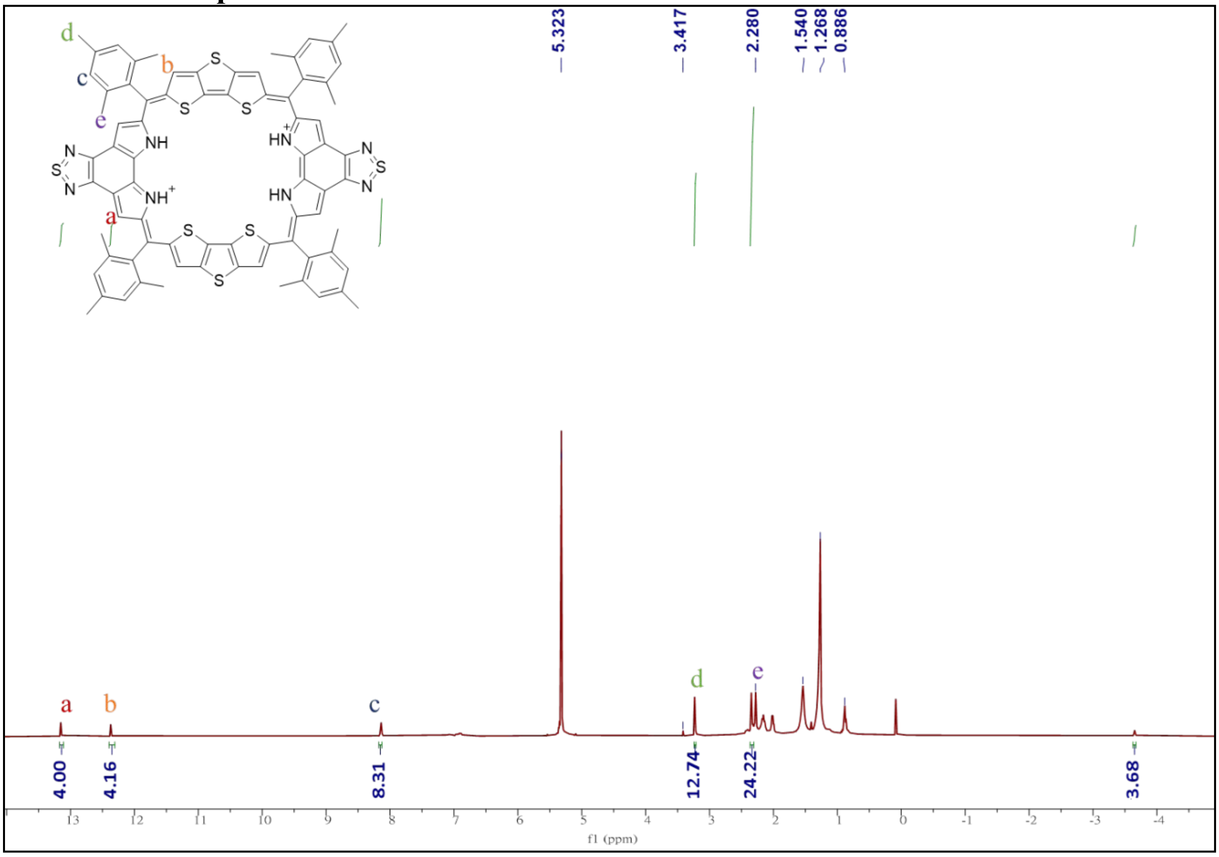

HR-MASS of **compound SBT-2H^{+}**

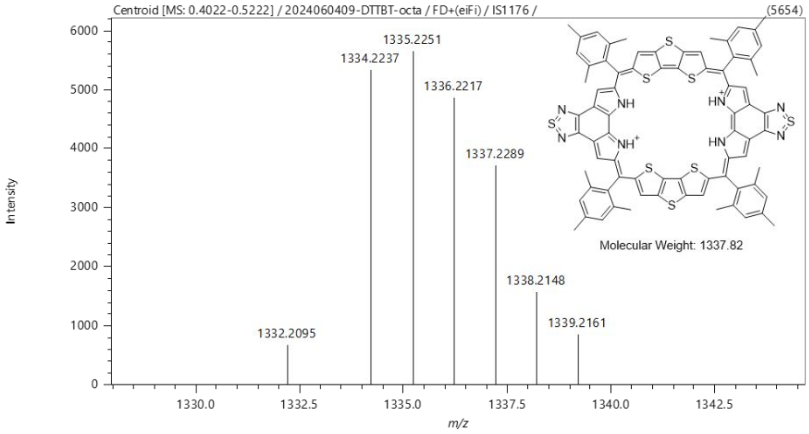

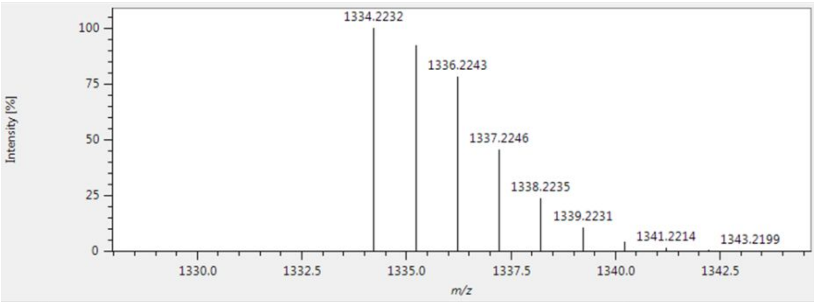

Mass	Intensity	Formula	Calculated Mass	Mass Difference [mDa]	Mass Difference [ppm]	DBE
1334.22375	5327.04	C76 H54 N8 S8	1334.22316	0.59	0.44	54.0

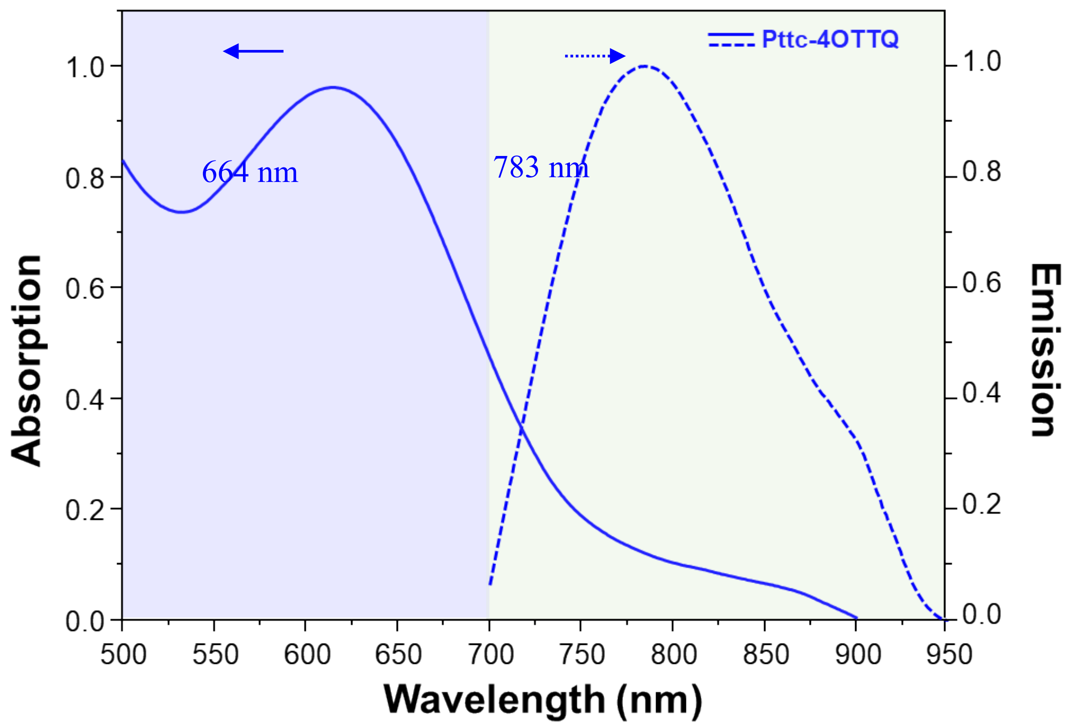

Figure S1. The absorption and emission spectra of **Pttc-4OTTQ** in CH_2Cl_2.

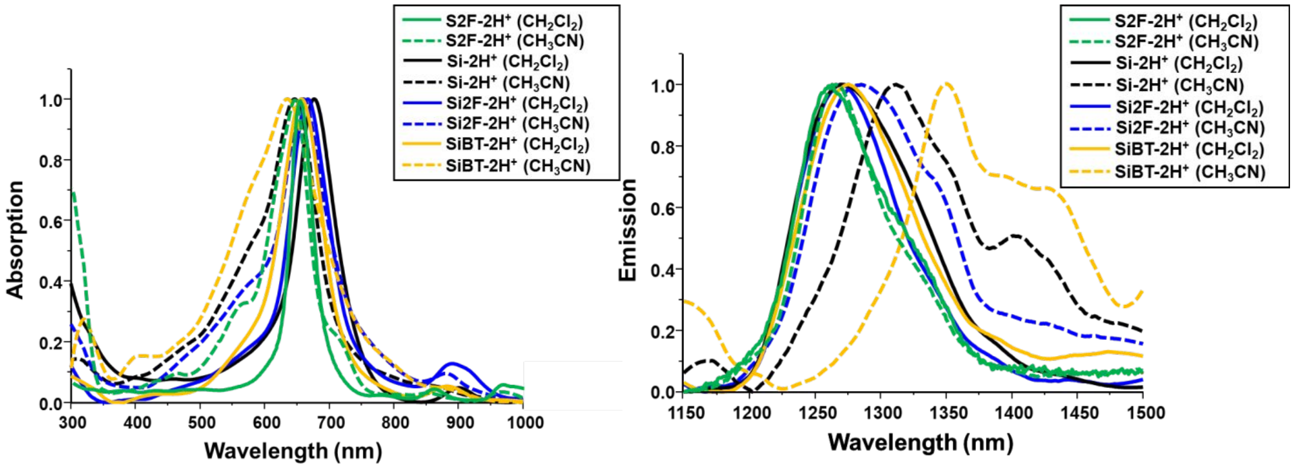

Figure S2. The absorption (left) and emission (right) spectra of porphyrinoids in CH_2Cl_2 and CH_3CN.

Computational Detail. All geometry optimization and frequency analyses of **Si-2H$^+$**, **Si2F-2H$^+$**, **SiBT-2H$^+$** and **S2F-2H$^+$**, both in the ground state (and the first excited state) using DFT (TDDFT) at the B3LYP-D3/6-31G* level. The *n*-alkyl groups for **Si-2H$^+$**, **Si2F-2H$^+$**, **SiBT-2H$^+$**, and **O4TTQ** are replaced by methyl or ethyl groups for better computational efficiency. The structure optimization calculation and frequency analysis of the **dye•4OTTQ** complex were also completed in B3LYP-D3/6-31G*. Theoretical absorption optical properties have been calculated on ground state geometries using TD-DFT/6-31G* in different exchange–correlation functionals, such as B3LYP, PBE0, and PBE38[12], with CH_2Cl_2 as solvent using the conductor-like polarizable continuum model (CPCM)[13]. The calculated results are listed in **Table S3**. The Q band predicted by PBE38 is closer to the experimental observations. Therefore, all TD-DFT excited state calculations were performed using PBE38 functional with 6-31G* basis sets. Theoretical absorption optical properties have been calculated on ground state geometries using TD-PBE38/6-31G* with CH_2Cl_2 as solvent using the conductor-like polarizable continuum model (CPCM). Pentyl acetate is used to model with **Pdot** condition which is the aliphatic part of mPEG-DSPE. All the DFT calculations were carried out employing Gaussian 16 Revision A.03. (Gaussian 16, Revision A.03, Frisch, M. J.; Trucks, G. W.; Schlegel, H. B.; Scuseria, G. E.; Robb, M. A.; Cheeseman, J. R.; Scalmani, G.; Barone, V.; Petersson, G. A.; Nakatsuji, H.; Li, X.; Caricato, M.; Marenich, A. V.; Bloino, J.; Janesko, B. G.; Gomperts, R.; Mennucci, B.; Hratchian, H. P.; Ortiz, J. V.; Izmaylov, A. F.; Sonnenberg, J. L.; Williams-Young, D.; Ding, F.; Lipparini, F.; Egidi, F.; Goings, J.; Peng, B.; Petrone, A.; Henderson, T.; Ranasinghe, D.; Zakrzewski, V. G.; Gao, J.; Rega, N.; Zheng, G.; Liang, W.; Hada, M.; Ehara, M.; Toyota, K.; Fukuda, R.; Hasegawa, J.; Ishida, M.; Nakajima, T.; Honda, Y.; Kitao, O.; Nakai, H.; Vreven, T.; Throssell, K.; Montgomery, J. A., Jr.; Peralta, J. E.; Ogliaro, F.; Bearpark, M. J.; Heyd, J. J.; Brothers, E. N.; Kudin, K. N.; Staroverov, V. N.; Keith, T. A.; Kobayashi, R.; Normand, J.; Raghavachari, K.; Rendell, A. P.; Burant, J. C.; Iyengar, S. S.; Tomasi, J.; Cossi, M.; Millam, J. M.; Klene, M.; Adamo, C.; Cammi, R.; Ochterski, J. W.; Martin, R. L.; Morokuma, K.; Farkas, O.; Foresman, J. B.; Fox, D. J. Gaussian, Inc., Wallingford CT, 2016.)

Detailed analysis of Pdot modeled dyes. Using **S2F-2H$^+$** as a representative example, **Scheme S3** illustrates the proposed stacking configurations between **S2F-2H$^+$** and **4OTTQ**, which maximizes contact between the two components. Two distinct stacking modes were explored: i) a-stacked: Thiadiazole group oriented toward the indole hydrogens of **4OTTQ**; and ii) b-stacked: Thiadiazole group aligned with the thiophene moiety of **4OTTQ**. For the Si series, structural analysis focused exclusively on bowl-shaped configurations stacked with **4OTTQ**. For **S2F-2H$^+$**, both bowl-shaped and wave-shaped conformations were evaluated for their stacking interactions with **4OTTQ**.

However, attempts to stabilize the b-stacked wave-shaped **S2F-2H$^+$-4OTTQ** complex were unsuccessful. As a results. two stable complexes were identified for the Si series and three for **S2F-2H$^+$**. **Scheme S4** presents the optimized structures and BSSE-corrected binding energies with their top-view. For Si Series, all complexes except the a_stacked **SiBT-2H$^+$** deviate from the bowl-shaped geometry. To investigate how the structural alteration through Pdot formation might affect the optical properties, the HOMO-LUMO gaps and excitation properties of dyes were derived by extracting the dye's geometry (with the chloride ions) from dye•**4OTTQ** complexes, with pentyl acetate, a weakly polar solvent, mimicking the dielectric effects of surfactant in Pdot. The results are summarized in **Table S5** and **Figure S7**. From these results, blueshift for most Si-containing cases, except the a_stacked **Si-2H$^+$** and redshift for **SiBT-2H$^+$** and **S2F-2H$^+$** relative to their original structures of CH_2Cl_2 are seen. To eliminate the influence of solvent on absorption wavelengths, we performed absorption optical calculations for these porphyrinoids within the Pdot form using CH_2Cl_2 as the solvent (**Table S5**). The results reveal that all dyes in CH_2Cl_2 exhibit nearly identical behaviors similar to pentyl acetate, with only a minor redshift in the Soret band. This consistency confirms that structural modifications of the dye within Pdots are the primary driver of optical property changes rather than the solvent effect. As a result, the observed redshift in **S2F-2H$^+$** Pdots likely arises from the wave-shaped **S2F-2H$^+$** adopting a bowl-like conformation. A larger Soret band blueshift in **SiBT-2H$^+$** Pdots occurs only when **4OTTQ** stacks with **SiBT-2H$^+$** in the b_mode configuration. The molecular orbital energy diagram of these porphyrinoids in the Pdot form is also summarized in **Figure S7**. It is seen that the distorted structure of Si series resulting in an increase in the gap between HOMO and LUMO, contributing to a blue shift in the spectra. Only in the case of **S2F-2H$^+$** did we see a decreased HOMO-LUMO gap due to the conformation change.

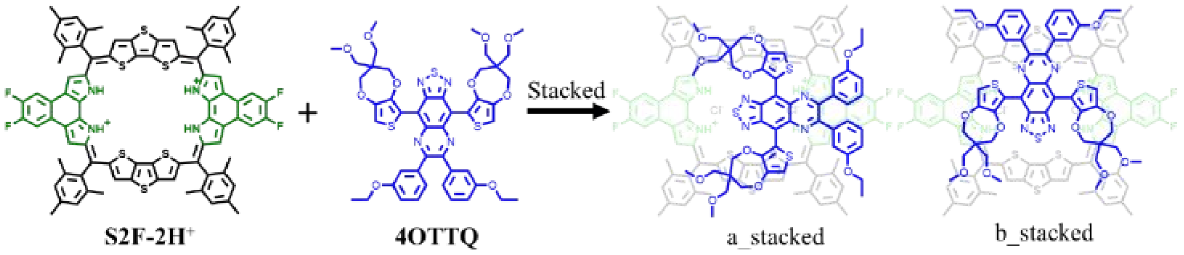

Scheme S3. Schematic diagram of a simple **4OTTQ** and **Si2F-2H$^+$** stacking design.

Table S1. BSSE corrected binding energy (kcal/mol) of the deprotonated dyes with the two chloride ions, calculated at the M06-2X(CPCM)/6-31+G*//B3LYP-D3/6-31G* level. Data in water indicating the strong association remains even with a strongly dipolar solvent.

		**in CH_2Cl_2**	**in water**
**Si-2H$^+$**	bowl-shaped	-43.62	-21.20
	wave-shaped	-42.33	-22.75
**SiBT-2H$^+$**	bowl-shaped	-43.06	-22.88
	wave-shaped	-40.01	-19.71
**Si2F-2H$^+$**	bowl-shaped	-41.70	-22.16
	wave-shaped	-38.69	-19.04
**S2F-2H$^+$**	bowl-shaped	-44.49	-23.89
	wave-shaped	-40.81	-20.10

Table S2. Absorption wavelength (nm) from experiments and TDDFT(PBE38) calculation, for **Si-2H$^+$**, **Si2F-2H$^+$**, **SiBT-2H$^+$** and **S2F-2H$^+$** in their diprotonated, dicationic form or dichloride-incorporated, charge-neutral forms (calculated with CH_2Cl_2 as a model solvent).

	**Experiment**		**Diprotonated**		**Chloride-incorporated**	
	Soret band	Q band	B band[a]	Q band	B band[a]	Q band
**Si-2H$^+$**	664	1259	631	1208	633	1243
**SiBT-2H$^+$**	666	1279	623	1224	625	1261
**Si2F-2H$^+$**	670	1272	629	1219	631	1258
**S2F-2H$^+$**	656	1237	610	1153	615	1190
					606[a]	1150[a]

[a]Wave-shaped structure

Table S3. Absorption wavelength (nm) of **Si-2H$^+$**, **Si2F-2H$^+$**, **SiBT-2H$^+$** and **S2F-2H$^+$** with dichloride incorporated, calculated with TDDFT with 3 different density functionals, with CH_2Cl_2 as a model solvent.

	**Experimental Data (CH_2Cl_2)**		**B3LYP**		**PBE0**		**PBE38**	
	Soret band	Q band	Soret band	Q band	Soret band	Q band	Soret band	Q band
**Si-2H$^+$**	664	1259	661	1184	651	1185	633	1243
**SiBT-2H$^+$**	666	1279	651	1194	641	1197	625	1261
**Si2F-2H$^+$**	670	1272	658	1195	649	1197	631	1258
**S2F-2H$^+$**	656	1237	642	1144	632	1143	615	1190
			632	1108[a]	621[a]	1107[a]	606[a]	1150[a]

[a] Wave-shaped structure.

Table S4. Reorganization energies (meV) of diprotonated **Si-2H$^+$**, **SiBT-2H$^+$**, **Si2F-2H$^+$** and **S2F-2H$^+$** at B3LYP-D3/6-31G* level

	λ^{ground}	λ^{ex}	λ
**Si-2H$^+$**	14.1	13.5	13.8
**SiBT-2H$^+$**	17.6	16.3	17.0
**Si2F-2H$^+$**	15.7	15.0	15.3
**S2F-2H$^+$**	20.1	17.4	18.7

Table S5. TD-PBE38/6-31G* absorption wavelength (Soret band, nm) with dyes structures derived from their binding with 4OTTQ in mimicking **Pdot**, and with pentyl acetate (simulating surfactant environment in Pdot) **Si-2H$^+$**, **Si2F-2H$^+$**, **SiBT-2H$^+$** and **S2F-2H$^+$**. Excitation wave lengths similarly calculated for freely optimized dyes is also included. For comparison, the structure mimicking Pdot was also calculated under CH_2Cl_2, indicating minimal effects of solvent polarity.

			Pdot/pentyl acetate	Free/CH_2Cl_2	Pdot/CH_2Cl_2
**Si-2H$^+$**	major	a_stacked	627	633	629
		b_stacked	628		630
**SiBT-2H$^+$**	major	a_stacked	644	625	645
		b_stacked	620		621
**Si2F-2H$^+$**		a_stacked	623	631	625
	major	b_stacked	625		627
**S2F-2H$^+$**		a_stacked	612	615	614
		b_stacked	608		610
	major	a_stacked[a]	615	606	617

[a]This **S2F-2H$^+$** structure is obtained from the wave-structure of **S2F-2H$^+$** with **4OTTQ** optimized structure.

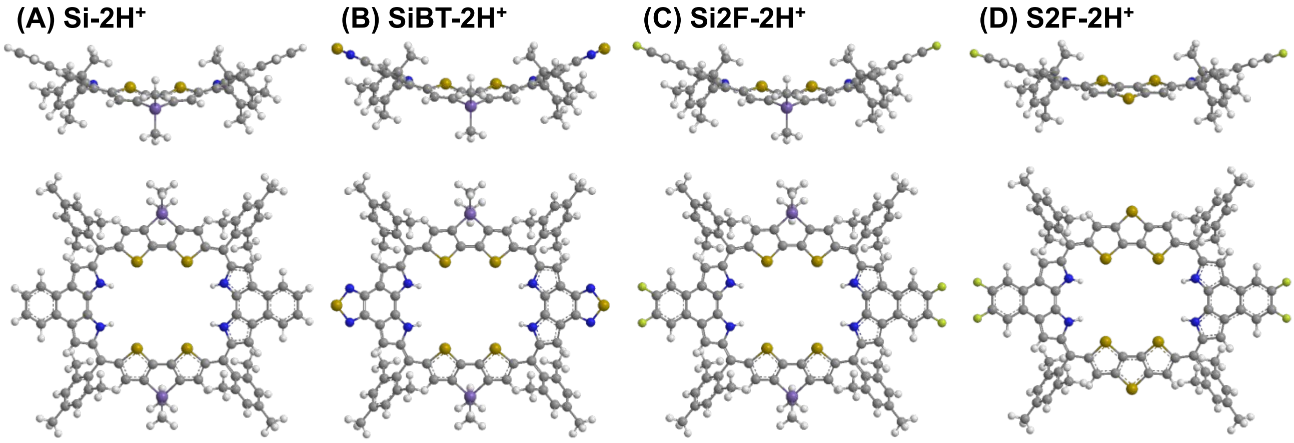

Figure S3. The optimized structures of (A) **Si-2H$^+$**, (B) **SiBT-2H$^+$**, (C) **Si2F-2H$^+$**, and (D) **S2F-2H$^+$** without two chloride ions at the theoretical level of B3LYP-D3/6-31G*. The top row is a side view and the bottom row is a top view.

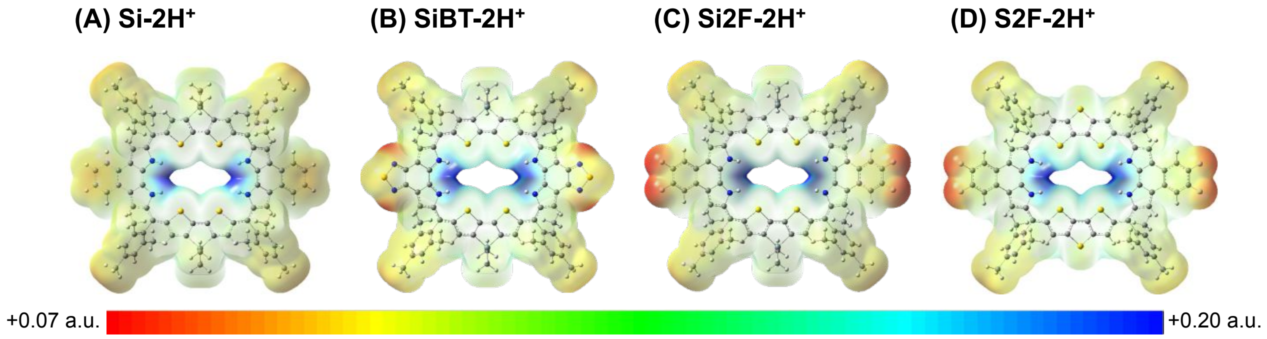

Figure S4. Molecular electrostatic potential map of (A) **Si-2H$^+$**, (B) **SiBT-2H$^+$**, (C) **Si2F-2H$^+$**, and (D) **S2F-2H$^+$** obtained from B3LYP-D3/6-31G*.

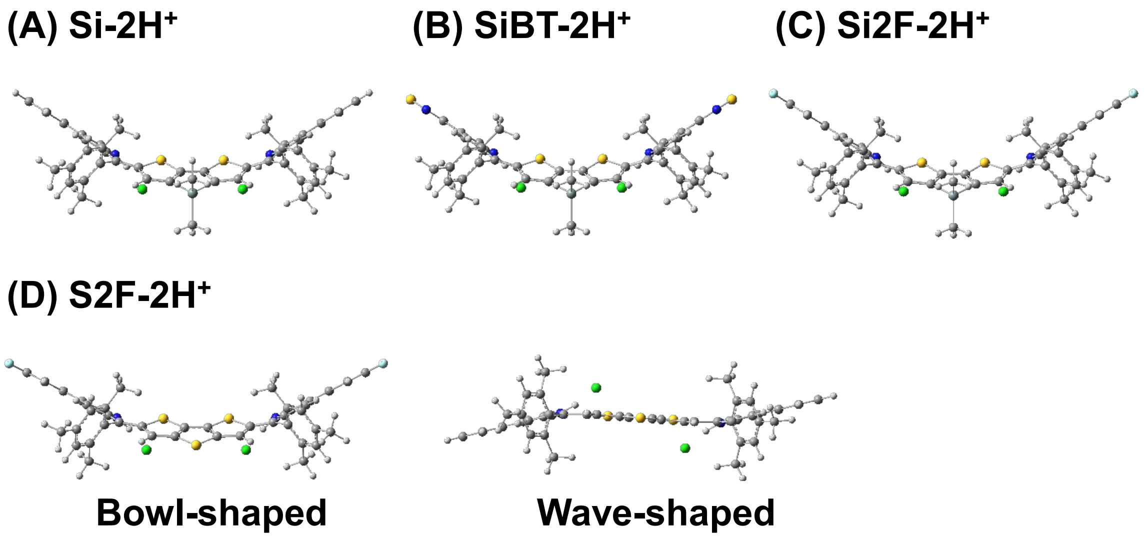

Figure S5. The side view of optimized structures of the first excited state of (A) **Si-2H$^+$**, (B) **SiBT-2H$^+$**, (C) **Si2F-2H$^+$**, and (D) **S2F-2H$^+$**, at the theoretical level of TD-B3LYP-D3/6-31G*.

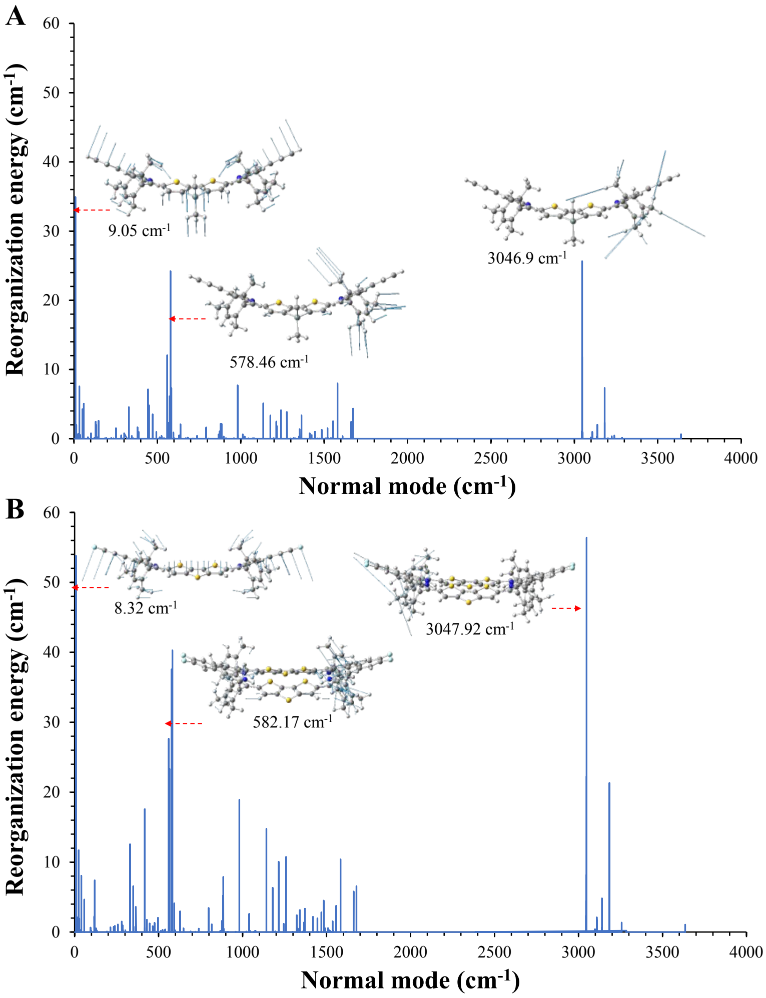

Figure S6. Reorganization of S_0 to S_1 in projection to normal modes, with estimated contribution of reorganization energy vs. normal mode frequencies plotted, for (a) **Si-2H$^+$** and (b) **S2F-2H$^+$**. The insets show the displacement vectors of the normal modes with large contribution in the transition.

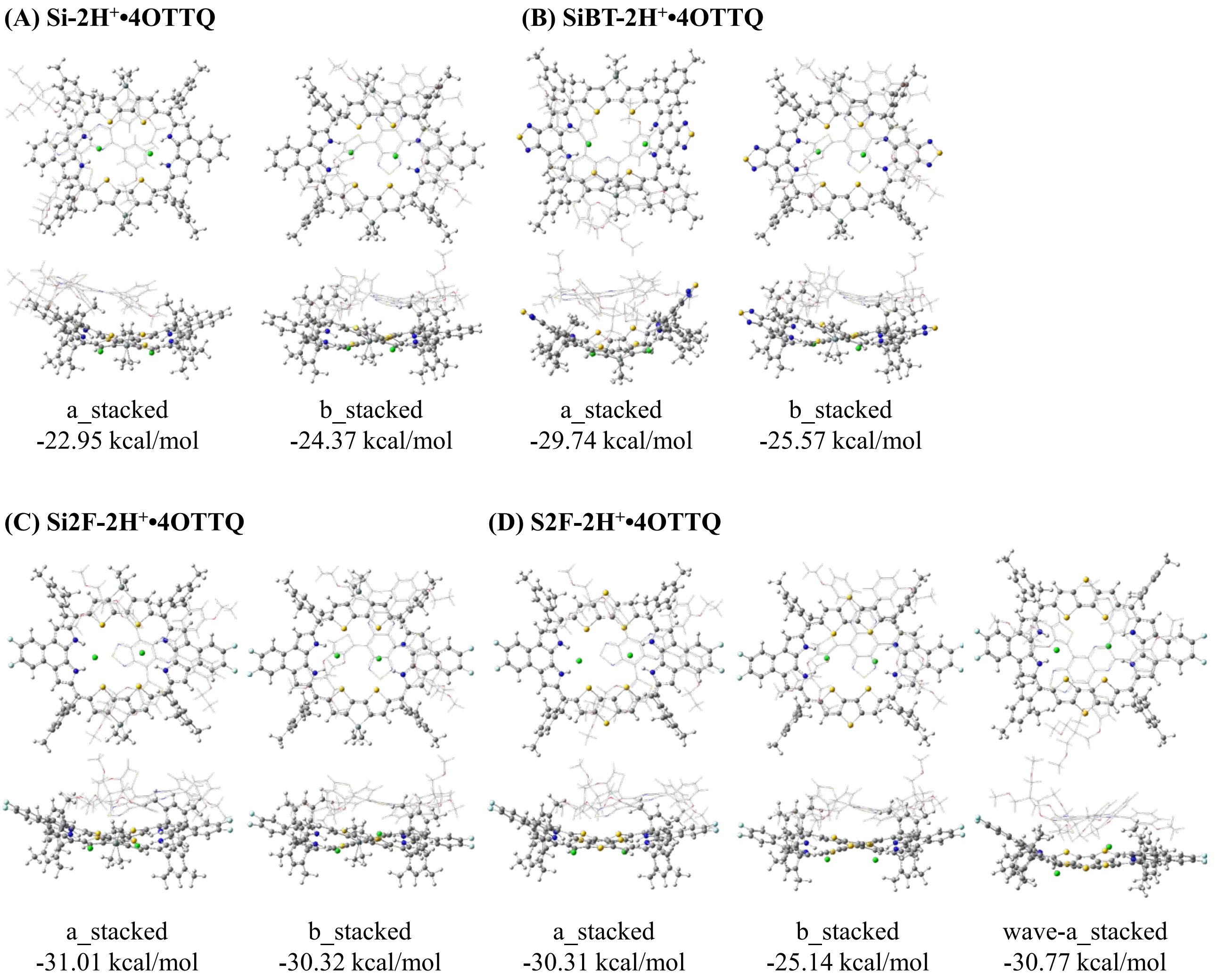

Scheme S4. Optimized structures of (A) **Si-2H$^+$**, (B) **SiBT-2H$^+$**, (C) **Si2F-2H$^+$**, and (D) **S2F-2H$^+$** with **4OTTQ** complexes at the theoretical level of B3LYP-D3/6-31G*. For clarity, dye molecules are represented in ball-and-stick format and **4OTTQ** is represented in stick format. The upper layer is a top view and the bottom layer is a side view. The energy values are BSSE corrected binding energies of the two fragments calculated at the M06-2X/6-31+G* level.

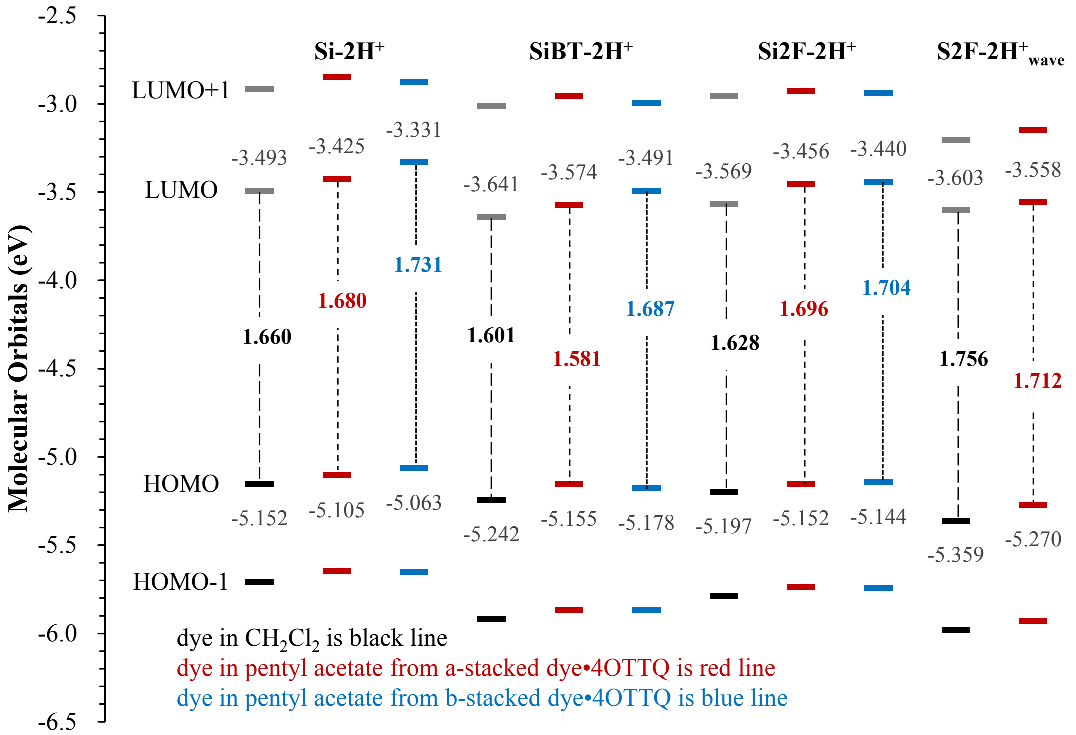

Figure S7. Molecular orbital energy diagram of **Si-2H$^+$**, **SiBT-2H$^+$**, **Si2F-2H$^+$**, **S2F-2H$^+$** wave-shaped molecules calculated at PBE38/6-31G* level in CH_2Cl_2 or pentyl acetate modeled with CPCM.

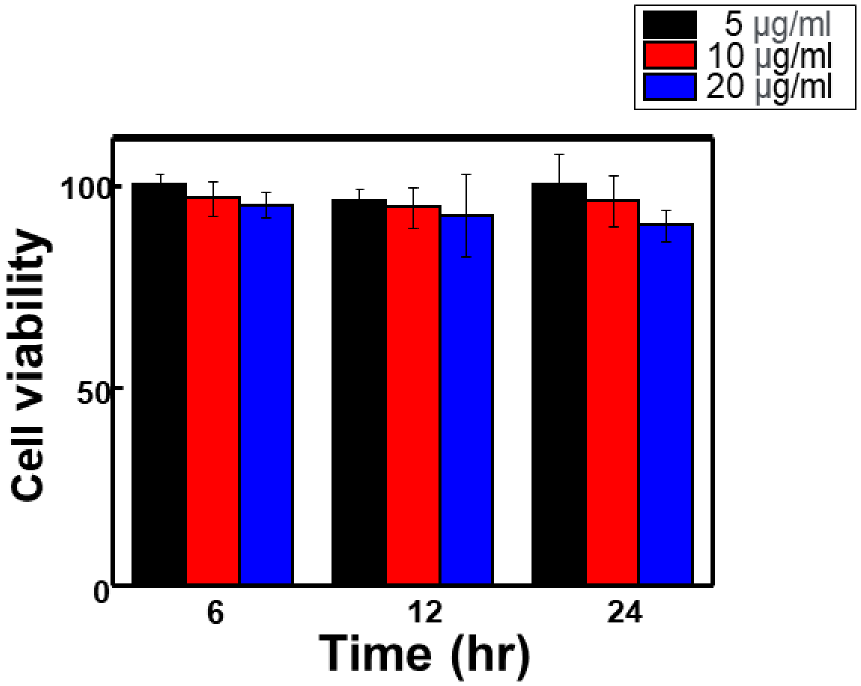

Figure S8. Cytotoxicity results on HeLa cells evaluated by MTT assays. The cells were incubated with **SiBT-2H$^+$** Pdots of different concentrations (5-20 μg/mL) at various incubation times (6-24 h). Each statistical analysis had a sample size of five (n = 5).

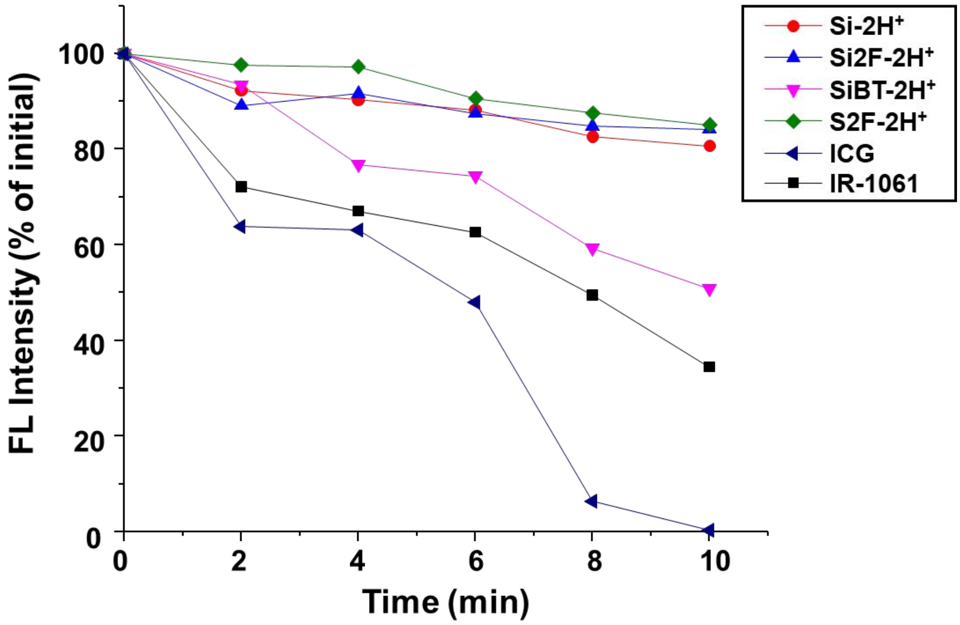

Figure S9. Photostability of **Si-2H$^+$** (red line), **Si2F-2H$^+$** (blue line), **SiBT-2H$^+$** (pink line), **S2F-2H$^+$** (green line), ICG (purple line) and IR-1061 (black line) dissolved in CH_2Cl_2 under continuous 254 nm UV irradiation. The concentration is 0.01 mg mL^{-1} for all probes.

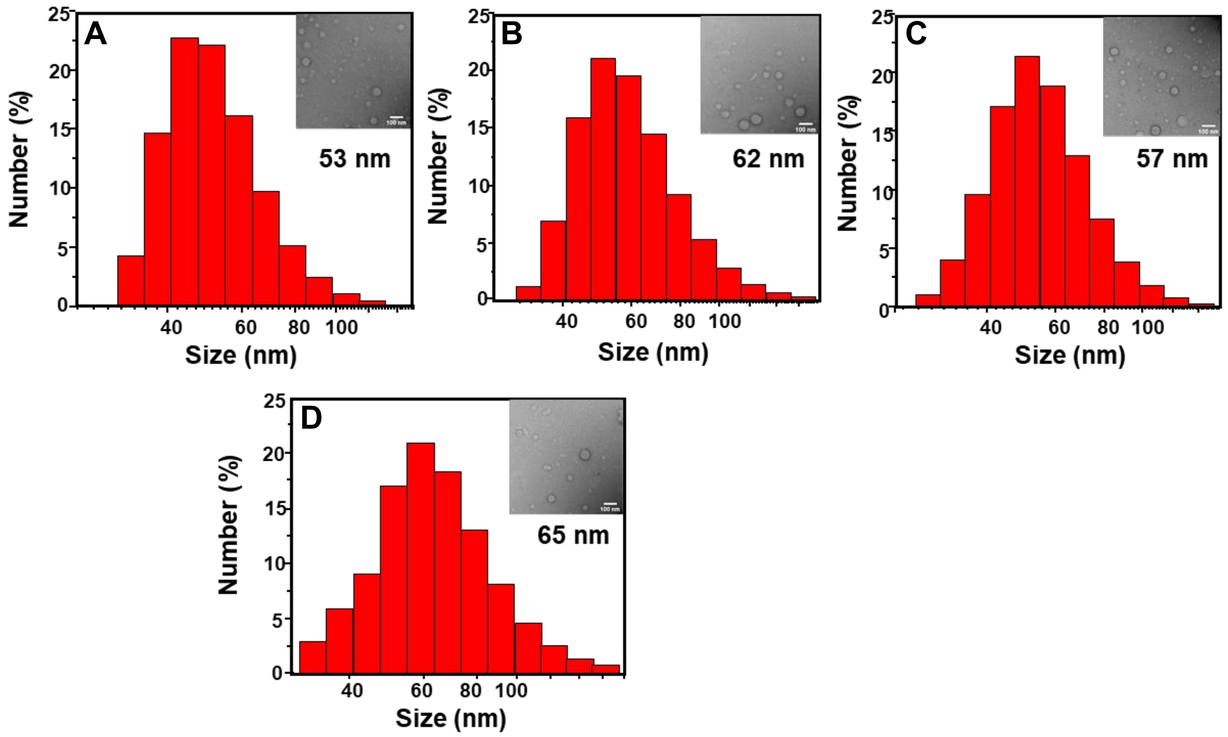

Figure S10. Hydrodynamic diameters of (A) **Si-2H$^+$**, (B) **SiBT-2H$^+$**, (C) **Si2F-2H$^+$**, and (D) **S2F-2H$^+$** Pdots. The insets on the images represent their corresponding TEM images. The scale bars are 100 nm. The average sizes of the Pdots were 53±13 nm, 62±15 nm, 57±12 nm, and 65±10 nm for **Si-2H$^+$**, **SiBT-2H$^+$**, **Si2F-2H$^+$**, and **S2F-2H$^+$** Pdots, respectively. The means ± standard deviations were used to express statistical data and continuous variables of DLS measurements. Each statistical analysis had a sample size of three (n = 3).

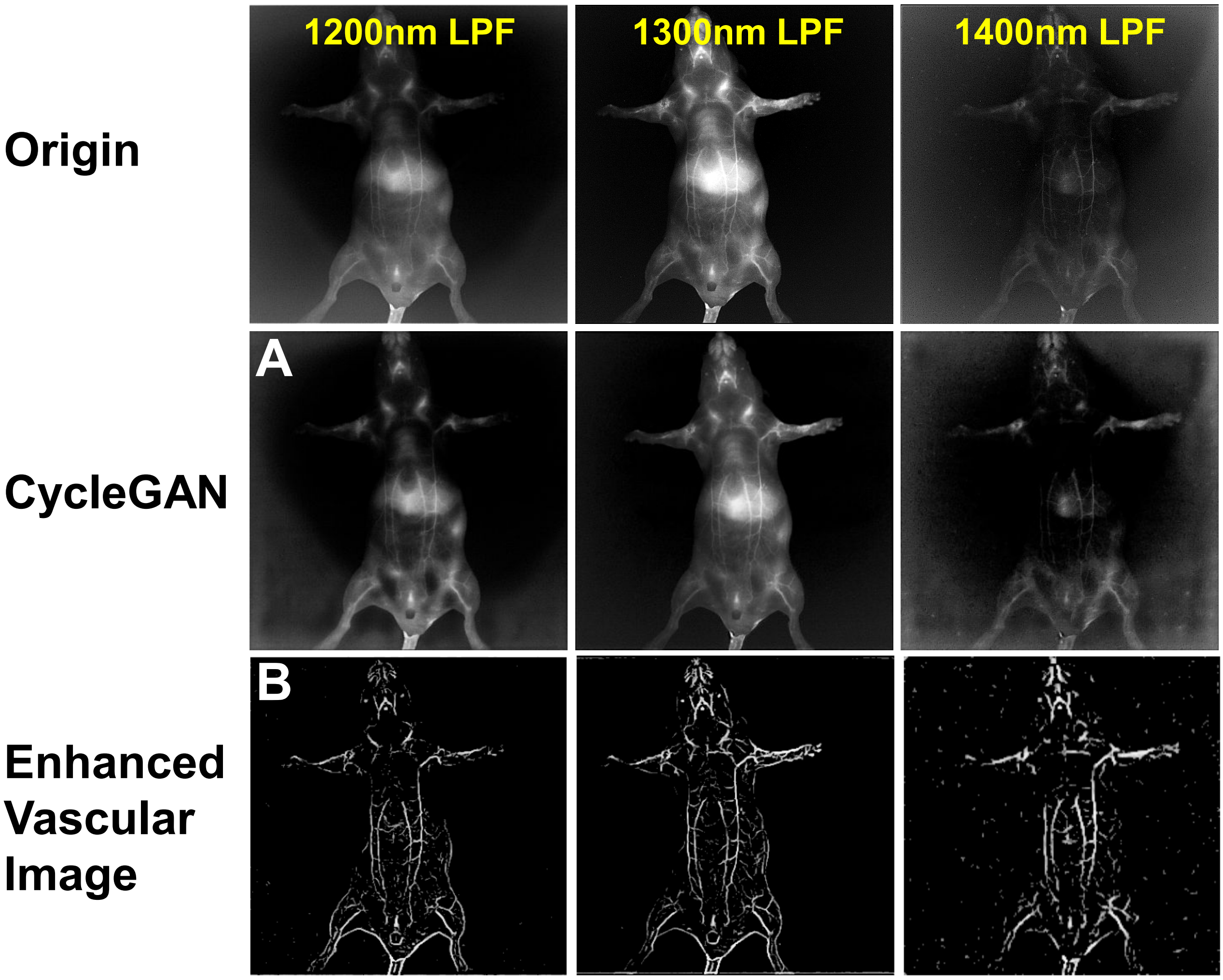

Figure S11. AI-assisted NIR-II imaging using (A) CycleGAN and (B) Frangi vesselness filter.

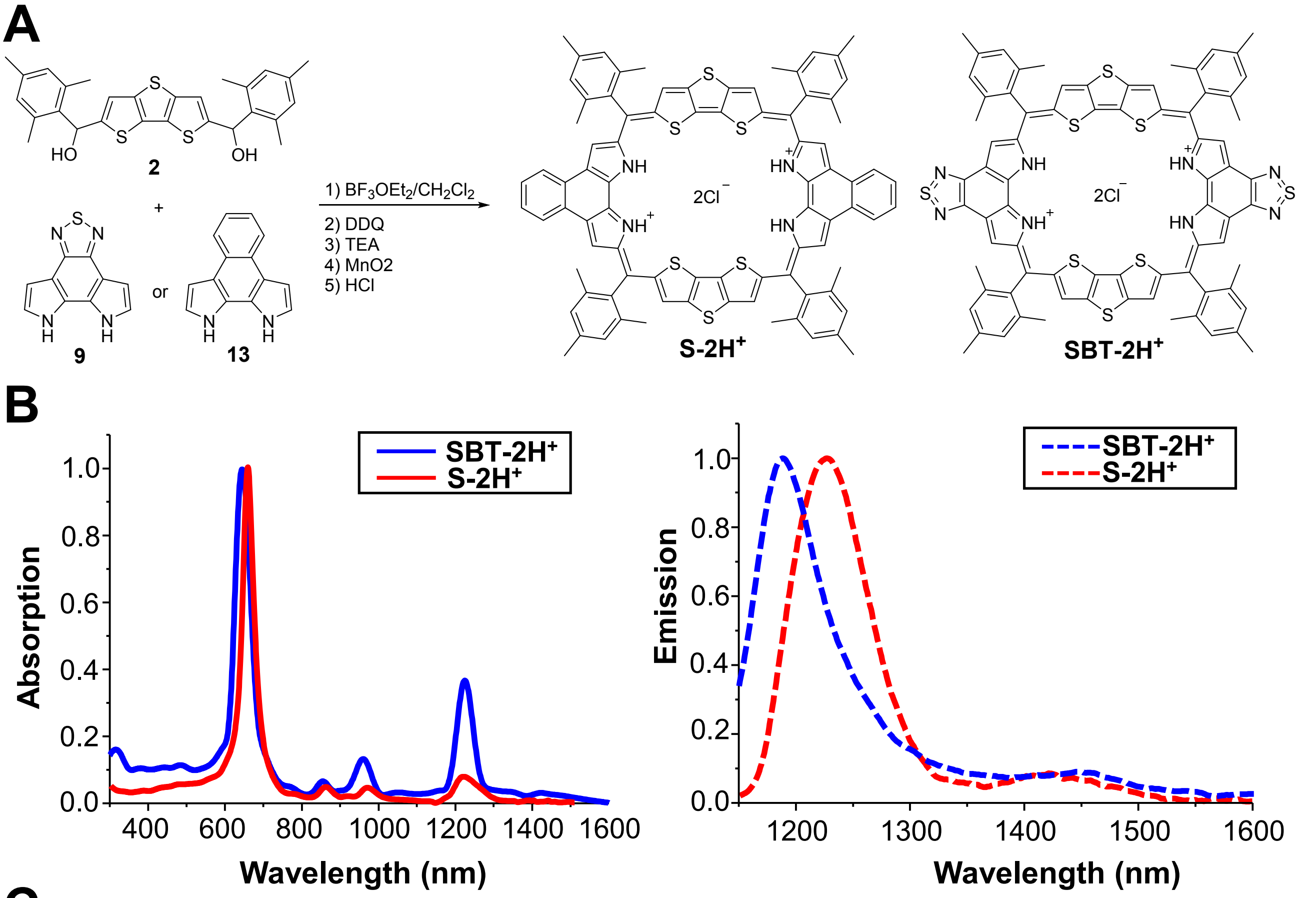

C

Compound	Soret band λ_{max}^{abs} (nm)	Q band λ_{max}^{abs} (nm)	λ_{max}^{em} (nm)	S_2-Stokes shift (nm)	ε^{max} (M^{-1} cm^{-1})	Φ(%)
S-2H$^+$	655	856, 960, 1212	1248	593	2.99× 10^4	3.44
SBT-2H$^+$	646	856, 962, 1228	1187	481	1.23× 10^5	0.43
S2F-2H$^+$	656	864, 971, 1237	1235	579	3.57× 10^4	6.14
Si-2H$^+$	664	885, 1035, 1259	1269	605	1.52× 10^4	1.48
SiBT-2H$^+$	666	900, 1075, 1279	1290	624	3.71× 10^4	1.10
Si2F-2H$^+$	670	899, 1013, 1272	1281	611	1.01× 10^4	1.23

Figure S12. (A) Synthetic routes of **S-2H$^+$** and **SBT-2H$^+$** and (B) their corresponding absorption (left) and emission (right) spectra. (C) Summary of optical properties of NIR-II emissive porphyrinoids studies in this work.

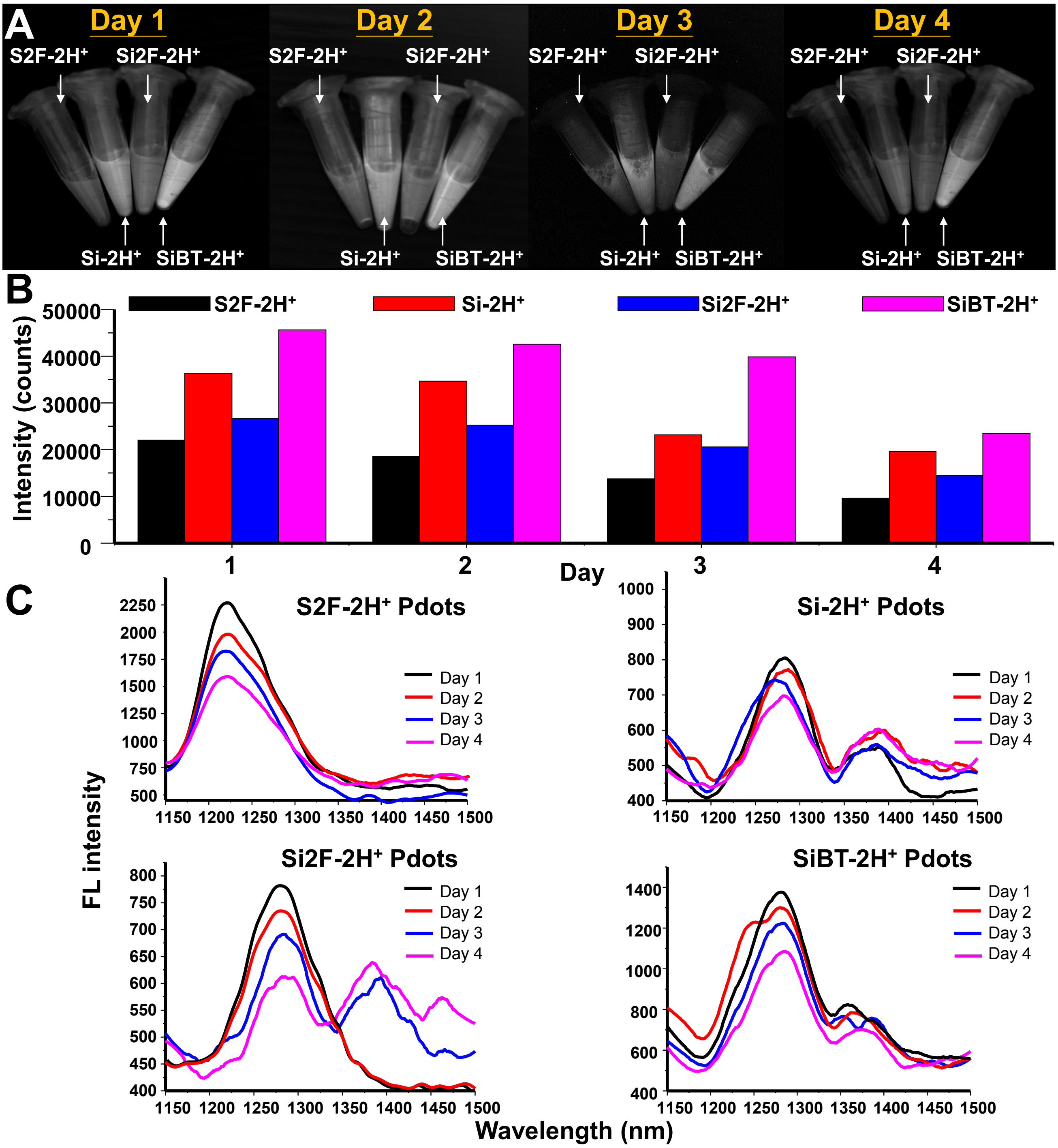

Figure S13. (A) Photographs of porphyrinoid Pdots (left to right: **S2F-2H**$^+$, **Si2H-2H**$^+$, **Si2F-2H**$^+$, **SiBT-2H**$^+$ Pdots) in water under 1064 nm laser (100 mW cm^{-2}) with a 1300 nm long-pass filter in different days. (B) Their corresponding mean fluorescence intensities and (C) emission spectra.

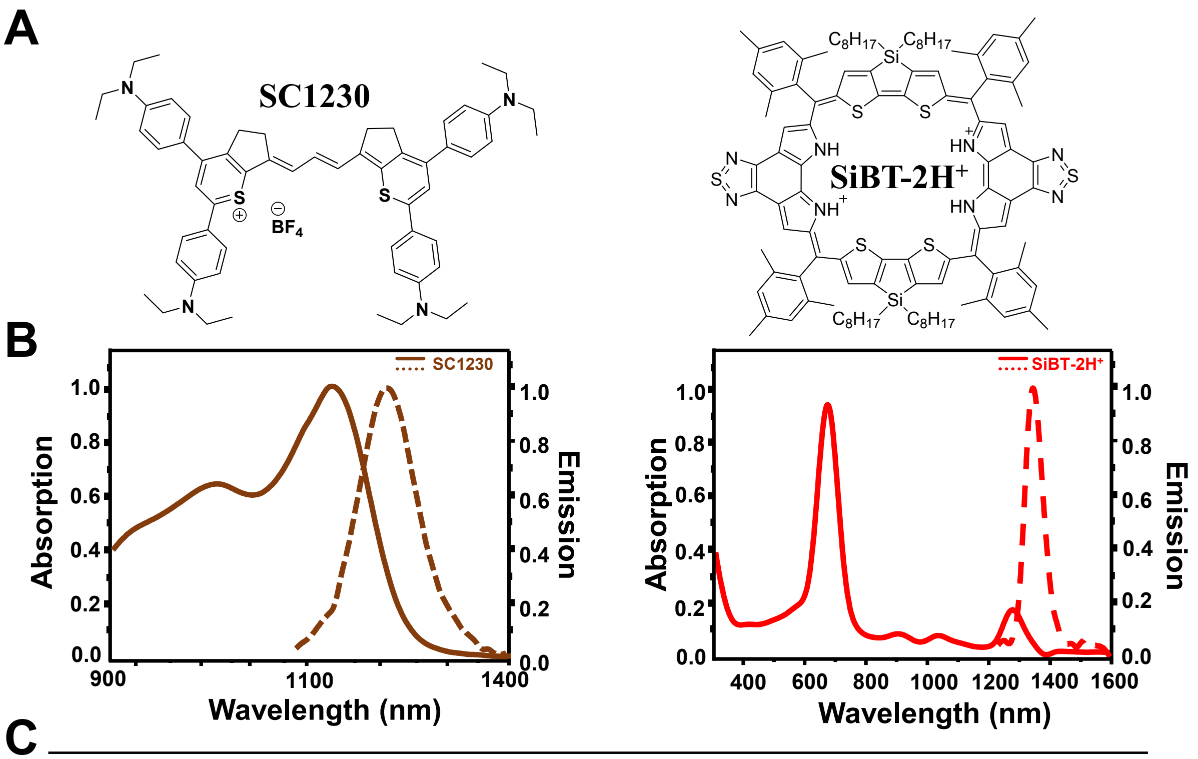

C

compound	λ_{abs} (nm)	λ_{em} (nm)	Stokes shift (nm)	Φ (%)
**SC1230**	1030, 1175	1230	55	0.20
**SiBT-2H$^+$**	666	1290	624	1.10

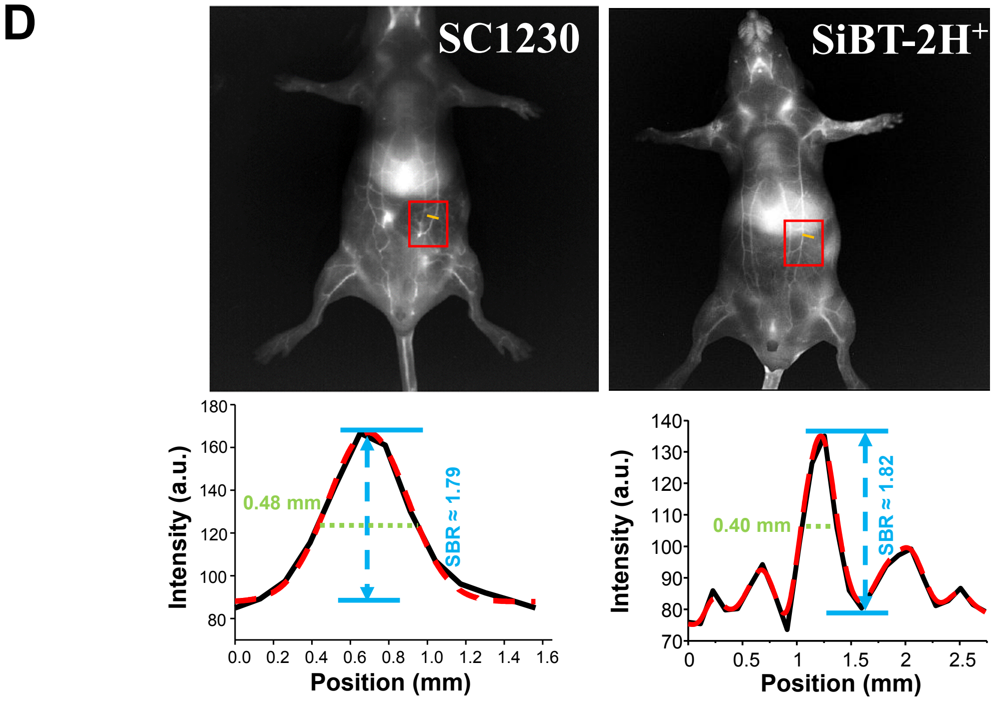

Figure S14. (A) Chemical structures of polymethine, **SC1230** and porphyrinoid, **SBT-2H$^+$**. (B) Their corresponding absorption (solid lines) and emission (dashed lines) spectra. (C) Summary of their optical properties. (D) Whole-body fluorescence imaging of vascular structures in mice at the supine position injected by **SC1230** nanoprobes (left) and **SiBT-2H$^+$** Pdots (right) at the same concentration of 2 mg/mL with a 1300 nm long-pass filter under 1064 nm laser excitation.

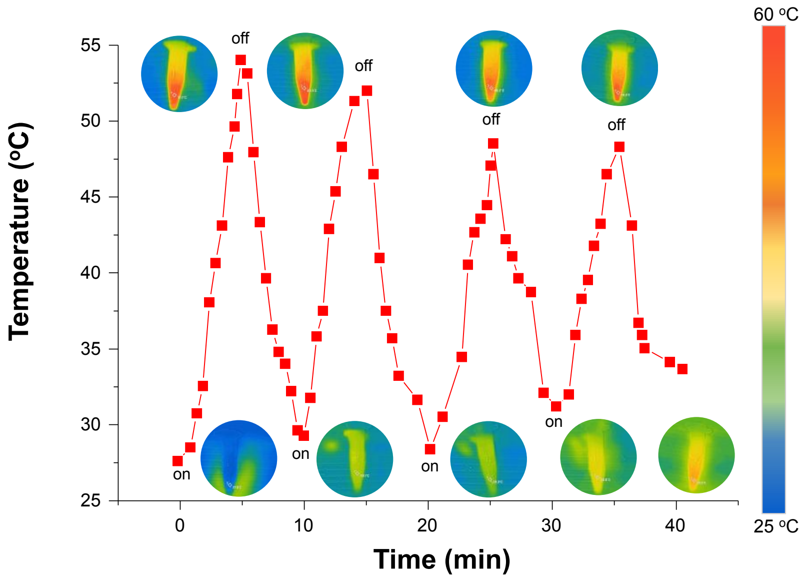

Figure S15. Temperature variations of the **SiBT-2H$^+$** Pdots under 1064 nm laser irradiation at a power density of 1.5 W cm^{-2} for five light on/off cycles of laser irradiation.

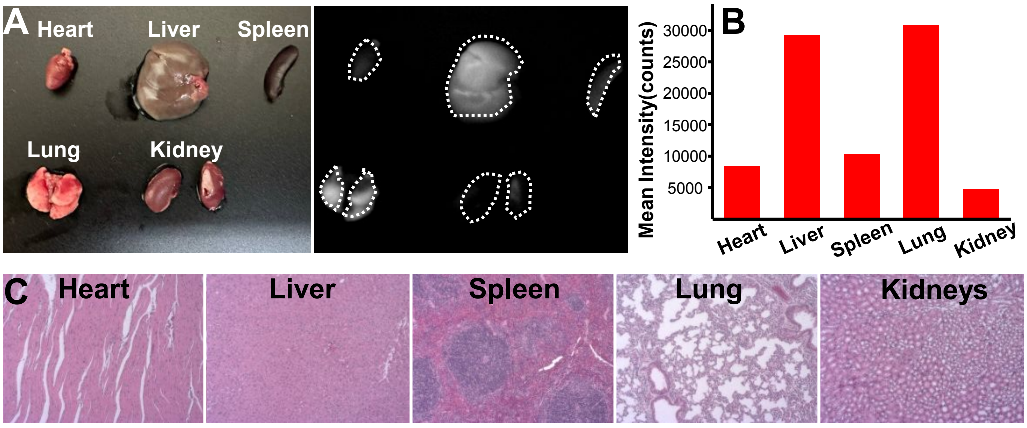

D

Test Item	PBS	Pdots	Test Item	PBS	Pdots
ALanine aminotransferase (U/L)	44	33	ASpartate aminotransferase (U/L)	77	68
Liver and Gallbladder-Total Bilirubin (mg/dL)	0.33	0.7	Gamma Glutamyl Transpeptidase (U/L)	3	3
Blood Urea Nitrogen (mg/dL)	23	27	Alkaline Phosphatase (U/L)	77	70
Creatinine (mg/dL)	0.2	0.4	Calcium (mg/dL)	10.8	10.6
Phosphate (mg/dL)	7	7.6	Amylase (U/L)	509	512
Glucose (mg/dL)	115	150	Globulin (g/dL)	2.9	3
Total protein (g/dL)	6.4	6.4	Albumin/Globulin	1	1
Albumin (g/dL)	2.9	3	Blood Urea Nitrogen /Creatinine	60	66

Figure S16. (A) Biodistribution of **SiBT-2H$^+$** Pdots in major excised organs at 24 h post-injection in which the bright-field image (left) and NIR-II fluorescence imaging (right) of different organs were shown. (B) The corresponding quantitative mean fluorescence intensities in organs. (C) H&E

staining of organ sections from Pdot-treated mouse at 24 h post-injection. (D) Serum biochemical analysis of PBS-treated and Pdot-treated mice at 24 h post-injection.

Supporting References

(1) Sarma, T.; Kim, G.; Sen, S.; Cha, W.-Y.; Duan, Z.; Moore, M. D.; Lynch, V. M.; Zhang, Z.; Kim, D.; Sessler, J. L. *Journal of the American Chemical Society* **2018**, *140*, 12111.

(2) Chen, C.-P.; Huang, Y.-C.; Liou, S.-Y.; Wu, P.-J.; Kuo, S.-Y.; Chan, Y.-H. *ACS Applied Materials & Interfaces* **2014**, *6*, 21585.

(3) Steckler, T. T.; Abboud, K. A.; Craps, M.; Rinzler, A. G.; Reynolds, J. R. *Chemical Communications* **2007**, 4904.

(4) Firmansyah, D.; Hong, S.-J.; Dutta, R.; He, Q.; Bae, J.; Jo, H.; Kim, H.; Ok, K. M.; Lynch, V. M.; Byon, H. R.; Sessler, J. L.; Lee, C.-H. *Chemistry – A European Journal* **2019**, *25*, 3525.

(5) Liu, H.-Y.; Wu, P.-J.; Kuo, S.-Y.; Chen, C.-P.; Chang, E.-H.; Wu, C.-Y.; Chan, Y.-H. *Journal of the American Chemical Society* **2015**, *137*, 10420.

(6) Li, H.; Tam, T. L.; Lam, Y. M.; Mhaisalkar, S. G.; Grimsdale, A. C. *Organic Letters* **2011**, *13*, 46.

(7) Tsai, W.-K.; Wang, C.-I.; Liao, C.-H.; Yao, C.-N.; Kuo, T.-J.; Liu, M.-H.; Hsu, C.-P.; Lin, S.-Y.; Wu, C.-Y.; Pyle, J. R.; Chen, J.; Chan, Y.-H. *Chemical Science* **2019**, *10*, 198.

(8) Ma, Z.; Wang, F.; Wang, W.; Zhong, Y.; Dai, H. *Proceedings of the National Academy of Sciences* **2021**, *118*, e2021446118.

(9) Wang, F.; Zhong, Y.; Bruns, O.; Liang, Y.; Dai, H. *Nature Photonics* **2024**, *18*, 535.

(10) Zhu, J.-Y.; Park, T.; Isola, P.; Efros, A. A. In *Proceedings of the IEEE international conference on computer vision* 2017, p 2223.

(11) Frangi, A. F.; Niessen, W. J.; Vincken, K. L.; Viergever, M. A. In *Medical Image Computing and Computer-Assisted Intervention — MICCAI'98*; Wells, W. M., Colchester, A., Delp, S., Eds.; Springer Berlin Heidelberg: Berlin, Heidelberg, 1998, p 130.

(12) Grimme, J. Antony, S. Ehrlich and H. Krieg, *J. Chem. Phys.* **2010**, *132*, 154104.

(13) Barone, V.; Cossi, M. *J. Phys. Chem. A* **1998**, 102, 1995.
